# Supplementary material for: The Evolution and Characterization of the RNA Interference Pathways in Lophotrochozoa
Source: Genome Biol Evol. 2024 May 7;16(5):evae098. doi: 10.1093/gbe/evae098 (PMC11114477; doi:10.1093/gbe/evae098)
Supplement: evae098_Supplementary_Data [file evae098_supplementary_data.zip › Supplementary_materials.zip!/selected_miRNAs/NoGe_selected_miRNAs/mirdeep_pdf_files/NMRB01001187.1_Notospermus_geniculatus_strain_Ushimado_scaffold1187,_whole_genome_shotgun_sequence_26007.pdf]

Provisional ID	:	NMRB01001187.1_Notospermus_geniculatus_strain_Ushimado_scaffold1187,_whole_genome_shotgun_sequence_26007
Score total	:	2368407.3
Score for star read(s)	:	3.9
Score for read counts	:	2368398.9
Score for mfe	:	1.6
Score for randfold	:	0
Score for cons. seed	:	3
Total read count	:	4645524
Mature read count	:	4645442
Loop read count	:	0
Star read count	:	82

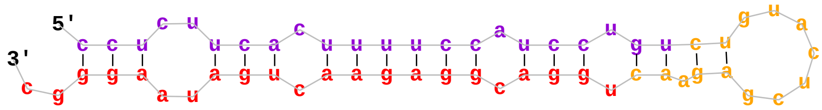

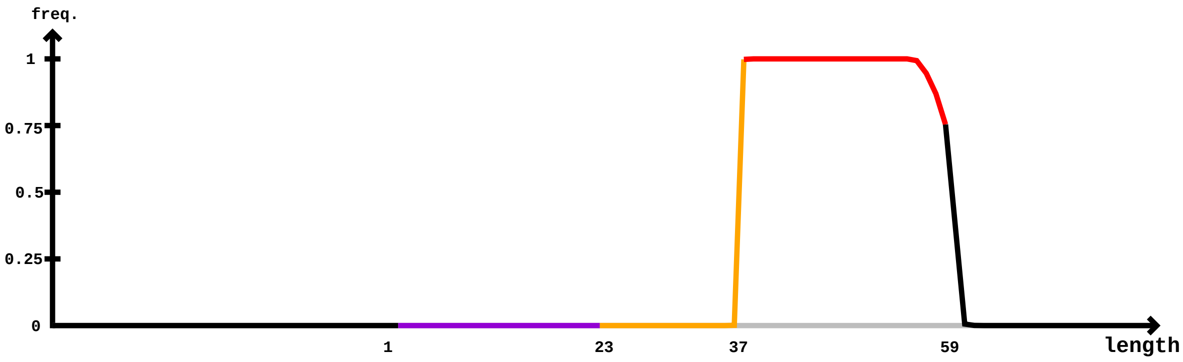

Star Mature

```
5'- cauuugugacaugagaaguaaguuugucucguguccucuucacuuuuccauccugucuguacucgagaacuggacggagaacugauaagggcauagggcagaccacaucaaca  -3'   obs
    cauuugugacaugagaaguaaguuugucucguguccucuucacuuuuccauccugucuguacucgagaacuggacggagaacugauaagggcauagggcagaccacaucaaca         exp
    ..............((.((..((((((((.(((((((..(((.((((((.(((.((((.......)).)).))).)))))).)))..))))))).)))))))).)).))....   reads    mm      sample
    ..................................ccucuucacuuuuccauccugu.........................................................   8        0       Mm3
    ..................................ccucuucacuuuuccauccugucug......................................................   1        0       Mm3
    ...............................................................cgagaacuggacggagaacugauaagggc.....................   1        0       Mm3
    ....................................................................Ucuggacggagaacugauaagggc.....................   6        1       Mm3
    .....................................................................cuggacggagaacugaua..........................   1        0       Mm3
    .....................................................................cuggacggagaacugauaa.........................   3        0       Mm3
    .....................................................................cuggacggagaacugauaaA........................   5        1       Mm3
    .....................................................................cuggacggagaacugauaag........................   13       0       Mm3
    .....................................................................cuggacggagaacugauaagA.......................   9        1       Mm3
    .....................................................................cuggacggagaacugauaagg.......................   18       0       Mm3
    .....................................................................cuggaNggagaacugauaagg.......................   1        1       Mm3
    .....................................................................cuggacggagaacugauUagg.......................   1        1       Mm3
    .....................................................................cuggacggagaacugauaaggC......................   1        1       Mm3
    .....................................................................cuggacggagaacugauaGggg......................   1        1       Mm3
    .....................................................................cuggacggagaacugauaaggU......................   3        1       Mm3
    .....................................................................cuggacggagaacugauaaggg......................   33       0       Mm3
    .....................................................................cuggacggagaacugauCaggg......................   2        1       Mm3
    .....................................................................cuggacggagaacugauaaggA......................   4        1       Mm3
    .....................................................................cuggacggagaacugauaagggA.....................   2        1       Mm3
    .....................................................................cuggacggagaacugauaagggc.....................   112      0       Mm3
    .....................................................................cuggCcggagaacugauaagggc.....................   1        1       Mm3
    .....................................................................cAggacggagaacugauaagggc.....................   2        1       Mm3
    .....................................................................cuggacggagaacugauaagggU.....................   2        1       Mm3
    .....................................................................cuggacggagaacugauaagggca....................   7        0       Mm3
    .....................................................................cuggacggagaUcugauaagggca....................   2        1       Mm3
    ......................................................................uggacggagaaUugauaa.........................   1        1       Mm3
    ......................................................................uggaUggagaacugauaa.........................   2        1       Mm3
    ......................................................................uggacggagaacugaAaa.........................   66       1       Mm3
    ......................................................................uggacggagaGcugauaa.........................   3        1       Mm3
    ......................................................................uggacggaCaacugauaa.........................   1        1       Mm3
    ......................................................................uggacggagCacugauaa.........................   1        1       Mm3
    ......................................................................uggacggagaacugauGa.........................   2        1       Mm3
    ......................................................................uggacggagaacugauCa.........................   6        1       Mm3
```

Star Mature

```
cauuugugacaugagaaguaaguuugucucguguccucuucacuuuuccauccuguucuguacucgagaacuggacggagaacugauaagggcauagggcagaccacaucaaca
......................................................................uggCcggagaacugauaa.........................   1      1   Mm3
......................................................................Nggacggagaacugauaa.........................   2      1   Mm3
......................................................................uggacggagaacugauaC.........................   4      1   Mm3
......................................................................uggacggUgaacugauaa.........................   1      1   Mm3
......................................................................uggacggagaacugauaU.........................   62     1   Mm3
......................................................................uggacggagaacugauaG.........................   13     1   Mm3
......................................................................Aggacggagaacugauaa.........................   2      1   Mm3
......................................................................uggacUgagaacugauaa.........................   1      1   Mm3
......................................................................uggacggagaacugauUa.........................   5      1   Mm3
......................................................................uggacggagaacugauaa.........................   2074   0   Mm3
......................................................................uggacggagaacugaCaa.........................   2      1   Mm3
......................................................................uggacggagaacCgauaa.........................   1      1   Mm3
......................................................................uggacggagaCcugauaa.........................   1      1   Mm3
......................................................................uggacggagGacugauaag........................   9      1   Mm3
......................................................................uggacggagaacugauaCg........................   30     1   Mm3
......................................................................uggacggagaacuAauaag........................   4      1   Mm3
......................................................................uggacggagaacugauUag........................   14     1   Mm3
......................................................................uggacggagaacCgauaag........................   1      1   Mm3
......................................................................uggCcggagaacugauaag........................   1      1   Mm3
......................................................................uggacggUgaacugauaag........................   3      1   Mm3
......................................................................uggacggagaacugCuaag........................   1      1   Mm3
......................................................................uggacggagaCcugauaag........................   14     1   Mm3
......................................................................Nggacggagaacugauaag........................   3      1   Mm3
......................................................................uggacggagaacugauaaC........................   15     1   Mm3
......................................................................uAgacggagaacugauaag........................   3      1   Mm3
......................................................................Aggacggagaacugauaag........................   11     1   Mm3
......................................................................uggacggagaacugauaaA........................   5374   1   Mm3
......................................................................uggacggagaacuUauaag........................   4      1   Mm3
......................................................................uggacggagaacugaAaag........................   3      1   Mm3
......................................................................uUgacggagaacugauaag........................   2      1   Mm3
......................................................................uggacggagCacugauaag........................   1      1   Mm3
......................................................................uggGcggagaacugauaag........................   1      1   Mm3
......................................................................Cggacggagaacugauaag........................   2      1   Mm3
......................................................................uggacggagaacugauGag........................   10     1   Mm3
......................................................................uggacggagaacugUuaag........................   1      1   Mm3
......................................................................uggacggagaacugauaag........................   8431   0   Mm3
......................................................................uggacggagaacugauaUg........................   30     1   Mm3
......................................................................uggacggagaacugauaGg........................   40     1   Mm3
......................................................................uggacggaUaacugauaag........................   2      1   Mm3
......................................................................uggacAgagaacugauaag........................   4      1   Mm3
......................................................................uggacggagaacugaCaag........................   1      1   Mm3
......................................................................uggacggagaGcugauaag........................   30     1   Mm3
......................................................................uggacggagaaUugauaag........................   2      1   Mm3
......................................................................uggacggagUacugauaag........................   4      1   Mm3
......................................................................uggacUgagaacugauaag........................   2      1   Mm3
......................................................................uggaAggagaacugauaag........................   1      1   Mm3
......................................................................uggacggagaaAugauaag........................   1      1   Mm3
......................................................................uggUcggagaacugauaag........................   1      1   Mm3
......................................................................uggacggagaacugauCag........................   11     1   Mm3
......................................................................ugAacggagaacugauaag........................   4      1   Mm3
......................................................................uggacggagaacugauaaU........................   1351   1   Mm3
......................................................................uggacggGgaacugauaag........................   3      1   Mm3
......................................................................uggGcggagaacugauaagg.......................   5      1   Mm3
......................................................................uggacggagGacugauaagg.......................   27     1   Mm3
......................................................................uggacNgagaacugauaagg.......................   3      1   Mm3
......................................................................uggacggagaacuUauaagg.......................   1      1   Mm3
......................................................................uggacggagaacGgauaagg.......................   2      1   Mm3
......................................................................uggacggaUaacugauaagg.......................   1      1   Mm3
......................................................................uggacggCgaacugauaagg.......................   2      1   Mm3
......................................................................uggacggagaacugauaaUg.......................   2      1   Mm3
......................................................................uggacgCagaacugauaagg.......................   3      1   Mm3
......................................................................uggacggaCaacugauaagg.......................   1      1   Mm3
......................................................................uggacUgagaacugauaagg.......................   11     1   Mm3
......................................................................uAgacggagaacugauaagg.......................   11     1   Mm3
......................................................................uggacggagaaUugauaagg.......................   3      1   Mm3
......................................................................uggacggGgaacugauaagg.......................   2      1   Mm3
......................................................................uUgacggagaacugauaagg.......................   7      1   Mm3
......................................................................uggacggagaacugauaGgg.......................   91     1   Mm3
......................................................................uggacggagaacugUuaagg.......................   3      1   Mm3
......................................................................ugCacggagaacugauaagg.......................   2      1   Mm3
```

Star Mature

```
cauuugugacaugagaaguaaguuugucucguguccucuucacuuuuccauccuguCuguacucgagaacuggacggagaacugauaagggcauagggcagaccacaucaaca
......................................................................Gggacggagaacugauaagg.......................   1      1   Mm3
......................................................................uggacgAagaacugauaagg.......................   2      1   Mm3
......................................................................uggaUggagaacugauaagg.......................   7      1   Mm3
......................................................................uggacggagaacCgauaagg.......................   1      1   Mm3
......................................................................uggacAgagaacugauaagg.......................   2      1   Mm3
......................................................................uggacggagaacugGuaagg.......................   1      1   Mm3
......................................................................uggacggagaacugauaaAg.......................   91     1   Mm3
......................................................................uggacggagaCcugauaagg.......................   76     1   Mm3
......................................................................uggCcggagaacugauaagg.......................   3      1   Mm3
......................................................................uCgacggagaacugauaagg.......................   1      1   Mm3
......................................................................uggacggagaaAugauaagg.......................   1      1   Mm3
......................................................................Aggacggagaacugauaagg.......................   140    1   Mm3
......................................................................ugUacggagaacugauaagg.......................   3      1   Mm3
......................................................................uggacggaAaacugauaagg.......................   2      1   Mm3
......................................................................uggacggagaacugauUagg.......................   12     1   Mm3
......................................................................Cggacggagaacugauaagg.......................   4      1   Mm3
......................................................................uggacggagaacugauaCgg.......................   58     1   Mm3
......................................................................uggacggagCacugauaagg.......................   5      1   Mm3
......................................................................uggacggagaacuAauaagg.......................   1      1   Mm3
......................................................................uggacCgagaacugauaagg.......................   1      1   Mm3
......................................................................uggacggagaacugauaagg.......................   14584  0   Mm3
......................................................................uggacggagaacugauaagC.......................   10     1   Mm3
......................................................................uggacggagaacugauCagg.......................   31     1   Mm3
......................................................................Nggacggagaacugauaagg.......................   6      1   Mm3
......................................................................uggacggagUacugauaagg.......................   2      1   Mm3
......................................................................uggacggagaacugauGagg.......................   21     1   Mm3
......................................................................uggUcggagaacugauaagg.......................   4      1   Mm3
......................................................................uggacggagaacugauaagU.......................   617    1   Mm3
......................................................................uggacggagaGcugauaagg.......................   140    1   Mm3
......................................................................uggacggagaacugauaagA.......................   3650   1   Mm3
......................................................................uggacgUagaacugauaagg.......................   1      1   Mm3
......................................................................uggacggUgaacugauaagg.......................   6      1   Mm3
......................................................................uggacggagaacugauaUgg.......................   64     1   Mm3
......................................................................uNgacggagaacugauaagg.......................   1      1   Mm3
......................................................................uggacggagaacugauaaggU......................   657    1   Mm3
......................................................................Gggacggagaacugauaaggg......................   1      1   Mm3
......................................................................uggacggagaacugGuaaggg......................   1      1   Mm3
......................................................................uggacggagaacugauaaggA......................   4536   1   Mm3
......................................................................uggacggagaacugauaagAg......................   28     1   Mm3
......................................................................uggacggagaacugauaaUgg......................   3      1   Mm3
......................................................................uggacggagaacugaCaaggg......................   1      1   Mm3
......................................................................uggacgUagaacugauaaggg......................   5      1   Mm3
......................................................................uggacNgagaacugauaaggg......................   2      1   Mm3
......................................................................uggacggagCacugauaaggg......................   5      1   Mm3
......................................................................Nggacggagaacugauaaggg......................   13     1   Mm3
......................................................................uggacggagaacugauaagUg......................   2      1   Mm3
......................................................................uggUcggagaacugauaaggg......................   3      1   Mm3
......................................................................uggacggagaacCgauaaggg......................   4      1   Mm3
......................................................................Aggacggagaacugauaaggg......................   102    1   Mm3
......................................................................uggacCgagaacugauaaggg......................   2      1   Mm3
......................................................................uggacggagaaAugauaaggg......................   2      1   Mm3
......................................................................uggacggCgaacugauaaggg......................   4      1   Mm3
......................................................................uggacggagaacugauaaAgg......................   3      1   Mm3
......................................................................uUgacggagaacugauaaggg......................   5      1   Mm3
......................................................................uCgacggagaacugauaaggg......................   1      1   Mm3
......................................................................uAgacggagaacugauaaggg......................   9      1   Mm3
......................................................................uggacggagaacuAauaaggg......................   6      1   Mm3
......................................................................uggacggagaacugauaagCg......................   1      1   Mm3
......................................................................uggacggagaacugauaaggC......................   89     1   Mm3
......................................................................uggacgNagaacugauaaggg......................   2      1   Mm3
......................................................................uggacggagaacugauGaggg......................   40     1   Mm3
......................................................................uggacggGgaacugauaaggg......................   11     1   Mm3
......................................................................uggacggagaacugauUaggg......................   38     1   Mm3
......................................................................ugAacggagaacugauaaggg......................   7      1   Mm3
......................................................................uggacggagaaUugauaaggg......................   7      1   Mm3
......................................................................ugUacggagaacugauaaggg......................   3      1   Mm3
......................................................................uggacggagaacugauaaggg......................   27231  0   Mm3
......................................................................uggCcggagaacugauaaggg......................   3      1   Mm3
......................................................................uggacggUgaacugauaaggg......................   15     1   Mm3
......................................................................uggacggagaacugauaGggg......................   205    1   Mm3
```

Star **Mature**

cauuugugacaugagaaguaaguuugucucguguccucuucacuuuuccauccuguc uguacucgagaacuggacggagaacugauaagggcauagggcagaccacaucaaca			
....uggacggagaacugauCaggg......................	60	1	Mm3
....uggacggagaacugUuaaggg......................	5	1	Mm3
....uggaAggagaacugauaaggg......................	2	1	Mm3
....Cggacggagaacugauaaggg......................	11	1	Mm3
....uggGcggagaacugauaaggg......................	6	1	Mm3
....uggacggagaacAgauaaggg......................	3	1	Mm3
....uggacggagGacugauaaggg......................	35	1	Mm3
....uggacUgagaacugauaaggg......................	13	1	Mm3
....uggacgAagaacugauaaggg......................	5	1	Mm3
....uggacAgagaacugauaaggg......................	5	1	Mm3
....uggacggagaacugaAaaggg......................	1	1	Mm3
....uggacggagaacugauaCggg......................	118	1	Mm3
....uggacggaUaacugauaaggg......................	6	1	Mm3
....uggacggagaGcugauaaggg......................	145	1	Mm3
....uggacggagaacugCuaaggg......................	1	1	Mm3
....uggacggaAaacugauaaggg......................	1	1	Mm3
....uggacggagaacuCauaaggg......................	2	1	Mm3
....uggacggaCaacugauaaggg......................	1	1	Mm3
....uggacggagaCcugauaaggg......................	71	1	Mm3
....uggaUggagaacugauaaggg......................	3	1	Mm3
....uggacggagaacuUauaaggg......................	5	1	Mm3
....uggacggagaacugauaUggg......................	74	1	Mm3
....uggacggagUacugauaaggg......................	9	1	Mm3
....Gggacggagaacugauaagggc.....................	15	1	Mm3
....uggacggagaacugCuaagggc.....................	9	1	Mm3
....ugCacggagaacugauaagggc.....................	24	1	Mm3
....uggacggagaacugauaNgggc.....................	2	1	Mm3
....uggCcggagaacugauaagggc.....................	70	1	Mm3
....uggacggagaacugauaaCggc.....................	7	1	Mm3
....uggacgNagaacugauaagggc.....................	22	1	Mm3
....uggacggagaacAgauaagggc.....................	17	1	Mm3
....uggacggaUaacugauaagggc.....................	47	1	Mm3
....uggacgUagaacugauaagggc.....................	53	1	Mm3
....uggacggagaacugauaaggUc.....................	74	1	Mm3
....Nggacggagaacugauaagggc.....................	148	1	Mm3
....uggacgCagaacugauaagggc.....................	17	1	Mm3
....uggacAgagaacugauaagggc.....................	50	1	Mm3
....uggacggagaCcugauaagggc.....................	247	1	Mm3
....uggGcggagaacugauaagggc.....................	58	1	Mm3
....uggacggagaacugaCaagggc.....................	20	1	Mm3
....uggacggagaacuUauaagggc.....................	28	1	Mm3
....uggacggagaacugGuaagggc.....................	29	1	Mm3
....uggacggGgaacugauaagggc.....................	76	1	Mm3
....uggacggagUacugauaagggc.....................	126	1	Mm3
....uggacggagaacugauUagggc.....................	428	1	Mm3
....uggacggagaacugauaagCgc.....................	22	1	Mm3
....uggacggagaaAugauaagggc.....................	9	1	Mm3
....Cggacggagaacugauaagggc.....................	55	1	Mm3
....uggacggagaacugauaagggU.....................	2911	1	Mm3
....uggacggagaacCgauaagggc.....................	35	1	Mm3
....uggacggagaacGgauaagggc.....................	12	1	Mm3
....uggacggagaacugauaGgggc.....................	2855	1	Mm3
....uggacggCgaacugauaagggc.....................	43	1	Mm3
....uggacggagaacugauaaggAc.....................	60	1	Mm3
....uggacggaAaacugauaagggc.....................	19	1	Mm3
....uggacggagaacugauaaggCc.....................	161	1	Mm3
....uggacggaCaacugauaagggc.....................	13	1	Mm3
....uggacNgagaacugauaagggc.....................	38	1	Mm3
....ugNacggagaacugauaagggc.....................	2	1	Mm3
....uggacggagCacugauaagggc.....................	54	1	Mm3
....uggacggagaacugauaUgggc.....................	986	1	Mm3
....uggacggagaGcugauaagggc.....................	541	1	Mm3
....uggacggagaacugauGagggc.....................	556	1	Mm3
....uggacggagaacugauaCgggc.....................	1119	1	Mm3
....uggacggagaaGugauaagggc.....................	6	1	Mm3
....uggacggUgaacugauaagggc.....................	168	1	Mm3
....uggacggagaacuAauaagggc.....................	60	1	Mm3
....uggacggagaacugaAaagggc.....................	10	1	Mm3
....uggacggagaacugauaagUgc.....................	69	1	Mm3
....uggUcggagaacugauaagggc.....................	42	1	Mm3

```
                                  Star                                  Mature
cauuugugacaugagaaguaaguuugucucguguccucuucacuuuuccauccuguGuguacucgagaacuggacggagaacugauaagggcauagggcagaccacaucaaca
......................................................................uggacggagaacugauCagggc.....................    629      1   Mm3
......................................................................uggacggagaacugauaagggA.....................    8184     1   Mm3
......................................................................uggacggagaacugauaagAgc.....................    49       1   Mm3
......................................................................uggaUggagaacugauaagggc.....................    74       1   Mm3
......................................................................uggacggagaacugaGaagggc.....................    2        1   Mm3
......................................................................uggacggagGacugauaagggc.....................    464      1   Mm3
......................................................................uggacggagaacugauaagggc.....................    293785   0   Mm3
......................................................................uUgacggagaacugauaagggc.....................    58       1   Mm3
......................................................................uggacggagaacugUuaagggc.....................    56       1   Mm3
......................................................................uggacggagaacugauaaAggc.....................    31       1   Mm3
......................................................................uggacgAagaacugauaagggc.....................    36       1   Mm3
......................................................................uggaGggagaacugauaagggc.....................    14       1   Mm3
......................................................................uggacggagaaUugauaagggc.....................    74       1   Mm3
......................................................................uCgacggagaacugauaagggc.....................    32       1   Mm3
......................................................................uggacCgagaacugauaagggc.....................    7        1   Mm3
......................................................................ugAacggagaacugauaagggc.....................    81       1   Mm3
......................................................................uggaAggagaacugauaagggc.....................    29       1   Mm3
......................................................................uAgacggagaacugauaagggc.....................    111      1   Mm3
......................................................................uNgacggagaacugauaagggc.....................    3        1   Mm3
......................................................................uggacUgagaacugauaagggc.....................    32       1   Mm3
......................................................................uggacggagaacuCauaagggc.....................    19       1   Mm3
......................................................................Aggacggagaacugauaagggc.....................    284      1   Mm3
......................................................................uggacggagaacugauaaUggc.....................    50       1   Mm3
......................................................................ugUacggagaacugauaagggc.....................    39       1   Mm3
......................................................................uggacggagaacugauaagggG.....................    144      1   Mm3
......................................................................uggacgUagaacugauaagggca....................    3        1   Mm3
......................................................................uggacUgagaacugauaagggca....................    3        1   Mm3
......................................................................uAgacggagaacugauaagggca....................    9        1   Mm3
......................................................................uggacgAagaacugauaagggca....................    2        1   Mm3
......................................................................uggacggagaacugauaagggUa....................    75       1   Mm3
......................................................................uggacggagaacugaCaagggca....................    6        1   Mm3
......................................................................uggacAgagaacugauaagggca....................    5        1   Mm3
......................................................................uCgacggagaacugauaagggca....................    2        1   Mm3
......................................................................uggacggaCaacugauaagggca....................    1        1   Mm3
......................................................................Nggacggagaacugauaagggca....................    10       1   Mm3
......................................................................ugAacggagaacugauaagggca....................    5        1   Mm3
......................................................................uggacggagaacugGuaagggca....................    4        1   Mm3
......................................................................uggacggagaacuUauaagggca....................    2        1   Mm3
......................................................................uggacggagaacugauCagggca....................    79       1   Mm3
......................................................................uggacggagaacugauaaggCca....................    26       1   Mm3
......................................................................uggacggagaacuAauaagggca....................    5        1   Mm3
......................................................................uggacggagaacugauaaUggca....................    4        1   Mm3
......................................................................uggacCgagaacugauaagggca....................    2        1   Mm3
......................................................................uggacggagaacugauUagggca....................    35       1   Mm3
......................................................................uggaUggagaacugauaagggca....................    6        1   Mm3
......................................................................uggacggCgaacugauaagggca....................    4        1   Mm3
......................................................................uggacggagaacugauaagggAa....................    420      1   Mm3
......................................................................uggacggagaacugauaagggca....................    26505    0   Mm3
......................................................................uggacggGgaacugauaagggca....................    4        1   Mm3
......................................................................uggacggagaacugauaaCggca....................    1        1   Mm3
......................................................................uggacggagaacugauGagggca....................    54       1   Mm3
......................................................................uggacggUgaacugauaagggca....................    11       1   Mm3
......................................................................uggacggagaacugauaagUgca....................    5        1   Mm3
......................................................................uggacggagaacugauaagAgca....................    10       1   Mm3
......................................................................uggCcggagaacugauaagggca....................    2        1   Mm3
......................................................................uggacggaUaacugauaagggca....................    4        1   Mm3
......................................................................uggacNgagaacugauaagggca....................    4        1   Mm3
......................................................................uggGcggagaacugauaagggca....................    4        1   Mm3
......................................................................uggacggagaUcugauaagggca....................    2237     1   Mm3
......................................................................ugCacggagaacugauaagggca....................    2        1   Mm3
......................................................................uggaGggagaacugauaagggca....................    1        1   Mm3
......................................................................ugUacggagaacugauaagggca....................    3        1   Mm3
......................................................................uggacggagaacugUuaagggca....................    5        1   Mm3
......................................................................Aggacggagaacugauaagggca....................    14       1   Mm3
......................................................................uggacgNagaacugauaagggca....................    3        1   Mm3
......................................................................uggacggagaacugauaagggcC....................    175      1   Mm3
......................................................................Cggacggagaacugauaagggca....................    4        1   Mm3
......................................................................uggacggagaacugauaCgggca....................    92       1   Mm3
......................................................................uggacggagaacugaAaagggca....................    1        1   Mm3
......................................................................uggacggagaacugauaUgggca....................    78       1   Mm3
```

Star **Mature**

```
cauuugugacaugagaaguaaguuugucucguguccucuucacuuuuccauccugucuguacucgagaacuggacggagaacugauaagggcauagggcagaccacaucaaca
......................................................................uggacggagaacugauaagggcG....................  434   1  Mm3
......................................................................uggacggagaCcugauaagggca....................  22    1  Mm3
......................................................................uggacggagaaUugauaagggca....................  12    1  Mm3
......................................................................uggacggagGacugauaagggca....................  29    1  Mm3
......................................................................uggacggagaacugauaaggUca....................  7     1  Mm3
......................................................................uggacggagaacugauaagCgca....................  2     1  Mm3
......................................................................uggacggagaGcugauaagggca....................  44    1  Mm3
......................................................................uggacggagUacugauaagggca....................  8     1  Mm3
......................................................................uggaAggagaacugauaagggca....................  2     1  Mm3
......................................................................uggacggagaacugauaaggAca....................  4     1  Mm3
......................................................................uggacggagaacAgauaagggca....................  2     1  Mm3
......................................................................uUgacggagaacugauaagggca....................  6     1  Mm3
......................................................................uggacggagCacugauaagggca....................  8     1  Mm3
......................................................................uggacggagaacugauaGgggca....................  185   1  Mm3
......................................................................uggUcggagaacugauaagggca....................  5     1  Mm3
......................................................................uggacggagaacugauaagggGa....................  3     1  Mm3
......................................................................uggacggagaacugauCagggcau...................  7     1  Mm3
......................................................................uggacNgagaacugauaagggcau...................  1     1  Mm3
......................................................................uCgacggagaacugauaagggcau...................  1     1  Mm3
......................................................................uggacggagaacugauaagggcaG...................  22    1  Mm3
......................................................................Aggacggagaacugauaagggcau...................  1     1  Mm3
......................................................................uggacggagaacugauaagggcaA...................  59    1  Mm3
......................................................................Nggacggagaacugauaagggcau...................  1     1  Mm3
......................................................................uggacggagaacugauaagggcGu...................  19    1  Mm3
......................................................................uggacggaUaacugauaagggcau...................  1     1  Mm3
......................................................................uggacggagaCcugauaagggcau...................  1     1  Mm3
......................................................................uggacggagaacugauaagggUau...................  167   1  Mm3
......................................................................uggacggagaacuAauaagggcau...................  1     1  Mm3
......................................................................uggCcggagaacugauaagggcau...................  1     1  Mm3
......................................................................uggacggagCacugauaagggcau...................  2     1  Mm3
......................................................................uggacggagGacugauaagggcau...................  1     1  Mm3
......................................................................uggacggagaacugauaagggcau...................  2304  0  Mm3
......................................................................uggUcggagaacugauaagggcau...................  1     1  Mm3
......................................................................uggacggagaacugauaaggUcau...................  2     1  Mm3
......................................................................uggacggagaacugauaCgggcau...................  13    1  Mm3
......................................................................uggacggagaGcugauaagggcau...................  5     1  Mm3
......................................................................uggacggUgaacugauaagggcau...................  2     1  Mm3
......................................................................uAgacggagaacugauaagggcau...................  1     1  Mm3
......................................................................uggacggagaacugauaagggAau...................  323   1  Mm3
......................................................................uggacggagaUcugauaagggcau...................  247   1  Mm3
......................................................................uggacggagaacugauaagUgcau...................  1     1  Mm3
......................................................................uggacggagaacugauaGgggcau...................  24    1  Mm3
......................................................................uggaUggagaacugauaagggcau...................  1     1  Mm3
......................................................................uggacggagaacCgauaagggcau...................  1     1  Mm3
......................................................................uggacggagaacugauaagggcCu...................  24    1  Mm3
......................................................................uggacggagaacugauaagggcaC...................  20    1  Mm3
......................................................................uggacggagaacugauaUgggcau...................  6     1  Mm3
......................................................................uggacggagaacugauUagggcau...................  2     1  Mm3
......................................................................uggacggagaacugauaagggcaua..................  2     0  Mm3
......................................................................uggacggagaacugauaagggcauC..................  4     1  Mm3
......................................................................uggacggagaacugauaagggcauU..................  360   1  Mm3
......................................................................uggacggagaUcugauaagggcaua..................  1     1  Mm3
......................................................................uggacggagaacugauaagggcauG..................  1     1  Mm3
......................................................................uggacggagaacugauaagggcUua..................  10    1  Mm3
......................................................................uggacggagaacugauaagggcaAa..................  2     1  Mm3
......................................................................uggacggagaacugauaagggcauaU.................  4     1  Mm3
......................................................................uggacggagaacugauaagggcaAag.................  1     1  Mm3
.......................................................................ggacggagaacugauaaU........................  3     1  Mm3
.......................................................................ggacggagaacugauaaA........................  12    1  Mm3
.......................................................................ggacggagaacugauaag........................  18    0  Mm3
.......................................................................ggacggagaCcugauaagg.......................  1     1  Mm3
.......................................................................ggacggagCacugauaagg.......................  1     1  Mm3
.......................................................................ggacggagaacugauaagC.......................  1     1  Mm3
.......................................................................Ugacggagaacugauaagg.......................  1     1  Mm3
.......................................................................ggacggagaacugauaagg.......................  33    0  Mm3
.......................................................................ggacggagaacugauaagA.......................  16    1  Mm3
.......................................................................ggacggCgaacugauaagg.......................  1     1  Mm3
.......................................................................ggacggagaacugauaaggU......................  1     1  Mm3
.......................................................................ggacggagaGcugauaaggg......................  2     1  Mm3
.......................................................................ggacggagaacuUauaaggg......................  1     1  Mm3
```

Star Mature

```
cauuugugacaugagaaguaaguuugucucguguccucuucacuuuuccauccugucuguacucgagaacuggacggagaacugauaagggcauagggcagaccacaucaaca
.......................................................................ggacggagCacugauaaggg......................  2    1  Mm3
.......................................................................ggacggagaacugauaaggg......................  61   0  Mm3
.......................................................................Ugacggagaacugauaaggg......................  2    1  Mm3
.......................................................................ggacggagaacugauaaggA......................  14   1  Mm3
.......................................................................ggacgCagaacugauaaggg......................  1    1  Mm3
.......................................................................ggaAggagaacugauaagggc.....................  1    1  Mm3
.......................................................................ggacggagaaAugauaagggc.....................  1    1  Mm3
.......................................................................Ngacggagaacugauaagggc.....................  1    1  Mm3
.......................................................................ggacggagaacugauaUgggc.....................  1    1  Mm3
.......................................................................ggacggagaacAgauaagggc.....................  1    1  Mm3
.......................................................................ggacggagaaUugauaagggc.....................  1    1  Mm3
.......................................................................ggCcggagaacugauaagggc.....................  1    1  Mm3
.......................................................................ggacggagGacugauaagggc.....................  4    1  Mm3
.......................................................................ggacggagaacugauaagggG.....................  1    1  Mm3
.......................................................................ggacggagaacugauaagggc.....................  621  0  Mm3
.......................................................................Ugacggagaacugauaagggc.....................  12   1  Mm3
.......................................................................ggacggagaacugauaaUggc.....................  1    1  Mm3
.......................................................................ggacggUgaacugauaagggc.....................  1    1  Mm3
.......................................................................ggacggagaacugauaagAgc.....................  2    1  Mm3
.......................................................................ggacggagaacugauaCgggc.....................  2    1  Mm3
.......................................................................ggacCgagaacugauaagggc.....................  1    1  Mm3
.......................................................................ggacggagaacugaAaagggc.....................  1    1  Mm3
.......................................................................ggacggagaacugauaaggAc.....................  2    1  Mm3
.......................................................................ggacggaAaacugauaagggc.....................  1    1  Mm3
.......................................................................ggacggagaacugauaaggUc.....................  1    1  Mm3
.......................................................................ggacggagCacugauaagggc.....................  6    1  Mm3
.......................................................................ggacggagaacugauaGgggc.....................  5    1  Mm3
.......................................................................ggacggagaacugauaagUgc.....................  1    1  Mm3
.......................................................................ggacggagaacugauaagggU.....................  9    1  Mm3
.......................................................................ggacggagaacugauGagggc.....................  1    1  Mm3
.......................................................................ggacggagaCcugauaagggc.....................  1    1  Mm3
.......................................................................ggacggagaacugauaaAggc.....................  1    1  Mm3
.......................................................................ggacggagaaGugauaagggc.....................  1    1  Mm3
.......................................................................ggacggagaacugauCagggc.....................  4    1  Mm3
.......................................................................ggacggagaacugauaagggA.....................  13   1  Mm3
.......................................................................gUacggagaacugauaagggc.....................  2    1  Mm3
.......................................................................ggacggaUaacugauaagggc.....................  1    1  Mm3
.......................................................................ggacggagaacugauUagggca....................  1    1  Mm3
.......................................................................ggacggagaacCgauaagggca....................  1    1  Mm3
.......................................................................ggacggagaUcugauaagggca....................  7    1  Mm3
.......................................................................ggacggagaacugauaagggAa....................  1    1  Mm3
.......................................................................ggGcggagaacugauaagggca....................  1    1  Mm3
.......................................................................ggacggagaacugauaagggca....................  61   0  Mm3
.......................................................................ggacggagaacugauaagggcau...................  8    0  Mm3
.......................................................................ggacggagaacugauaUgggcau...................  1    1  Mm3
.......................................................................ggacggaUaacugauaagggcau...................  1    1  Mm3
.......................................................................ggaUggagaacugauaagggcau...................  1    1  Mm3
.......................................................................ggacggagaacugauaagggcauU..................  1    1  Mm3
........................................................................gacggagaacugauaagggc.....................  3    0  Mm3
.........................................................................acggagaacugauaagggc.....................  2    0  Mm3
.........................................................................acggagaacugauaagggca....................  2    0  Mm3

..................................ccucuucacuuuuUcauccugu.........................................................  2    1  Fm3
..................................ccucuucacuuuuccauccugu.........................................................  5    0  Fm3
...................................cucuucacuuuuccauccugu.........................................................  1    0  Fm3
...................................................................aacuggacggagaacugauaaU........................  1    1  Fm3
...................................................................aacuggacggagaacugauaagU.......................  1    1  Fm3
...................................................................aUcuggacggagaacugauaagggc.....................  4    1  Fm3
....................................................................acuggacggagaacugaua..........................  2    0  Fm3
....................................................................acuggacggagaacugauaag........................  3    0  Fm3
....................................................................Ucuggacggagaacugauaagg.......................  1    1  Fm3
....................................................................acuggacggagaacugauaagg.......................  6    0  Fm3
....................................................................acuggacggagaacugauaagU.......................  6    1  Fm3
....................................................................acuggacggagaacugauaaggA......................  4    1  Fm3
....................................................................Ucuggacggagaacugauaaggg......................  2    1  Fm3
....................................................................acuggacggagaacugauaaggU......................  7    1  Fm3
....................................................................Ucuggacggagaacugauaagggc.....................  1    1  Fm3
....................................................................acuggacggagaacugauaagggc.....................  1    0  Fm3
.....................................................................cuggacggagaacugaua..........................  12   0  Fm3
.....................................................................cuggacggagaacugaAa..........................  5    1  Fm3
```

```
                                  Star                                      Mature
cauuugugacaugagaaguaaguuugucucguguccucuucacuuuuccauccuguCuguacucgagaacuggacggagaacugauaagggcauagggcagaccacaucaaca
.....................................................................cuggacggagaacugauU..........................   1       1       Fm3
.....................................................................cuggacggagaacugauaa.........................   16      0       Fm3
.....................................................................cuggacggagaacugauaG.........................   1       1       Fm3
.....................................................................cuggacggagaacugauaag........................   103     0       Fm3
.....................................................................cuggacggagaacugauaaU........................   11      1       Fm3
.....................................................................cuggacggagaacugauUag........................   1       1       Fm3
.....................................................................Guggacggagaacugauaag........................   1       1       Fm3
.....................................................................cuggacggagaacugauaaA........................   23      1       Fm3
.....................................................................cuggacggagaacugaAaagg.......................   1       1       Fm3
.....................................................................cuggacggagCacugauaagg.......................   1       1       Fm3
.....................................................................cuggacggagGacugauaagg.......................   1       1       Fm3
.....................................................................cuggacggagaacugauGagg.......................   1       1       Fm3
.....................................................................cuggacggagaacugauaagU.......................   9       1       Fm3
.....................................................................cuggacggagaacugauCagg.......................   2       1       Fm3
.....................................................................cuggacggagaacugaCaagg.......................   3       1       Fm3
.....................................................................cuggacggagaacugauaagA.......................   70      1       Fm3
.....................................................................cuggacggagaacugauaagg.......................   238     0       Fm3
.....................................................................cuggacggagaacugauaaAg.......................   1       1       Fm3
.....................................................................cuggacggagaacugauUagg.......................   1       1       Fm3
.....................................................................cuggacggagaacugauGaggg......................   3       1       Fm3
.....................................................................cuggacggagaaAugauaaggg......................   1       1       Fm3
.....................................................................cuggacggagaacugaGaaggg......................   1       1       Fm3
.....................................................................Nuggacggagaacugauaaggg......................   1       1       Fm3
.....................................................................cuggacggagaacugauaaggU......................   46      1       Fm3
.....................................................................cuggacggagCacugauaaggg......................   2       1       Fm3
.....................................................................cuggacggagaacugauaaggC......................   3       1       Fm3
.....................................................................cuggacggagaacugauCaggg......................   1       1       Fm3
.....................................................................cuggacggagGacugauaaggg......................   2       1       Fm3
.....................................................................cuggacggagaacugauaaggg......................   284     0       Fm3
.....................................................................cuggacggagaacugauaaggA......................   67      1       Fm3
.....................................................................cuggacggagaacugauaagggU.....................   26      1       Fm3
.....................................................................cuggacggagaacugaAaagggc.....................   1       1       Fm3
.....................................................................cuggacggagaacugauaagggA.....................   64      1       Fm3
.....................................................................cuggacggagaacugauaGgggc.....................   2       1       Fm3
.....................................................................cuggacggagaacugauaagggc.....................   212     0       Fm3
.....................................................................cuggUcggagaacugauaagggc.....................   1       1       Fm3
.....................................................................cuggacggGgaacugauaagggc.....................   1       1       Fm3
.....................................................................cuggacggagaacugaCaagggc.....................   1       1       Fm3
.....................................................................cuggacggagaacugauaagggAa....................   1       1       Fm3
.....................................................................cuggacggagaaUugauaagggca....................   1       1       Fm3
.....................................................................cuggacggagaUcugauaagggca....................   3       1       Fm3
.....................................................................cuggacggagaacugauaUgggca....................   1       1       Fm3
.....................................................................cuggacggagaacugauaagggca....................   51      0       Fm3
.....................................................................cuggacggagaacugauCagggca....................   1       1       Fm3
......................................................................Cggacggagaacugauaa.........................   1       1       Fm3
......................................................................uggacggagaacuCauaa.........................   1       1       Fm3
......................................................................uggacggagaaUugauaa.........................   1       1       Fm3
......................................................................uggacggagCacugauaa.........................   2       1       Fm3
......................................................................uggUcggagaacugauaa.........................   1       1       Fm3
......................................................................uggacggagaacuAauaa.........................   6       1       Fm3
......................................................................uggacggaAaacugauaa.........................   2       1       Fm3
......................................................................uggacggagaacugauaC.........................   58      1       Fm3
......................................................................uggacggaUaacugauaa.........................   3       1       Fm3
......................................................................uggacggagaacugauaU.........................   591     1       Fm3
......................................................................uggacgAagaacugauaa.........................   3       1       Fm3
......................................................................uggacggagaacugauCa.........................   41      1       Fm3
......................................................................uggacggagaGcugauaa.........................   27      1       Fm3
......................................................................uggacggagaacugauaa.........................   18785   0       Fm3
......................................................................ugCacggagaacugauaa.........................   1       1       Fm3
......................................................................uUgacggagaacugauaa.........................   4       1       Fm3
......................................................................uggacggagGacugauaa.........................   15      1       Fm3
......................................................................uggacggagaacugauUa.........................   54      1       Fm3
......................................................................Aggacggagaacugauaa.........................   2       1       Fm3
......................................................................uggacggagaacugUuaa.........................   5       1       Fm3
......................................................................uAgacggagaacugauaa.........................   14      1       Fm3
......................................................................uggacggagaacugauGa.........................   23      1       Fm3
......................................................................ugUacggagaacugauaa.........................   9       1       Fm3
......................................................................uggacUgagaacugauaa.........................   8       1       Fm3
......................................................................uggacggagaCcugauaa.........................   13      1       Fm3
......................................................................uggacggagaacugaGaa.........................   5       1       Fm3
```

Star **Mature**

cauuugugacaugagaaguaaguuugucucguguccucuucacuuuuccauccugucuguacucgagaacuggacggagaacugauaagggcauagggcagaccacaucaaca			
....uggaGggagaacugauaa.........................	1	1	Fm3
....uggacgCagaacugauaa.........................	1	1	Fm3
....uggacggagaacugGuaa.........................	1	1	Fm3
....uggGcggagaacugauaa.........................	2	1	Fm3
....uggacgUagaacugauaa.........................	7	1	Fm3
....uggacggCgaacugauaa.........................	1	1	Fm3
....uggacggaCaacugauaa.........................	2	1	Fm3
....uggacggagaacGgauaa.........................	1	1	Fm3
....uggacggagaacCgauaa.........................	3	1	Fm3
....ugAacggagaacugauaa.........................	5	1	Fm3
....Nggacggagaacugauaa.........................	6	1	Fm3
....uCgacggagaacugauaa.........................	3	1	Fm3
....uggacgNagaacugauaa.........................	1	1	Fm3
....uggCcggagaacugauaa.........................	6	1	Fm3
....uggacAgagaacugauaa.........................	2	1	Fm3
....uggacggagaacugaAaa.........................	1643	1	Fm3
....uggacggagaacugauaG.........................	105	1	Fm3
....uggaUggagaacugauaa.........................	6	1	Fm3
....uggacggGgaacugauaa.........................	3	1	Fm3
....uggacggUgaacugauaa.........................	7	1	Fm3
....uggacggagaacCgauaag........................	9	1	Fm3
....Cggacggagaacugauaag........................	9	1	Fm3
....uggacggaUaacugauaag........................	19	1	Fm3
....uUgacggagaacugauaag........................	37	1	Fm3
....uggacggagCacugauaag........................	5	1	Fm3
....uggacggagaacugauaUg........................	256	1	Fm3
....uAgacggagaacugauaag........................	53	1	Fm3
....uggacggagaaAugauaag........................	2	1	Fm3
....uggacggagaacuAauaag........................	10	1	Fm3
....uggaUggagaacugauaag........................	21	1	Fm3
....uggacggagaacugauaGg........................	245	1	Fm3
....uggacggagaacugauaag........................	75493	0	Fm3
....uggacggagaacugauCag........................	167	1	Fm3
....ugNacggagaacugauaag........................	1	1	Fm3
....uggacggCgaacugauaag........................	6	1	Fm3
....uggacggaCaacugauaag........................	2	1	Fm3
....uggacggagaacugauaaA........................	48211	1	Fm3
....uggGcggagaacugauaag........................	17	1	Fm3
....uggacgAagaacugauaag........................	6	1	Fm3
....uggacgNagaacugauaag........................	3	1	Fm3
....uggacggagaacugCuaag........................	4	1	Fm3
....uggacggagaacugaGaag........................	2	1	Fm3
....uggacgUagaacugauaag........................	35	1	Fm3
....uggacggagaacGgauaag........................	1	1	Fm3
....uggacggagaacuCauaag........................	7	1	Fm3
....uggacggagaacugUuaag........................	10	1	Fm3
....ugCacggagaacugauaag........................	8	1	Fm3
....uggacggagaCcugauaag........................	116	1	Fm3
....ugAacggagaacugauaag........................	17	1	Fm3
....Aggacggagaacugauaag........................	41	1	Fm3
....uggacggagaaUugauaag........................	23	1	Fm3
....uCgacggagaacugauaag........................	6	1	Fm3
....uggacggagaacugGuaag........................	9	1	Fm3
....uNgacggagaacugauaag........................	2	1	Fm3
....uggacUgagaacugauaag........................	28	1	Fm3
....uggacggGgaacugauaag........................	9	1	Fm3
....Gggacggagaacugauaag........................	4	1	Fm3
....uggacggagaacuUauaag........................	8	1	Fm3
....uggacggagUacugauaag........................	18	1	Fm3
....uggacggagaacugauGag........................	102	1	Fm3
....uggacggagaacAgauaag........................	7	1	Fm3
....uggacAgagaacugauaag........................	17	1	Fm3
....Nggacggagaacugauaag........................	30	1	Fm3
....uggacggagaacugauaCg........................	298	1	Fm3
....uggacggagaacugaCaag........................	8	1	Fm3
....uggacggagaacugaAaag........................	60	1	Fm3
....uggacggagaGcugauaag........................	243	1	Fm3
....uggacggagaacugauaaU........................	15180	1	Fm3
....uggacggUgaacugauaag........................	17	1	Fm3
....uggacggagaaGugauaag........................	3	1	Fm3

```
                                                  Star                                Mature
cauuugugacaugagaaguaaguuugucucguguccucuucacuuuuccauccugucuguacucgagaacuggacggagaacugauaagggcauagggcagaccacaucaaca
......................................................................uggaGggagaacugauaag........................ 7      1 Fm3
......................................................................uggacggagaacugauaaC........................ 52     1 Fm3
......................................................................uggacCgagaacugauaag........................ 2      1 Fm3
......................................................................uggacggagaacugauUag........................ 116    1 Fm3
......................................................................uggacNgagaacugauaag........................ 7      1 Fm3
......................................................................uggacgCagaacugauaag........................ 7      1 Fm3
......................................................................uggCcggagaacugauaag........................ 11     1 Fm3
......................................................................uggacggaAaacugauaag........................ 4      1 Fm3
......................................................................uggaAggagaacugauaag........................ 11     1 Fm3
......................................................................ugUacggagaacugauaag........................ 27     1 Fm3
......................................................................uggUcggagaacugauaag........................ 14     1 Fm3
......................................................................uggacggagGacugauaag........................ 72     1 Fm3
......................................................................uggacggagaacuAauaagg....................... 33     1 Fm3
......................................................................uggUcggagaacugauaagg....................... 25     1 Fm3
......................................................................uggacggagaaUugauaagg....................... 31     1 Fm3
......................................................................uggacggagUacugauaagg....................... 32     1 Fm3
......................................................................uggacggagaacugauaGgg....................... 663    1 Fm3
......................................................................uggacCgagaacugauaagg....................... 6      1 Fm3
......................................................................uggacggagGacugauaagg....................... 235    1 Fm3
......................................................................uggacggagaacugauaagU....................... 5388   1 Fm3
......................................................................uggacggagaacugaCaagg....................... 19     1 Fm3
......................................................................ugCacggagaacugauaagg....................... 13     1 Fm3
......................................................................uggGcggagaacugauaagg....................... 36     1 Fm3
......................................................................uggacggagaaGugauaagg....................... 4      1 Fm3
......................................................................uggacggagaacugauaagg....................... 154018 0 Fm3
......................................................................uggacAgagaacugauaagg....................... 37     1 Fm3
......................................................................uggacggagaGcugauaagg....................... 1812   1 Fm3
......................................................................uggaAggagaacugauaagg....................... 20     1 Fm3
......................................................................uggaUggagaacugauaagg....................... 57     1 Fm3
......................................................................Aggacggagaacugauaagg....................... 638    1 Fm3
......................................................................uggacggagCacugauaagg....................... 14     1 Fm3
......................................................................uggacggagaacugauaaCg....................... 5      1 Fm3
......................................................................uggacggagaaAugauaagg....................... 10     1 Fm3
......................................................................uggacggagaacugGuaagg....................... 15     1 Fm3
......................................................................Nggacggagaacugauaagg....................... 83     1 Fm3
......................................................................uggacgAagaacugauaagg....................... 12     1 Fm3
......................................................................uggacggagaacugUuaagg....................... 33     1 Fm3
......................................................................uCgacggagaacugauaagg....................... 18     1 Fm3
......................................................................uggacggUgaacugauaagg....................... 37     1 Fm3
......................................................................Cggacggagaacugauaagg....................... 26     1 Fm3
......................................................................uggacggCgaacugauaagg....................... 9      1 Fm3
......................................................................uggacggaCaacugauaagg....................... 11     1 Fm3
......................................................................ugUacggagaacugauaagg....................... 42     1 Fm3
......................................................................uggacggagaacAgauaagg....................... 4      1 Fm3
......................................................................uggacggagaacugauaaUg....................... 45     1 Fm3
......................................................................uggacggagaacuCauaagg....................... 9      1 Fm3
......................................................................Gggacggagaacugauaagg....................... 16     1 Fm3
......................................................................uggacgUagaacugauaagg....................... 46     1 Fm3
......................................................................uggacggagaacugaAaagg....................... 7      1 Fm3
......................................................................uggaGggagaacugauaagg....................... 8      1 Fm3
......................................................................uggacggaAaacugauaagg....................... 10     1 Fm3
......................................................................uggacggaUaacugauaagg....................... 32     1 Fm3
......................................................................uggacggagaacugauaagC....................... 22     1 Fm3
......................................................................uggacggagaacugauaaAg....................... 936    1 Fm3
......................................................................uggacggagaacugauGagg....................... 266    1 Fm3
......................................................................uggacggagaacugauaCgg....................... 648    1 Fm3
......................................................................uNgacggagaacugauaagg....................... 1      1 Fm3
......................................................................uggacggagaCcugauaagg....................... 1074   1 Fm3
......................................................................ugNacggagaacugauaagg....................... 1      1 Fm3
......................................................................uggacggagaacugauCagg....................... 354    1 Fm3
......................................................................uggacggGgaacugauaagg....................... 22     1 Fm3
......................................................................uggacggagaacugauaagA....................... 43430  1 Fm3
......................................................................uggacggagaacGgauaagg....................... 7      1 Fm3
......................................................................uggacggagaacuUauaagg....................... 17     1 Fm3
......................................................................uggCcggagaacugauaagg....................... 34     1 Fm3
......................................................................uggacggagaacugCuaagg....................... 10     1 Fm3
......................................................................uggacgNagaacugauaagg....................... 9      1 Fm3
......................................................................uggacgCagaacugauaagg....................... 11     1 Fm3
......................................................................uggacggagaacugauUagg....................... 249    1 Fm3
......................................................................uggacNgagaacugauaagg....................... 17     1 Fm3
```

Star Mature

cauuugugacaugagaaguaaguuugucucguguccucuucacuuuuccauccugucuguacucgagaacuggacggagaacugauaagggcauagggcagaccacaucaaca

....ugAacggagaacugauaagg.......................	36	1	Fm3
....uUgacggagaacugauaagg.......................	57	1	Fm3
....uggacggagaacCgauaagg.......................	19	1	Fm3
....uggacggagaacugauaUgg.......................	476	1	Fm3
....uggacggagaacugaGaagg.......................	6	1	Fm3
....uAgacggagaacugauaagg.......................	79	1	Fm3
....uggacUgagaacugauaagg.......................	50	1	Fm3
....uggacggagaacugauUaggg......................	327	1	Fm3
....uggacggagaacugauaagAg......................	189	1	Fm3
....ugUacggagaacugauaaggg......................	57	1	Fm3
....uggacggagaacugauaaggC......................	78	1	Fm3
....uggacgAagaacugauaaggg......................	13	1	Fm3
....uggacggagaacugUuaaggg......................	48	1	Fm3
....uggacggagaacugauaGggg......................	1166	1	Fm3
....uggaGggagaacugauaaggg......................	5	1	Fm3
....ugAacggagaacugauaaggg......................	49	1	Fm3
....uggacggaAaacugauaaggg......................	13	1	Fm3
....uggacggagaacugauaaCgg......................	5	1	Fm3
....uggacggagaacugaCaaggg......................	20	1	Fm3
....uggacggagaaUugauaaggg......................	50	1	Fm3
....uggacggagaacugaGaaggg......................	4	1	Fm3
....uCgacggagaacugauaaggg......................	18	1	Fm3
....uggacggagaacuAauaaggg......................	52	1	Fm3
....uAgacggagaacugauaaggg......................	98	1	Fm3
....uggacggCgaacugauaaggg......................	14	1	Fm3
....uggacggagaaGugauaaggg......................	6	1	Fm3
....uggacNgagaacugauaaggg......................	30	1	Fm3
....uggaAggagaacugauaaggg......................	20	1	Fm3
....uggacggagaacugauaaAgg......................	22	1	Fm3
....uggGcggagaacugauaaggg......................	44	1	Fm3
....uggCcggagaacugauaaggg......................	29	1	Fm3
....uggacggagCacugauaaggg......................	17	1	Fm3
....Cggacggagaacugauaaggg......................	31	1	Fm3
....uggacggagaacugGuaaggg......................	33	1	Fm3
....uggUcggagaacugauaaggg......................	33	1	Fm3
....uggacggagaacugauaaggA......................	98094	1	Fm3
....uggacggagaacugauGaggg......................	294	1	Fm3
....uggacAgagaacugauaaggg......................	34	1	Fm3
....uggacgCagaacugauaaggg......................	12	1	Fm3
....uggacggGgaacugauaaggg......................	44	1	Fm3
....uggacggagGacugauaaggg......................	195	1	Fm3
....uggacggaUaacugauaaggg......................	41	1	Fm3
....uggacggagaGcugauaaggg......................	1292	1	Fm3
....Gggacggagaacugauaaggg......................	12	1	Fm3
....uggacggagaacugauaCggg......................	780	1	Fm3
....uggacggagaacugauCaggg......................	465	1	Fm3
....Nggacggagaacugauaaggg......................	111	1	Fm3
....uggacggaCaacugauaaggg......................	8	1	Fm3
....uggacggagUacugauaaggg......................	41	1	Fm3
....uggacggagaacugauaagCg......................	15	1	Fm3
....uggacggagaaAugauaaggg......................	6	1	Fm3
....uggacggagaCcugauaaggg......................	674	1	Fm3
....uggacggagaacugauaUggg......................	709	1	Fm3
....uggacCgagaacugauaaggg......................	7	1	Fm3
....uggacgNagaacugauaaggg......................	10	1	Fm3
....uggacggagaacAgauaaggg......................	10	1	Fm3
....uggacggagaacugauaaggU......................	7452	1	Fm3
....Aggacggagaacugauaaggg......................	389	1	Fm3
....uggacggagaacuUauaaggg......................	25	1	Fm3
....uUgacggagaacugauaaggg......................	58	1	Fm3
....uggacUgagaacugauaaggg......................	73	1	Fm3
....uggacggagaacGgauaaggg......................	11	1	Fm3
....uggacggagaacugauaaUgg......................	103	1	Fm3
....uggacggUgaacugauaaggg......................	90	1	Fm3
....uggacgUagaacugauaaggg......................	63	1	Fm3
....uggacggagaacugauaaggg......................	200595	0	Fm3
....uNgacggagaacugauaaggg......................	3	1	Fm3
....uggacggagaacugauaagUg......................	79	1	Fm3
....ugCacggagaacugauaaggg......................	14	1	Fm3
....uggaUggagaacugauaaggg......................	66	1	Fm3

```
                                                                  Star                     Mature
cauuugugacaugagaaguaaguuugucucguguccucuucacuuuuccauccuguc uguacucgagaacuggacggagaacugauaagggcauagggcagaccacaucaaca
......................................................................uggacggagaacuCauaaggg......................	14	1	Fm3
......................................................................uggacggagaacugCuaaggg......................	11	1	Fm3
......................................................................ugNacggagaacugauaaggg......................	1	1	Fm3
......................................................................uggacggagaacugaAaaggg......................	13	1	Fm3
......................................................................uggacggagaacCgauaaggg......................	22	1	Fm3
......................................................................uggacggagaacuCauaagggc.....................	58	1	Fm3
......................................................................uggacggagCacugauaagggc.....................	55	1	Fm3
......................................................................uggaGggagaacugauaagggc.....................	32	1	Fm3
......................................................................uggacNgagaacugauaagggc.....................	104	1	Fm3
......................................................................uggacggagGacugauaagggc.....................	581	1	Fm3
......................................................................uggacggagaacugaGaagggc.....................	19	1	Fm3
......................................................................uggacggagaacugauaagggc.....................	790111	0	Fm3
......................................................................uggCcggagaacugauaagggc.....................	161	1	Fm3
......................................................................uggacggaUaacugauaagggc.....................	182	1	Fm3
......................................................................Gggacggagaacugauaagggc.....................	40	1	Fm3
......................................................................uggacggagaacugauNagggc.....................	3	1	Fm3
......................................................................uggacggagaacugauaaAggc.....................	51	1	Fm3
......................................................................uggacggagaacugauaaUggc.....................	308	1	Fm3
......................................................................uggacggagaCcugauaagggc.....................	696	1	Fm3
......................................................................Cggacggagaacugauaagggc.....................	141	1	Fm3
......................................................................uggaUggagaacugauaagggc.....................	278	1	Fm3
......................................................................uggacggagaacuUauaagggc.....................	83	1	Fm3
......................................................................uggacggagaacugauUagggc.....................	911	1	Fm3
......................................................................uggacggagaacugauaGgggc.....................	5830	1	Fm3
......................................................................uggacggagaacugauaaggAc.....................	157	1	Fm3
......................................................................uggacggagaacugauaUgggc.....................	2622	1	Fm3
......................................................................uggacggagaaGugauaagggc.....................	15	1	Fm3
......................................................................uggacggagaacCgauaagggc.....................	90	1	Fm3
......................................................................ugUacggagaacugauaagggc.....................	244	1	Fm3
......................................................................uggacggagaacugauaagUgc.....................	281	1	Fm3
......................................................................uggGcggagaacugauaagggc.....................	198	1	Fm3
......................................................................ugNacggagaacugauaagggc.....................	1	1	Fm3
......................................................................ugCacggagaacugauaagggc.....................	61	1	Fm3
......................................................................uggacggUgaacugauaagggc.....................	214	1	Fm3
......................................................................Aggacggagaacugauaagggc.....................	237	1	Fm3
......................................................................uggaAggagaacugauaagggc.....................	91	1	Fm3
......................................................................uggacggagaacugauaagAgc.....................	104	1	Fm3
......................................................................uggacggaAaacugauaagggc.....................	51	1	Fm3
......................................................................uggacggagaacugauaCgggc.....................	3025	1	Fm3
......................................................................uggacggagaacugauaaNggc.....................	1	1	Fm3
......................................................................uggacggagaGcugauaagggc.....................	1460	1	Fm3
......................................................................uAgacggagaacugauaagggc.....................	387	1	Fm3
......................................................................uggacggagaacAgauaagggc.....................	51	1	Fm3
......................................................................uggacggagaacugGuaagggc.....................	95	1	Fm3
......................................................................uNgacggagaacugauaagggc.....................	10	1	Fm3
......................................................................uggacggagaaAugauaagggc.....................	34	1	Fm3
......................................................................ugAacggagaacugauaagggc.....................	192	1	Fm3
......................................................................uUgacggagaacugauaagggc.....................	256	1	Fm3
......................................................................uggacggagaacugaAaagggc.....................	29	1	Fm3
......................................................................uggacggagaacugauaagCgc.....................	49	1	Fm3
......................................................................uggacggagaaUugauaagggc.....................	189	1	Fm3
......................................................................uggacggagaacugauaagggU.....................	12581	1	Fm3
......................................................................uCgacggagaacugauaagggc.....................	85	1	Fm3
......................................................................uggacgAagaacugauaagggc.....................	78	1	Fm3
......................................................................uggacggaCaacugauaagggc.....................	45	1	Fm3
......................................................................uggacggagaacugCuaagggc.....................	28	1	Fm3
......................................................................uggacggagaacugauaagggG.....................	97	1	Fm3
......................................................................uggacggCgaacugauaagggc.....................	42	1	Fm3
......................................................................uggacgUagaacugauaagggc.....................	263	1	Fm3
......................................................................uggacggagaacuAauaagggc.....................	167	1	Fm3
......................................................................uggacggagaacugauaaCggc.....................	38	1	Fm3
......................................................................uggacCgagaacugauaagggc.....................	40	1	Fm3
......................................................................uggacggagaacugauaagggA.....................	100006	1	Fm3
......................................................................uggacggagaacugauCagggc.....................	1554	1	Fm3
......................................................................uggacgNagaacugauaagggc.....................	41	1	Fm3
......................................................................uggacggagaacGgauaagggc.....................	25	1	Fm3
......................................................................uggacggagUacugauaagggc.....................	123	1	Fm3
......................................................................uggacggagaacugauaaggCc.....................	71	1	Fm3
......................................................................uggacUgagaacugauaagggc.....................	245	1	Fm3
......................................................................uggacggagaacugUuaagggc.....................	135	1	Fm3
```

Star Mature

```
cauuugugacaugagaaguaaguuugucucguguccucuucacuuuuccauccuguCuguacucgagaacuggacggagaacugauaagggcauagggcagaccacaucaaca
......................................................................uggacggGgaacugauaagggc.....................  180     1  Fm3
......................................................................uggacggagaacugauGagggc.....................  1829    1  Fm3
......................................................................uggUcggagaacugauaagggc.....................  147     1  Fm3
......................................................................uggacggagaacugauaaggUc.....................  273     1  Fm3
......................................................................uggacAgagaacugauaagggc.....................  155     1  Fm3
......................................................................uggacggagaacugaCaagggc.....................  82      1  Fm3
......................................................................Nggacggagaacugauaagggc.....................  405     1  Fm3
......................................................................uggacgCagaacugauaagggc.....................  46      1  Fm3
......................................................................uggacgNagaacugauaagggca....................  9       1  Fm3
......................................................................uggacggagaacugauaCgggca....................  617     1  Fm3
......................................................................uggacggagaacugauaagggcC....................  86      1  Fm3
......................................................................uggacggagaacuUauaagggca....................  14      1  Fm3
......................................................................uggacgCagaacugauaagggca....................  11      1  Fm3
......................................................................uggacggagaacugauaUgggca....................  546     1  Fm3
......................................................................uggacggagaacugauaGgggca....................  818     1  Fm3
......................................................................uggacggaCaacugauaagggca....................  7       1  Fm3
......................................................................uggacggagaacugauaagggca....................  165997  0  Fm3
......................................................................uggacggagaacugauaagUgca....................  52      1  Fm3
......................................................................uUgacggagaacugauaagggca....................  63      1  Fm3
......................................................................uggacggagaacugauCagggca....................  330     1  Fm3
......................................................................ugNacggagaacugauaagggca....................  2       1  Fm3
......................................................................uggacggagaacAgauaagggca....................  7       1  Fm3
......................................................................uggacggagaacugaGaagggca....................  6       1  Fm3
......................................................................ugCacggagaacugauaagggca....................  16      1  Fm3
......................................................................uggacggagaacugGuaagggca....................  19      1  Fm3
......................................................................uCgacggagaacugauaagggca....................  17      1  Fm3
......................................................................uggacggCgaacugauaagggca....................  10      1  Fm3
......................................................................uggacggagaacugauaaggUca....................  39      1  Fm3
......................................................................uggacggagUacugauaagggca....................  32      1  Fm3
......................................................................uggacNgagaacugauaagggca....................  17      1  Fm3
......................................................................uggacggagGacugauaagggca....................  72      1  Fm3
......................................................................Cggacggagaacugauaagggca....................  37      1  Fm3
......................................................................Nggacggagaacugauaagggca....................  77      1  Fm3
......................................................................ugAacggagaacugauaagggca....................  32      1  Fm3
......................................................................uggacggagaacugauGagggca....................  343     1  Fm3
......................................................................uggacggaUaacugauaagggca....................  32      1  Fm3
......................................................................ugUacggagaacugauaagggca....................  58      1  Fm3
......................................................................uggacggaAaacugauaagggca....................  10      1  Fm3
......................................................................uggacggagaacugauaaUggca....................  69      1  Fm3
......................................................................uggacggagCacugauaagggca....................  20      1  Fm3
......................................................................uggacggagaacugauaagggGa....................  30      1  Fm3
......................................................................uggacggGgaacugauaagggca....................  41      1  Fm3
......................................................................uggacggagaacugauaaAggca....................  14      1  Fm3
......................................................................uggGcggagaacugauaagggca....................  34      1  Fm3
......................................................................uggacggagaaAugauaagggca....................  6       1  Fm3
......................................................................uggacggagaUcugauaagggca....................  14451   1  Fm3
......................................................................uggacggagaacugauaagggAa....................  3429    1  Fm3
......................................................................uggacggagaacugauUagggca....................  198     1  Fm3
......................................................................uggacggagaGcugauaagggca....................  257     1  Fm3
......................................................................uNgacggagaacugauaagggca....................  3       1  Fm3
......................................................................uggacggagaacugauaagggUa....................  363     1  Fm3
......................................................................uggacggagaacugCuaagggca....................  7       1  Fm3
......................................................................uggacggagaaGugauaagggca....................  6       1  Fm3
......................................................................uggacggagaacuCauaagggca....................  9       1  Fm3
......................................................................uggacAgagaacugauaagggca....................  33      1  Fm3
......................................................................uggacggagaacuAauaagggca....................  27      1  Fm3
......................................................................uggacggagaacugaAaagggca....................  2       1  Fm3
......................................................................uggaUggagaacugauaagggca....................  59      1  Fm3
......................................................................uggacgAagaacugauaagggca....................  9       1  Fm3
......................................................................uggacggagaCcugauaagggca....................  127     1  Fm3
......................................................................Gggacggagaacugauaagggca....................  4       1  Fm3
......................................................................uggacggUgaacugauaagggca....................  49      1  Fm3
......................................................................uggCcggagaacugauaagggca....................  19      1  Fm3
......................................................................uggacggagaacugauaaggCca....................  16      1  Fm3
......................................................................uggacggagaacugauaaCggca....................  8       1  Fm3
......................................................................uggacCgagaacugauaagggca....................  9       1  Fm3
......................................................................uggacggagaacugauaagAgca....................  26      1  Fm3
......................................................................uggacggagaacCgauaagggca....................  7       1  Fm3
......................................................................uggaAggagaacugauaagggca....................  19      1  Fm3
......................................................................Aggacggagaacugauaagggca....................  26      1  Fm3
```

```
                                  Star                             Mature
cauuugugacaugagaaguaaguuugucucguguccucuucacuuuuccauccuguguacucgagaacuggacggagaacugauaagggcauagggcagaccacaucaaca
......................................................................uggacggagaaUugauaagggca....................  22    1  Fm3
......................................................................uggacggagaacugauaaggAca....................  29    1  Fm3
......................................................................uggacgUagaacugauaagggca....................  59    1  Fm3
......................................................................uggacggagaacGgauaagggca....................  5     1  Fm3
......................................................................uggacggagaacugauaagCgca....................  9     1  Fm3
......................................................................uAgacggagaacugauaagggca....................  78    1  Fm3
......................................................................uggacggagaacugauaagggcG....................  246   1  Fm3
......................................................................uggaGggagaacugauaagggca....................  16    1  Fm3
......................................................................uggacggagaacugUuaagggca....................  26    1  Fm3
......................................................................uggUcggagaacugauaagggca....................  28    1  Fm3
......................................................................uggacggagaacugaCaagggca....................  24    1  Fm3
......................................................................uggacUgagaacugauaagggca....................  51    1  Fm3
......................................................................uggacggagaaUugauaagggcau...................  1     1  Fm3
......................................................................uggacggagaGcugauaagggcau...................  7     1  Fm3
......................................................................uggacNgagaacugauaagggcau...................  2     1  Fm3
......................................................................uggacggagaacugauaagggUau...................  434   1  Fm3
......................................................................uggacggagaacugauaagggcaG...................  63    1  Fm3
......................................................................uggacggagaacAgauaagggcau...................  1     1  Fm3
......................................................................uggacggagaacugauaagggcGu...................  5     1  Fm3
......................................................................uggaUggagaacugauaagggcau...................  2     1  Fm3
......................................................................uggacggagaacugauaagggcCu...................  4     1  Fm3
......................................................................uggacggagaacugauaagCgcau...................  1     1  Fm3
......................................................................uggacgUagaacugauaagggcau...................  3     1  Fm3
......................................................................uggacggagaacugauaUgggcau...................  19    1  Fm3
......................................................................ugUacggagaacugauaagggcau...................  1     1  Fm3
......................................................................uggacggagaacugauCagggcau...................  15    1  Fm3
......................................................................uUgacggagaacugauaagggcau...................  1     1  Fm3
......................................................................Nggacggagaacugauaagggcau...................  3     1  Fm3
......................................................................uggacggagaacugauaagUgcau...................  1     1  Fm3
......................................................................uggacggagaacugauaCgggcau...................  16    1  Fm3
......................................................................uggaAggagaacugauaagggcau...................  2     1  Fm3
......................................................................uggacggagaUcugauaagggcau...................  513   1  Fm3
......................................................................uggacggagaacugauaagggcaC...................  9     1  Fm3
......................................................................uggacggagGacugauaagggcau...................  4     1  Fm3
......................................................................uggacggagaacugauaGgggcau...................  26    1  Fm3
......................................................................uggGcggagaacugauaagggcau...................  1     1  Fm3
......................................................................uAgacggagaacugauaagggcau...................  2     1  Fm3
......................................................................uggacggagaacugauaagAgcau...................  1     1  Fm3
......................................................................uggacggagaacugauaagggAau...................  2005  1  Fm3
......................................................................uggacUgagaacugauaagggcau...................  2     1  Fm3
......................................................................uggacggagaacugauaagggcau...................  4351  0  Fm3
......................................................................uggacggagaacugauUagggcau...................  4     1  Fm3
......................................................................uggacggagaacugaCaagggcau...................  1     1  Fm3
......................................................................uggacggagaacCgauaagggcau...................  2     1  Fm3
......................................................................uggacggagaacugauGagggcau...................  7     1  Fm3
......................................................................ugCacggagaacugauaagggcau...................  2     1  Fm3
......................................................................uggacggagaacugauaagggcaA...................  231   1  Fm3
......................................................................uggacggagCacugauaagggcau...................  1     1  Fm3
......................................................................uggacggagaCcugauaagggcau...................  2     1  Fm3
......................................................................Aggacggagaacugauaagggcau...................  2     1  Fm3
......................................................................uggacggagaacuAauaagggcau...................  2     1  Fm3
......................................................................uggacggagaacugauaaUggcau...................  1     1  Fm3
......................................................................uggaUggagaacugauaagggcaua..................  1     1  Fm3
......................................................................uggacggagaacugauaagggcaAa..................  7     1  Fm3
......................................................................uggacggagaacugauaagggcaGa..................  2     1  Fm3
......................................................................uggacggagaUcugauaagggcaua..................  3     1  Fm3
......................................................................uggacggagaacugauaagggcauU..................  159   1  Fm3
......................................................................uggacggagaacugauaagggcUua..................  2     1  Fm3
......................................................................uggacggagaacugauaagggcauC..................  2     1  Fm3
......................................................................uggacggagaacugauaagggcaua..................  7     0  Fm3
......................................................................uggacggagaacugauaagggcauaU.................  12    1  Fm3
.......................................................................ggacggaAaacugauaag........................  1     1  Fm3
.......................................................................ggacggagaacugauaaA........................  68    1  Fm3
.......................................................................ggGcggagaacugauaag........................  1     1  Fm3
.......................................................................ggacggagaacugauaaC........................  1     1  Fm3
.......................................................................ggacggagaacugauaag........................  135   0  Fm3
.......................................................................ggacggagaacugauaaU........................  21    1  Fm3
.......................................................................ggacggagCacugauaag........................  1     1  Fm3
.......................................................................ggaUggagaacugauaag........................  1     1  Fm3
.......................................................................Ugacggagaacugauaag........................  6     1  Fm3
```

Star Mature

cauuugugacaugagaaguaaguuugucucguguccucuucacuuuuccauccugucuguacucgagaacuggacggagaacugauaagggcauagggcagaccacaucaaca			
....ggacggagaacugaAaag........................	1	1	Fm3
....ggacggagaacugauCag........................	1	1	Fm3
....ggacggagaacugaGaag........................	1	1	Fm3
....ggacggagaacugauaCg........................	1	1	Fm3
....ggacggagaGcugauaagg.......................	6	1	Fm3
....Ngacggagaacugauaagg.......................	1	1	Fm3
....ggacggagaacGgauaagg.......................	1	1	Fm3
....ggGcggagaacugauaagg.......................	1	1	Fm3
....ggacggaCaacugauaagg.......................	1	1	Fm3
....ggacggagaacugauaaUg.......................	2	1	Fm3
....ggacggagaacugauaagA.......................	108	1	Fm3
....ggacggagaCcugauaagg.......................	7	1	Fm3
....Ugacggagaacugauaagg.......................	9	1	Fm3
....ggacggagaacugauaGgg.......................	2	1	Fm3
....ggacggagaaUugauaagg.......................	1	1	Fm3
....ggacggagaacugauaUgg.......................	1	1	Fm3
....ggacggagaacugauaaAg.......................	1	1	Fm3
....ggacggagaacugauaagg.......................	317	0	Fm3
....ggaAggagaacugauaagg.......................	1	1	Fm3
....ggacggagaacCgauaagg.......................	2	1	Fm3
....ggacggagaacugauCagg.......................	3	1	Fm3
....ggacggaAaacugauaagg.......................	1	1	Fm3
....ggacggCgaacugauaagg.......................	1	1	Fm3
....ggacggagaacugaAaagg.......................	1	1	Fm3
....ggacggagaaAugauaagg.......................	3	1	Fm3
....ggacggagaacugauaagU.......................	8	1	Fm3
....ggacgAagaacugauaaggg......................	1	1	Fm3
....ggacggagaacugauaCggg......................	3	1	Fm3
....ggacggagaacugauaaAgg......................	1	1	Fm3
....ggacggagaacugauaaggA......................	253	1	Fm3
....ggacggagaacugauUaggg......................	2	1	Fm3
....ggacggagaacugauaaggg......................	386	0	Fm3
....ggacggagaaGugauaaggg......................	2	1	Fm3
....ggacggagaacugaAaaggg......................	1	1	Fm3
....ggacggagaacCgauaaggg......................	2	1	Fm3
....ggacggagaGcugauaaggg......................	4	1	Fm3
....ggacggUgaacugauaaggg......................	1	1	Fm3
....ggacggagaacugauaGggg......................	3	1	Fm3
....ggacggagaacugUuaaggg......................	1	1	Fm3
....ggacggagaacugauCaggg......................	5	1	Fm3
....ggacggagaacugauaaggC......................	5	1	Fm3
....ggacggagCacugauaaggg......................	1	1	Fm3
....Ugacggagaacugauaaggg......................	14	1	Fm3
....ggacggagaaAugauaaggg......................	1	1	Fm3
....ggacggagaacugGuaaggg......................	1	1	Fm3
....ggacggagUacugauaaggg......................	1	1	Fm3
....ggacggagaCcugauaaggg......................	5	1	Fm3
....ggacggagaacGgauaaggg......................	1	1	Fm3
....ggacggagaacugauaaggU......................	16	1	Fm3
....ggCcggagaacugauaagggc.....................	1	1	Fm3
....Ngacggagaacugauaagggc.....................	1	1	Fm3
....ggacggagaacugauGagggc.....................	2	1	Fm3
....ggacggagaacugauaagAgc.....................	3	1	Fm3
....ggacggagUacugauaagggc.....................	2	1	Fm3
....ggacggUgaacugauaagggc.....................	1	1	Fm3
....ggacggagaacugauaaggCc.....................	7	1	Fm3
....ggacggCgaacugauaagggc.....................	6	1	Fm3
....ggacggagaacGgauaagggc.....................	6	1	Fm3
....ggacggaCaacugauaagggc.....................	1	1	Fm3
....ggacggagaaUugauaagggc.....................	3	1	Fm3
....ggacggagGacugauaagggc.....................	1	1	Fm3
....ggaAggagaacugauaagggc.....................	4	1	Fm3
....ggacggagaacugauaaggAc.....................	8	1	Fm3
....ggacggagaacugauaagggA.....................	230	1	Fm3
....ggacggagaacugauaaAggc.....................	2	1	Fm3
....ggacggagaacuCauaagggc.....................	1	1	Fm3
....ggacggagaacugauaCgggc.....................	3	1	Fm3
....ggacggagaacugauaagggc.....................	1615	0	Fm3
....ggacggagaacAgauaagggc.....................	1	1	Fm3
....ggacggagaacugauUagggc.....................	2	1	Fm3

```
                                  Star                                  Mature
cauuugugacaugagaaguaaguuugucucguguccucuucacuuuuccauccugucuguacucgagaacuggacggagaacugauaagggcauagggcagaccacaucaaca
.......................................................................ggGcggagaacugauaagggc.....................   3     1   Fm3
.......................................................................ggacggagaacugUuaagggc.....................   1     1   Fm3
.......................................................................ggacggagaacugauaaggUc.....................   6     1   Fm3
.......................................................................ggacggagaGcugauaagggc.....................   3     1   Fm3
.......................................................................ggacggagaaGugauaagggc.....................   4     1   Fm3
.......................................................................ggacggagaacuUauaagggc.....................   2     1   Fm3
.......................................................................ggacggagaacugauaagCgc.....................   1     1   Fm3
.......................................................................ggacggagaaAugauaagggc.....................   7     1   Fm3
.......................................................................Ugacggagaacugauaagggc.....................   34    1   Fm3
.......................................................................ggaGggagaacugauaagggc.....................   1     1   Fm3
.......................................................................ggacggagaacugauaGgggc.....................   7     1   Fm3
.......................................................................ggacggaAaacugauaagggc.....................   2     1   Fm3
.......................................................................ggacggagaacugaAaagggc.....................   1     1   Fm3
.......................................................................ggacggagaacugauaaUggc.....................   1     1   Fm3
.......................................................................ggacggagaacugaGaagggc.....................   2     1   Fm3
.......................................................................ggacggagaacugauaagggU.....................   23    1   Fm3
.......................................................................ggacggagaacugauaUgggc.....................   1     1   Fm3
.......................................................................ggacggagaacCgauaagggc.....................   1     1   Fm3
.......................................................................ggacggagaCcugauaagggc.....................   4     1   Fm3
.......................................................................ggacggagCacugauaagggc.....................   15    1   Fm3
.......................................................................ggacCgagaacugauaagggc.....................   3     1   Fm3
.......................................................................ggGcggagaacugauaagggca....................   1     1   Fm3
.......................................................................ggacggagaacugCuaagggca....................   1     1   Fm3
.......................................................................ggacggagaacugauGagggca....................   1     1   Fm3
.......................................................................ggacggagaacugauaaUggca....................   2     1   Fm3
.......................................................................ggacggagaacugauaagggca....................   334   0   Fm3
.......................................................................ggacggagaacugauaaggUca....................   1     1   Fm3
.......................................................................ggacggagaaUugauaagggca....................   2     1   Fm3
.......................................................................ggacggagaacuAauaagggca....................   1     1   Fm3
.......................................................................ggacggagaacuCauaagggca....................   1     1   Fm3
.......................................................................ggacggagaacugauaagggAa....................   2     1   Fm3
.......................................................................ggacggagaacugauaagUgca....................   1     1   Fm3
.......................................................................Ugacggagaacugauaagggca....................   11    1   Fm3
.......................................................................ggaAggagaacugauaagggca....................   1     1   Fm3
.......................................................................ggacggagaacugauaaggCca....................   1     1   Fm3
.......................................................................ggacggagCacugauaagggca....................   3     1   Fm3
.......................................................................ggacggagaacugauCagggca....................   1     1   Fm3
.......................................................................ggacggagaUcugauaagggca....................   46    1   Fm3
.......................................................................ggacggagaacugauaaggAca....................   1     1   Fm3
.......................................................................ggacggagaaAugauaagggca....................   1     1   Fm3
.......................................................................ggacggagaacuUauaagggca....................   2     1   Fm3
.......................................................................ggacggagaacugauaagggAau...................   5     1   Fm3
.......................................................................ggacggagaUcugauaagggcau...................   3     1   Fm3
.......................................................................ggacggagaacugauaagggcau...................   11    0   Fm3
.......................................................................ggacggagaacugauaagggcaC...................   1     1   Fm3
.......................................................................ggacggagaacuCauaagggcau...................   1     1   Fm3
.......................................................................ggacggagaacugauaagggcaA...................   1     1   Fm3
........................................................................Uacggagaacugauaaggg......................   3     1   Fm3
........................................................................gacggagaacugauaaggg......................   3     0   Fm3
........................................................................gacggagaacugauaagggc.....................   9     0   Fm3
........................................................................gacggagaacugauaagggA.....................   1     1   Fm3
........................................................................Uacggagaacugauaagggc.....................   6     1   Fm3
........................................................................gacggagaaUugauaagggc.....................   2     1   Fm3
.........................................................................acggagaacugauaagggc.....................   1     0   Fm3
..........................................................................Uggagaacugauaagggc.....................   10    1   Fm3
..........................................................................cggagaacugauaagggc.....................   1     0   Fm3
..........................................................................Uggagaacugauaagggca....................   3     1   Fm3
...........................................................................ggagaacugauaagggca....................   2     0   Fm3
...........................................................................ggagaUcugauaagggca....................   1     1   Fm3

..................................ccucuucacuuuuccauccugu.........................................................   28    0   Mm2
..................................ccucuucacuuuuccauccuguU........................................................   1     1   Mm2
..................................ccucuucacuuuuccauccugucuguacuc.................................................   3     0   Mm2
..................................ccucuucacuuuuccauccugucuguacucgagaa............................................   1     0   Mm2
....................................ucuucacuuuuccauccugucuguacu..................................................   1     0   Mm2
..........................................cuuuuccauccugucuguacucgaga.............................................   1     0   Mm2
........................................................cuguacucgagaacuggacggagaacugauaagggc.....................   1     0   Mm2
..........................................................guacucgagaacuggacggagaacugauaagggc.....................   2     0   Mm2
.............................................................cucgagaacuggacggagaacugauaagggc.....................   1     0   Mm2
...............................................................cgagaacuggacggagaacugauaaggg......................   1     0   Mm2
```

Star Mature

cauuugugacaugagaaguaaguuugucucguguccucuucacuuuuccauccuguCuguacucgagaacuggacggagaacugauaagggcauagggcagaccacaucaaca			
....cgagaacuggacggagaacugauaagggc.....................	2	0	Mm2
....aUcuggacggagaacugauaagggc.....................	3	1	Mm2
....acuggacggagaacugauaag........................	1	0	Mm2
....acuggacggagaacugauaagg.......................	2	0	Mm2
....Ucuggacggagaacugauaaggg......................	2	1	Mm2
....Ucuggacggagaacugauaagggc.....................	8	1	Mm2
....Ucuggacggagaacugauaagggca....................	1	1	Mm2
....cuggacggagaacugaua..........................	1	0	Mm2
....cuggacggagaacugauaa.........................	3	0	Mm2
....Uuggacggagaacugauaag........................	1	1	Mm2
....cuggacggagaacugauaaU........................	1	1	Mm2
....cuggacggagaacugauaag........................	21	0	Mm2
....cuggacggagaacugauaaA........................	6	1	Mm2
....cuggacggagaacugauaagA.......................	11	1	Mm2
....cuggacggagaacugauaagg.......................	71	0	Mm2
....cuggacggagaacugauUagg.......................	1	1	Mm2
....cuggacggagaacugaCaagg.......................	1	1	Mm2
....cuggacggagaacugauaagU.......................	1	1	Mm2
....cuggGcggagaacugauaaggg......................	1	1	Mm2
....cuggacggagaacugUuaaggg......................	1	1	Mm2
....cuggacggagaacugauaaggA......................	8	1	Mm2
....cuggacggagaacugauaaggg......................	97	0	Mm2
....cuggacggagaacugauaaggU......................	16	1	Mm2
....cuggacggagaacugauaaggC......................	1	1	Mm2
....Auggacggagaacugauaagggc.....................	2	1	Mm2
....Uuggacggagaacugauaagggc.....................	1	1	Mm2
....cAggacggagaacugauaagggc.....................	1	1	Mm2
....cuggacggagaacugauaagggA.....................	2	1	Mm2
....cuggacggagaacugauGagggc.....................	1	1	Mm2
....cuggacggagaacugauaagggc.....................	233	0	Mm2
....cuggacggagaacugaCaagggc.....................	1	1	Mm2
....cugAacggagaacugauaagggc.....................	1	1	Mm2
....cuggacggagaacugauaagggU.....................	5	1	Mm2
....cuggacggagaacugauaGgggc.....................	1	1	Mm2
....cuggacUgagaacugauaagggca....................	1	1	Mm2
....cuggacggagaacugauaagggca....................	16	0	Mm2
....cuggacggagaacugauaagggcC...................	2	1	Mm2
....cuggacggagaUcugauaagggca....................	2	1	Mm2
....uggacggagaacugauaG.........................	9	1	Mm2
....uggacggagaacugauaU.........................	39	1	Mm2
....uggacggagaacugauGa.........................	2	1	Mm2
....uggacggagaacugauUa.........................	6	1	Mm2
....ugCacggagaacugauaa.........................	2	1	Mm2
....uggacggUgaacugauaa.........................	2	1	Mm2
....uggacggagaacugauaC.........................	8	1	Mm2
....uggUcggagaacugauaa.........................	2	1	Mm2
....uggacgCagaacugauaa.........................	1	1	Mm2
....uggacggGgaacugauaa.........................	1	1	Mm2
....uggacggagaacugauaa.........................	1306	0	Mm2
....uggacggagaacugUuaa.........................	1	1	Mm2
....uggGcggagaacugauaa.........................	1	1	Mm2
....uggacggagaGcugauaa.........................	1	1	Mm2
....uggacggagaacugauCa.........................	1	1	Mm2
....uggacggagaacugaAaa.........................	136	1	Mm2
....uggacggagGacugauaag........................	12	1	Mm2
....uggacggagaacugauaag........................	8357	0	Mm2
....ugCacggagaacugauaag........................	1	1	Mm2
....uggacggagaacugauaaA........................	3696	1	Mm2
....uggacggagaacugauaaU........................	1753	1	Mm2
....uggacggagaacugauGag........................	6	1	Mm2
....uggacggagaacugauaGg........................	24	1	Mm2
....uggacggagaacuCauaag........................	1	1	Mm2
....uggacggagUacugauaag........................	2	1	Mm2
....uggacggagaacuAauaag........................	2	1	Mm2
....uggGcggagaacugauaag........................	1	1	Mm2
....uggacNgagaacugauaag........................	1	1	Mm2
....uggacggagaacugGuaag........................	1	1	Mm2
....uggacgUagaacugauaag........................	1	1	Mm2
....uUgacggagaacugauaag........................	1	1	Mm2
....uggacggaUaacugauaag........................	2	1	Mm2

Star **Mature**

cauuugugacaugagaaguaaguuugucucguguccucuucacuuuuccauccugucuguacucgagaacuggacggagaacugauaagggcauagggcagaccacaucaaca			
....uggacggagaacugauaUg........................	26	1	Mm2
....uggacggagaacugaAaag........................	2	1	Mm2
....ugAacggagaacugauaag........................	2	1	Mm2
....uggacUgagaacugauaag........................	3	1	Mm2
....uggacggagaacCgauaag........................	3	1	Mm2
....uggacggagaacugaGaag........................	1	1	Mm2
....uggaUggagaacugauaag........................	1	1	Mm2
....uggUcggagaacugauaag........................	1	1	Mm2
....uggacggagaCcugauaag........................	10	1	Mm2
....uggacggagaacugauUag........................	12	1	Mm2
....uggaAggagaacugauaag........................	1	1	Mm2
....uggacggagaacugauCag........................	40	1	Mm2
....uggacggagaacugauaCg........................	33	1	Mm2
....uggacAgagaacugauaag........................	1	1	Mm2
....Cggacggagaacugauaag........................	2	1	Mm2
....uggacggagaacugUuaag........................	1	1	Mm2
....uggacggagaacugaCaag........................	1	1	Mm2
....uggacgCagaacugauaag........................	1	1	Mm2
....Nggacggagaacugauaag........................	4	1	Mm2
....Aggacggagaacugauaag........................	2	1	Mm2
....uggacggagaacugauaaC........................	11	1	Mm2
....uNgacggagaacugauaag........................	1	1	Mm2
....uCgacggagaacugauaag........................	1	1	Mm2
....uggacggGgaacugauaag........................	2	1	Mm2
....uAgacggagaacugauaag........................	4	1	Mm2
....uggacggagaGcugauaag........................	20	1	Mm2
....uggacggUgaacugauaag........................	1	1	Mm2
....Nggacggagaacugauaagg.......................	7	1	Mm2
....uggacgUagaacugauaagg.......................	8	1	Mm2
....uggacggagaacugaCaagg.......................	3	1	Mm2
....uUgacggagaacugauaagg.......................	5	1	Mm2
....uggacggagaacugauaagA.......................	2256	1	Mm2
....uggacggagaacugauCagg.......................	35	1	Mm2
....ugUacggagaacugauaagg.......................	6	1	Mm2
....uggaGggagaacugauaagg.......................	1	1	Mm2
....uggacNgagaacugauaagg.......................	3	1	Mm2
....ugCacggagaacugauaagg.......................	1	1	Mm2
....uggacggagaacugauGagg.......................	16	1	Mm2
....uggacggagaacugauaaAg.......................	116	1	Mm2
....uggacggagaacugauaCgg.......................	71	1	Mm2
....uggacAgagaacugauaagg.......................	4	1	Mm2
....uggacgNagaacugauaagg.......................	3	1	Mm2
....uggacggagaaUugauaagg.......................	3	1	Mm2
....uggacggagaacugauaGgg.......................	90	1	Mm2
....uggacggagaacCgauaagg.......................	2	1	Mm2
....uggaUggagaacugauaagg.......................	1	1	Mm2
....uggacggagaacugauaaUg.......................	1	1	Mm2
....uggaAggagaacugauaagg.......................	2	1	Mm2
....uAgacggagaacugauaagg.......................	13	1	Mm2
....Gggacggagaacugauaagg.......................	1	1	Mm2
....ugAacggagaacugauaagg.......................	5	1	Mm2
....uggacggagCacugauaagg.......................	2	1	Mm2
....uggacggagaacGgauaagg.......................	1	1	Mm2
....uggacggaAaacugauaagg.......................	1	1	Mm2
....uggacggagaacugauaagU.......................	554	1	Mm2
....uggacggagaacugUuaagg.......................	1	1	Mm2
....uggacUgagaacugauaagg.......................	11	1	Mm2
....uggacggagaacuAauaagg.......................	1	1	Mm2
....uggacggagaacuCauaagg.......................	4	1	Mm2
....uggUcggagaacugauaagg.......................	1	1	Mm2
....uggCcggagaacugauaagg.......................	3	1	Mm2
....uggacggagaacuUauaagg.......................	1	1	Mm2
....uggacggagaacugauaUgg.......................	63	1	Mm2
....uggacggagaacugauaagg.......................	18110	0	Mm2
....uggacggGgaacugauaagg.......................	1	1	Mm2
....uggGcggagaacugauaagg.......................	4	1	Mm2
....uggacggaUaacugauaagg.......................	4	1	Mm2
....uggacggagaacugGuaagg.......................	2	1	Mm2
....uggacggagaacugauUagg.......................	30	1	Mm2
....uggacgAagaacugauaagg.......................	1	1	Mm2

```
                                   Star                              Mature
cauuugugacaugagaaguaaguuugucucguguccucuucacuuuuccauccugucuguacucgagaacuggacggagaacugauaagggcauagggcagaccacaucaaca
......................................................................uggacggagaCcugauaagg.......................   60     1   Mm2
......................................................................Aggacggagaacugauaagg.......................   32     1   Mm2
......................................................................uggacggagaGcugauaagg.......................   103    1   Mm2
......................................................................uggacggagaacugauaagC.......................   10     1   Mm2
......................................................................uCgacggagaacugauaagg.......................   5      1   Mm2
......................................................................uggacggUgaacugauaagg.......................   4      1   Mm2
......................................................................uggacggagUacugauaagg.......................   3      1   Mm2
......................................................................uggacggagGacugauaagg.......................   24     1   Mm2
......................................................................Cggacggagaacugauaagg.......................   4      1   Mm2
......................................................................uggacggagaacGgauaaggg......................   4      1   Mm2
......................................................................uggacCgagaacugauaaggg......................   1      1   Mm2
......................................................................uggacggagaacugauaaggg......................   41055  0   Mm2
......................................................................uggacggagaacugauaaAgg......................   5      1   Mm2
......................................................................uggacggagaacugauGaggg......................   49     1   Mm2
......................................................................uggacggagaacugauaGggg......................   262    1   Mm2
......................................................................uUgacggagaacugauaaggg......................   12     1   Mm2
......................................................................uggacggagaacugauaaUgg......................   5      1   Mm2
......................................................................uggacggagaacugauaaggU......................   899    1   Mm2
......................................................................uggacggUgaacugauaaggg......................   8      1   Mm2
......................................................................uggCcggagaacugauaaggg......................   3      1   Mm2
......................................................................uggacggagaacugauaagCg......................   3      1   Mm2
......................................................................uggacggagUacugauaaggg......................   15     1   Mm2
......................................................................uggacggagaacugGuaaggg......................   6      1   Mm2
......................................................................uggacggagaacugauaaCgg......................   1      1   Mm2
......................................................................uggacggagaacugUuaaggg......................   6      1   Mm2
......................................................................uggacggagaacugauaCggg......................   173    1   Mm2
......................................................................ugUacggagaacugauaaggg......................   8      1   Mm2
......................................................................uggUcggagaacugauaaggg......................   2      1   Mm2
......................................................................uggacggagaacuUauaaggg......................   11     1   Mm2
......................................................................Cggacggagaacugauaaggg......................   8      1   Mm2
......................................................................uggaUggagaacugauaaggg......................   9      1   Mm2
......................................................................uggacggagaacugauaUggg......................   129    1   Mm2
......................................................................uggacggagaacCgauaaggg......................   7      1   Mm2
......................................................................uggacgCagaacugauaaggg......................   5      1   Mm2
......................................................................uggacggagaacugauaaggA......................   5255   1   Mm2
......................................................................uggacggagaacugaCaaggg......................   4      1   Mm2
......................................................................uggacggCgaacugauaaggg......................   4      1   Mm2
......................................................................uggacggaCaacugauaaggg......................   4      1   Mm2
......................................................................Aggacggagaacugauaaggg......................   41     1   Mm2
......................................................................uggacUgagaacugauaaggg......................   14     1   Mm2
......................................................................uggacggagaGcugauaaggg......................   158    1   Mm2
......................................................................uAgacggagaacugauaaggg......................   11     1   Mm2
......................................................................uggacgNagaacugauaaggg......................   4      1   Mm2
......................................................................uggacAgagaacugauaaggg......................   12     1   Mm2
......................................................................uggacggagaaGugauaaggg......................   1      1   Mm2
......................................................................uggacggGgaacugauaaggg......................   10     1   Mm2
......................................................................uggacggagaCcugauaaggg......................   90     1   Mm2
......................................................................uggacggagaacugCuaaggg......................   5      1   Mm2
......................................................................Nggacggagaacugauaaggg......................   22     1   Mm2
......................................................................uggacggagaacuCauaaggg......................   3      1   Mm2
......................................................................uggacggagaaAugauaaggg......................   3      1   Mm2
......................................................................uggacggagaacugauaagUg......................   6      1   Mm2
......................................................................uggaGggagaacugauaaggg......................   1      1   Mm2
......................................................................uggacgAagaacugauaaggg......................   9      1   Mm2
......................................................................uggacggagCacugauaaggg......................   3      1   Mm2
......................................................................uggacgUagaacugauaaggg......................   10     1   Mm2
......................................................................uggacggagGacugauaaggg......................   50     1   Mm2
......................................................................uggacggagaacugauaagAg......................   15     1   Mm2
......................................................................uggacggagaacugauaaggC......................   215    1   Mm2
......................................................................uggacggaAaacugauaaggg......................   3      1   Mm2
......................................................................uggGcggagaacugauaaggg......................   7      1   Mm2
......................................................................ugCacggagaacugauaaggg......................   4      1   Mm2
......................................................................uggacNgagaacugauaaggg......................   9      1   Mm2
......................................................................uggacggagaacuAauaaggg......................   7      1   Mm2
......................................................................uggaAggagaacugauaaggg......................   3      1   Mm2
......................................................................ugAacggagaacugauaaggg......................   14     1   Mm2
......................................................................uggacggagaacugauUaggg......................   62     1   Mm2
......................................................................uggacggagaacugauCaggg......................   83     1   Mm2
......................................................................Gggacggagaacugauaaggg......................   1      1   Mm2
......................................................................uggacggagaaUugauaaggg......................   16     1   Mm2
```

Star **Mature**

```
cauuugugacaugagaaguaaguuugucucguguccucuucacuuuuccauccugucuguacucgagaacuggacggagaacugauaagggcauagggcagaccacaucaaca
......................................................................uggacggaUaacugauaaggg......................    8       1    Mm2
......................................................................uggacggagaacuCauaagggc.....................    36      1    Mm2
......................................................................uggacggagaacugauaaggUc.....................    123     1    Mm2
......................................................................uggacggagaacugaCaagggc.....................    45      1    Mm2
......................................................................uggacggagaacugaAaagggc.....................    14      1    Mm2
......................................................................uggacggagaacugauaagggU.....................    3929    1    Mm2
......................................................................uggacggagaacugauCagggc.....................    1033    1    Mm2
......................................................................uggacggagaacCgauaagggc.....................    74      1    Mm2
......................................................................uggacggagaacugauaagCgc.....................    25      1    Mm2
......................................................................uggacggagaacugauaGgggc.....................    5049    1    Mm2
......................................................................uggacggaCaacugauaagggc.....................    27      1    Mm2
......................................................................uggacggagaacugauaaUggc.....................    76      1    Mm2
......................................................................uggGcggagaacugauaagggc.....................    131     1    Mm2
......................................................................uggacgAagaacugauaagggc.....................    63      1    Mm2
......................................................................uggaUggagaacugauaagggc.....................    99      1    Mm2
......................................................................uggacggagCacugauaagggc.....................    55      1    Mm2
......................................................................uggUcggagaacugauaagggc.....................    89      1    Mm2
......................................................................uggacggagaaGugauaagggc.....................    14      1    Mm2
......................................................................Nggacggagaacugauaagggc.....................    258     1    Mm2
......................................................................uggacAgagaacugauaagggc.....................    82      1    Mm2
......................................................................uggacggagaacugauUagggc.....................    638     1    Mm2
......................................................................uggacggagaacugauaagAgc.....................    84      1    Mm2
......................................................................uggacggaAaacugauaagggc.....................    31      1    Mm2
......................................................................uUgacggagaacugauaagggc.....................    92      1    Mm2
......................................................................ugNacggagaacugauaagggc.....................    5       1    Mm2
......................................................................uggacggagGacugauaagggc.....................    998     1    Mm2
......................................................................uggacggagaacAgauaagggc.....................    20      1    Mm2
......................................................................uCgacggagaacugauaagggc.....................    37      1    Mm2
......................................................................uggacggGgaacugauaagggc.....................    113     1    Mm2
......................................................................uggacgUagaacugauaagggc.....................    127     1    Mm2
......................................................................uggacggagaacugauaaCggc.....................    14      1    Mm2
......................................................................ugAacggagaacugauaagggc.....................    103     1    Mm2
......................................................................uggacggagaacugauaNgggc.....................    1       1    Mm2
......................................................................ugCacggagaacugauaagggc.....................    46      1    Mm2
......................................................................uAgacggagaacugauaagggc.....................    244     1    Mm2
......................................................................uggacggagaaUugauaagggc.....................    79      1    Mm2
......................................................................uggCcggagaacugauaagggc.....................    116     1    Mm2
......................................................................Gggacggagaacugauaagggc.....................    27      1    Mm2
......................................................................uggacggagaCcugauaagggc.....................    435     1    Mm2
......................................................................uggacggagaacugauaagggc.....................    505969  0    Mm2
......................................................................uggacUgagaacugauaagggc.....................    84      1    Mm2
......................................................................uggacggagaacugauGagggc.....................    745     1    Mm2
......................................................................uggacNgagaacugauaagggc.....................    79      1    Mm2
......................................................................uNgacggagaacugauaagggc.....................    7       1    Mm2
......................................................................uggacggagaacugUuaagggc.....................    68      1    Mm2
......................................................................uggacggCgaacugauaagggc.....................    23      1    Mm2
......................................................................uggacggagaacugauaagUgc.....................    132     1    Mm2
......................................................................uggacggagaacugauaCgggc.....................    1933    1    Mm2
......................................................................uggaGggagaacugauaagggc.....................    18      1    Mm2
......................................................................uggacggagaacugCuaagggc.....................    17      1    Mm2
......................................................................uggacggagaacugauaagggA.....................    11213   1    Mm2
......................................................................uggacggagaacuAauaagggc.....................    105     1    Mm2
......................................................................uggacggagaacGgauaagggc.....................    22      1    Mm2
......................................................................uggacggagaacugauaagggG.....................    327     1    Mm2
......................................................................uggacggagaacugauaaggAc.....................    162     1    Mm2
......................................................................uggacggagaacugauaaAggc.....................    46      1    Mm2
......................................................................ugUacggagaacugauaagggc.....................    92      1    Mm2
......................................................................uggacCgagaacugauaagggc.....................    13      1    Mm2
......................................................................Aggacggagaacugauaagggc.....................    129     1    Mm2
......................................................................uggacggagaacugaGaagggc.....................    8       1    Mm2
......................................................................uggaAggagaacugauaagggc.....................    29      1    Mm2
......................................................................uggacgNagaacugauaagggc.....................    22      1    Mm2
......................................................................uggacggagaaAugauaagggc.....................    14      1    Mm2
......................................................................uggacggagUacugauaagggc.....................    112     1    Mm2
......................................................................Cggacggagaacugauaagggc.....................    64      1    Mm2
......................................................................uggacggagaacugauaaggCc.....................    101     1    Mm2
......................................................................uggacggagaGcugauaagggc.....................    844     1    Mm2
......................................................................uggacggaUaacugauaagggc.....................    93      1    Mm2
......................................................................uggacggagaacuUauaagggc.....................    45      1    Mm2
......................................................................uggacgCagaacugauaagggc.....................    18      1    Mm2
```

Star **Mature**

```
cauuugugacaugagaaguaaguuugucucguguccucuucacuuuuccauccuguc uguacucgagaacuggacggagaacugauaagggcauagggcagaccacaucaaca
```

```
......................................................................uggacggUgaacugauaagggc.....................	113	1	Mm2
......................................................................uggacggagaacugGuaagggc.....................	51	1	Mm2
......................................................................uggacggagaacugauaUgggc.....................	1591	1	Mm2
......................................................................Aggacggagaacugauaagggca....................	3	1	Mm2
......................................................................uggacggagaacugauaagCgca....................	4	1	Mm2
......................................................................uggacAgagaacugauaagggca....................	3	1	Mm2
......................................................................uggacggagaacugauaagAgca....................	2	1	Mm2
......................................................................uggacggagaGcugauaagggca....................	60	1	Mm2
......................................................................uggacggagaacuCauaagggca....................	4	1	Mm2
......................................................................uggacggagaacugauaagggcG....................	1203	1	Mm2
......................................................................uggacggagaacugauGagggca....................	47	1	Mm2
......................................................................uggacCgagaacugauaagggca....................	1	1	Mm2
......................................................................uggacggagaCcugauaagggca....................	31	1	Mm2
......................................................................uggacggagGacugauaagggca....................	39	1	Mm2
......................................................................uggacggagaacugauaagggca....................	36577	0	Mm2
......................................................................uAgacggagaacugauaagggca....................	14	1	Mm2
......................................................................uggacggCgaacugauaagggca....................	3	1	Mm2
......................................................................uggacggaAaacugauaagggca....................	3	1	Mm2
......................................................................uCgacggagaacugauaagggca....................	4	1	Mm2
......................................................................uggacggagaacugUuaagggca....................	6	1	Mm2
......................................................................uggacggagaacCgauaagggca....................	5	1	Mm2
......................................................................uggaUggagaacugauaagggca....................	10	1	Mm2
......................................................................Nggacggagaacugauaagggca....................	24	1	Mm2
......................................................................uggacggagaacugauaagggcC....................	305	1	Mm2
......................................................................uggacggagaacugauCagggca....................	64	1	Mm2
......................................................................uggacggagaacugauaaAggca....................	5	1	Mm2
......................................................................uggacggagaacGgauaagggca....................	2	1	Mm2
......................................................................uggacggagaacuAauaagggca....................	9	1	Mm2
......................................................................uggacggagUacugauaagggca....................	7	1	Mm2
......................................................................uggCcggagaacugauaagggca....................	9	1	Mm2
......................................................................uggacggagaacugCuaagggca....................	2	1	Mm2
......................................................................uggacggagaacuUauaagggca....................	5	1	Mm2
......................................................................uUgacggagaacugauaagggca....................	7	1	Mm2
......................................................................Cggacggagaacugauaagggca....................	6	1	Mm2
......................................................................uggacggUgaacugauaagggca....................	11	1	Mm2
......................................................................uggacgAagaacugauaagggca....................	6	1	Mm2
......................................................................uggacggagaacAgauaagggca....................	1	1	Mm2
......................................................................uggacNgagaacugauaagggca....................	8	1	Mm2
......................................................................uggacggagaacugGuaagggca....................	3	1	Mm2
......................................................................uggacggagaacugauaaUggca....................	9	1	Mm2
......................................................................uggaAggagaacugauaagggca....................	2	1	Mm2
......................................................................uggacUgagaacugauaagggca....................	7	1	Mm2
......................................................................uggacgUagaacugauaagggca....................	6	1	Mm2
......................................................................uggacggaUaacugauaagggca....................	10	1	Mm2
......................................................................uggacggagaUcugauaagggca....................	2228	1	Mm2
......................................................................uggGcggagaacugauaagggca....................	11	1	Mm2
......................................................................ugAacggagaacugauaagggca....................	10	1	Mm2
......................................................................ugUacggagaacugauaagggca....................	8	1	Mm2
......................................................................uggacggagaacugauaUgggca....................	121	1	Mm2
......................................................................uggacggagaacugauaagUgca....................	12	1	Mm2
......................................................................uggacggagaaUugauaagggca....................	7	1	Mm2
......................................................................uggacggagaacugauaCgggca....................	145	1	Mm2
......................................................................uggacgNagaacugauaagggca....................	1	1	Mm2
......................................................................uggacggagaacugauaaggAca....................	10	1	Mm2
......................................................................uggacggagaacugauaaggUca....................	12	1	Mm2
......................................................................uggacggagCacugauaagggca....................	4	1	Mm2
......................................................................uggacggagaacugauaGgggca....................	238	1	Mm2
......................................................................uggacgCagaacugauaagggca....................	1	1	Mm2
......................................................................uggacggagaacugauaagggAa....................	612	1	Mm2
......................................................................uggacggGgaacugauaagggca....................	9	1	Mm2
......................................................................uggacggagaacugauUagggca....................	58	1	Mm2
......................................................................uggUcggagaacugauaagggca....................	7	1	Mm2
......................................................................uggacggagaacugaAaagggca....................	1	1	Mm2
......................................................................uggacggagaacugauaagggUa....................	161	1	Mm2
......................................................................ugCacggagaacugauaagggca....................	3	1	Mm2
......................................................................uggacggagaacugaCaagggca....................	3	1	Mm2
......................................................................uggacggagaacugauaaggCca....................	27	1	Mm2
......................................................................uggacggagaacugauaagggGa....................	3	1	Mm2
......................................................................Nggacggagaacugauaagggcau...................	1	1	Mm2
......................................................................Cggacggagaacugauaagggcau...................	1	1	Mm2
```

```
                                                  Star                              Mature
cauuugugacaugagaaguaaguuugucucguguccucuucacuuuuccauccuguucuguacucgagaacuggacggagaacugauaagggcauagggcagaccacaucaaca
......................................................................uggacggagaacugauaagggUau...................   175    1    Mm2
......................................................................uggacggagaacugauaagggcaG...................   52     1    Mm2
......................................................................uggacggagaacugauaagggcaC...................   27     1    Mm2
......................................................................uggacggagaacugauUagggcau...................   3      1    Mm2
......................................................................uggacggagaacugauaagggcau...................   2426   0    Mm2
......................................................................uggacggagaacugauaagggAau...................   449    1    Mm2
......................................................................uggaAggagaacugauaagggcau...................   1      1    Mm2
......................................................................uggacggagGacugauaagggcau...................   1      1    Mm2
......................................................................uggacggagaaUugauaagggcau...................   1      1    Mm2
......................................................................uAgacggagaacugauaagggcau...................   1      1    Mm2
......................................................................uggacggagaCcugauaagggcau...................   2      1    Mm2
......................................................................uggacggagaacugauaagAgcau...................   1      1    Mm2
......................................................................uggacggagaacugauaagggcaA...................   92     1    Mm2
......................................................................uggacggagCacugauaagggcau...................   1      1    Mm2
......................................................................uggCcggagaacugauaagggcau...................   1      1    Mm2
......................................................................uggacggagaacugauaagggcGu...................   27     1    Mm2
......................................................................uggacggagaacugauaagUgcau...................   2      1    Mm2
......................................................................uggacggagaacugauaaggCcau...................   1      1    Mm2
......................................................................uggacggagaacugauaCgggcau...................   8      1    Mm2
......................................................................uggacggagaacugauCagggcau...................   2      1    Mm2
......................................................................uggacggagaacugauaGgggcau...................   23     1    Mm2
......................................................................uggacggagaGcugauaagggcau...................   7      1    Mm2
......................................................................uggacggagaacugauaUgggcau...................   11     1    Mm2
......................................................................ugCacggagaacugauaagggcau...................   1      1    Mm2
......................................................................uggacggagaacugauaagggGau...................   1      1    Mm2
......................................................................uggacggagaacugauaaUggcau...................   1      1    Mm2
......................................................................uggacggagaacugauaagggcCu...................   20     1    Mm2
......................................................................uggacggagaUcugauaagggcau...................   187    1    Mm2
......................................................................uggacggUgaacugauaagggcau...................   2      1    Mm2
......................................................................uggacggaUaacugauaagggcau...................   1      1    Mm2
......................................................................uggacggagaacugauGagggcau...................   4      1    Mm2
......................................................................uggacggagaacugauaagggcaua..................   7      0    Mm2
......................................................................uggacggagaacugauaagggcauG..................   1      1    Mm2
......................................................................uggacggagaacugauaagggcUua..................   8      1    Mm2
......................................................................uggacggagaacugauaagggcauU..................   313    1    Mm2
......................................................................uggacggagaacugauaagggcaAa..................   8      1    Mm2
......................................................................uggacggagaacugauaagggcauC..................   5      1    Mm2
......................................................................uggacggagaacugauaagggcauUg.................   1      1    Mm2
......................................................................uggacggagaacugauaagggcauaU.................   9      1    Mm2
.......................................................................ggacggagaAugauaag.........................   1      1    Mm2
.......................................................................ggacggagaacugauaaU........................   1      1    Mm2
.......................................................................ggacggagaacugauaaA........................   8      1    Mm2
.......................................................................ggacggagaacugauaag........................   17     0    Mm2
.......................................................................ggacggagaGcugauaagg.......................   1      1    Mm2
.......................................................................ggacggagaacugauaUgg.......................   1      1    Mm2
.......................................................................ggacggagaacugauaagA.......................   5      1    Mm2
.......................................................................Ugacggagaacugauaagg.......................   2      1    Mm2
.......................................................................ggacggagaacugauaagg.......................   47     0    Mm2
.......................................................................ggGcggagaacugauaagg.......................   1      1    Mm2
.......................................................................ggacggagaacugauaagU.......................   1      1    Mm2
.......................................................................ggacggagaacuUauaagg.......................   1      1    Mm2
.......................................................................ggacggaAaacugauaaggg......................   1      1    Mm2
.......................................................................ggacggagaaAugauaaggg......................   2      1    Mm2
.......................................................................ggacggagaacugauaaggA......................   11     1    Mm2
.......................................................................ggacggagaacugauaaggg......................   48     0    Mm2
.......................................................................ggacggagaacugaCaaggg......................   1      1    Mm2
.......................................................................Ugacggagaacugauaaggg......................   1      1    Mm2
.......................................................................ggacggagaacugauaaggU......................   3      1    Mm2
.......................................................................ggacggagUacugauaaggg......................   1      1    Mm2
.......................................................................ggacggagaacugauaaggC......................   2      1    Mm2
.......................................................................ggacgAagaacugauaagggc.....................   1      1    Mm2
.......................................................................ggacggagaacugauCagggc.....................   3      1    Mm2
.......................................................................ggacggGgaacugauaagggc.....................   1      1    Mm2
.......................................................................ggacggagCacugauaagggc.....................   8      1    Mm2
.......................................................................ggacggagaacugauaGgggc.....................   12     1    Mm2
.......................................................................ggacggagaGcugauaagggc.....................   1      1    Mm2
.......................................................................ggacgUagaacugauaagggc.....................   1      1    Mm2
.......................................................................ggacggagaacugauaaggCc.....................   5      1    Mm2
.......................................................................ggacggagUacugauaagggc.....................   1      1    Mm2
.......................................................................ggacggagaaGugauaagggc.....................   4      1    Mm2
```

```
                                  Star                                  Mature
cauuugugacaugagaaguaaguuugucucguguccucuucacuuuuccauccugucuguacucgagaacuggacggagaacugauaagggcauagggcagaccacaucaaca
.......................................................................ggacggagaacugauaCgggc.....................  4    1  Mm2
.......................................................................gAacggagaacugauaagggc.....................  2    1  Mm2
.......................................................................ggacggaAaacugauaagggc.....................  1    1  Mm2
.......................................................................ggacggagaacugauaaUggc.....................  1    1  Mm2
.......................................................................ggUcggagaacugauaagggc.....................  2    1  Mm2
.......................................................................ggacggagaacugauaagggU.....................  9    1  Mm2
.......................................................................ggacggagaacugauaagggc.....................  887  0  Mm2
.......................................................................ggacggagaaUugauaagggc.....................  3    1  Mm2
.......................................................................ggaGggagaacugauaagggc.....................  1    1  Mm2
.......................................................................ggacggagaacugauaaggUc.....................  3    1  Mm2
.......................................................................ggacCgagaacugauaagggc.....................  1    1  Mm2
.......................................................................ggacggagaaAugauaagggc.....................  2    1  Mm2
.......................................................................ggacggagaacugauaaggAc.....................  2    1  Mm2
.......................................................................ggacggagaacugauaagCgc.....................  2    1  Mm2
.......................................................................ggacggagaacugauaagggA.....................  26   1  Mm2
.......................................................................ggacggagaacugauaUgggc.....................  2    1  Mm2
.......................................................................ggacggagaacugauaagUgc.....................  1    1  Mm2
.......................................................................ggacggagaacugauaagAgc.....................  1    1  Mm2
.......................................................................ggacggagaacuUauaagggc.....................  2    1  Mm2
.......................................................................Ugacggagaacugauaagggc.....................  20   1  Mm2
.......................................................................ggacggagaCcugauaagggc.....................  1    1  Mm2
.......................................................................ggaAggagaacugauaagggc.....................  1    1  Mm2
.......................................................................ggGcggagaacugauaagggc.....................  2    1  Mm2
.......................................................................Ugacggagaacugauaagggca....................  2    1  Mm2
.......................................................................ggacggagaaAugauaagggca....................  1    1  Mm2
.......................................................................ggacggagaacugauaagAgca....................  1    1  Mm2
.......................................................................ggacggagaacugauaagggcG....................  3    1  Mm2
.......................................................................ggacggagaacugauaagggcC....................  1    1  Mm2
.......................................................................ggacggagaUcugauaagggca....................  4    1  Mm2
.......................................................................ggacggagaacugauaagggca....................  79   0  Mm2
.......................................................................ggacggagaacugauaagggAau...................  2    1  Mm2
.......................................................................ggaAggagaacugauaagggcau...................  1    1  Mm2
.......................................................................ggacggagaacugauaagggcau...................  3    0  Mm2
.......................................................................ggacggagaaUugauaagggcau...................  1    1  Mm2
........................................................................gacggagaacugauaagggc.....................  1    0  Mm2
........................................................................gacggagaacugauaagggcauU..................  1    1  Mm2
.........................................................................acggagaacugauaaggg......................  1    0  Mm2
.........................................................................acUgagaacugauaagggc.....................  1    1  Mm2
.........................................................................acggagaacugauaagggc.....................  3    0  Mm2
..........................................................................Uggagaacugauaagggc.....................  3    1  Mm2
..........................................................................cggagaacugauaagggc.....................  1    0  Mm2
...........................................................................ggagaacugauaagggca....................  1    0  Mm2

....................................................................Ucuggacggagaacugauaagggc.....................  1    1  Mm1
.....................................................................cuggacggagaacugauaag........................  1    0  Mm1
.....................................................................cuggacggagaacugauaagg.......................  43   0  Mm1
.....................................................................cuggacggagaacugauaagA.......................  7    1  Mm1
.....................................................................cuggacggagCacugauaagg.......................  1    1  Mm1
.....................................................................cuggacggagaacugauaaggg......................  17   0  Mm1
.....................................................................cuggacggagCacugauaaggg......................  1    1  Mm1
.....................................................................cuggacggagaacugauaagggc.....................  45   0  Mm1
.....................................................................cuggacggagCacugauaagggc.....................  1    1  Mm1
.....................................................................cugUacggagaacugauaagggc.....................  1    1  Mm1
.....................................................................cuggacggagaacugauaagggca....................  5    0  Mm1
......................................................................uggacgAagaacugauaa.........................  1    1  Mm1
......................................................................Aggacggagaacugauaa.........................  1    1  Mm1
......................................................................uggacggagaacugaCaa.........................  1    1  Mm1
......................................................................uggacggagaacuAauaa.........................  1    1  Mm1
......................................................................uggacggagaacugauUa.........................  6    1  Mm1
......................................................................uggacggagaGcugauaa.........................  1    1  Mm1
......................................................................Nggacggagaacugauaa.........................  2    1  Mm1
......................................................................uggacggagaacugauaC.........................  2    1  Mm1
......................................................................uggacggagaacugauGa.........................  1    1  Mm1
......................................................................uggacUgagaacugauaa.........................  1    1  Mm1
......................................................................uggacgUagaacugauaa.........................  1    1  Mm1
......................................................................uggacggagaacugauaa.........................  995  0  Mm1
......................................................................uggacggagaacugauCa.........................  3    1  Mm1
......................................................................ugAacggagaacugauaa.........................  1    1  Mm1
......................................................................uggacggagaacugaAaa.........................  64   1  Mm1
......................................................................uAgacggagaacugauaa.........................  2    1  Mm1
```

```
                                  Star                                  Mature
cauuugugacaugagaaguaaguuugucucguguccucuucacuuuuccauccugucuguacucgagaacuggacggagaacugauaagggcauagggcagaccacaucaaca
......................................................................uggacggagaacugauaU.........................    29      1    Mm1
......................................................................uggacggagaacugauaG.........................    2       1    Mm1
......................................................................uggacggagaGcugauaag........................    19      1    Mm1
......................................................................uggacggagaacugGuaag........................    1       1    Mm1
......................................................................uggacAgagaacugauaag........................    2       1    Mm1
......................................................................uggacggagaacugUuaag........................    2       1    Mm1
......................................................................uggacggagaacugauaUg........................    14      1    Mm1
......................................................................Nggacggagaacugauaag........................    3       1    Mm1
......................................................................ugAacggagaacugauaag........................    1       1    Mm1
......................................................................uggacggagUacugauaag........................    2       1    Mm1
......................................................................uggacggagGacugauaag........................    6       1    Mm1
......................................................................uggacggagaCcugauaag........................    7       1    Mm1
......................................................................Aggacggagaacugauaag........................    3       1    Mm1
......................................................................uggacggagaacugauaaA........................    2940    1    Mm1
......................................................................uggacggagaacugauaaU........................    1409    1    Mm1
......................................................................uAgacggagaacugauaag........................    2       1    Mm1
......................................................................uggacggagaacugauaag........................    3971    0    Mm1
......................................................................uggacggagaacugauUag........................    5       1    Mm1
......................................................................uggacggagaacugauaGg........................    15      1    Mm1
......................................................................uggacggagaacugauaCg........................    16      1    Mm1
......................................................................uggacggagaacugauCag........................    2       1    Mm1
......................................................................ugCacggagaacugauaag........................    1       1    Mm1
......................................................................uggacggagaacugauGag........................    3       1    Mm1
......................................................................uggacggaAaacugauaag........................    2       1    Mm1
......................................................................uggaUggagaacugauaag........................    1       1    Mm1
......................................................................uggacgAagaacugauaag........................    1       1    Mm1
......................................................................uggacggagaacugauaaC........................    20      1    Mm1
......................................................................uggacgCagaacugauaag........................    1       1    Mm1
......................................................................uggUcggagaacugauaag........................    1       1    Mm1
......................................................................uggacggagaacugaAaagg.......................    1       1    Mm1
......................................................................uggacggagaacugaCaagg.......................    1       1    Mm1
......................................................................uggacggagaacugauaagU.......................    365     1    Mm1
......................................................................uggacCgagaacugauaagg.......................    2       1    Mm1
......................................................................uggaUggagaacugauaagg.......................    3       1    Mm1
......................................................................Aggacggagaacugauaagg.......................    53      1    Mm1
......................................................................uggacggUgaacugauaagg.......................    6       1    Mm1
......................................................................uggacggagaacugauaagg.......................    17362   0    Mm1
......................................................................uggacggagaacugauGagg.......................    40      1    Mm1
......................................................................uggacggagaacAgauaagg.......................    2       1    Mm1
......................................................................uUgacggagaacugauaagg.......................    5       1    Mm1
......................................................................Cggacggagaacugauaagg.......................    3       1    Mm1
......................................................................uggacggagUacugauaagg.......................    6       1    Mm1
......................................................................uggacAgagaacugauaagg.......................    5       1    Mm1
......................................................................uggGcggagaacugauaagg.......................    4       1    Mm1
......................................................................uggaGggagaacugauaagg.......................    1       1    Mm1
......................................................................uggUcggagaacugauaagg.......................    6       1    Mm1
......................................................................uggacggagaacugauaaUg.......................    1       1    Mm1
......................................................................uggacggCgaacugauaagg.......................    1       1    Mm1
......................................................................uggacggagaacugUuaagg.......................    2       1    Mm1
......................................................................uggacggagaacugaGaagg.......................    2       1    Mm1
......................................................................uggacggagaaUugauaagg.......................    14      1    Mm1
......................................................................uggacggagaacugauaUgg.......................    70      1    Mm1
......................................................................uggacggagaacugauaagA.......................    1788    1    Mm1
......................................................................uggacggGgaacugauaagg.......................    1       1    Mm1
......................................................................uggacggagCacugauaagg.......................    5       1    Mm1
......................................................................uCgacggagaacugauaagg.......................    2       1    Mm1
......................................................................ugCacggagaacugauaagg.......................    2       1    Mm1
......................................................................uggacggagaaGugauaagg.......................    1       1    Mm1
......................................................................uggacggagaCcugauaagg.......................    70      1    Mm1
......................................................................ugAacggagaacugauaagg.......................    1       1    Mm1
......................................................................uggaAggagaacugauaagg.......................    2       1    Mm1
......................................................................uggacggagaacugauaaAg.......................    24      1    Mm1
......................................................................uggacggagaacugauaGgg.......................    77      1    Mm1
......................................................................uggacggagaacugGuaagg.......................    3       1    Mm1
......................................................................uggacggagaacuUauaagg.......................    1       1    Mm1
......................................................................uggacggagaacCgauaagg.......................    4       1    Mm1
......................................................................uggacgAagaacugauaagg.......................    1       1    Mm1
......................................................................uAgacggagaacugauaagg.......................    5       1    Mm1
......................................................................uggacgUagaacugauaagg.......................    7       1    Mm1
......................................................................uggacNgagaacugauaagg.......................    3       1    Mm1
```

```
                                   Star                                Mature
cauuugugacaugagaaguaaguuugucucguguccucuucacuuuuccauccuguCuguacucgagaacuggacggagaacugauaagggcauagggcagaccacaucaaca
......................................................................Nggacggagaacugauaagg.......................  6      1  Mm1
......................................................................uggacggagaacugauaCgg.......................  71     1  Mm1
......................................................................uggacgCagaacugauaagg.......................  1      1  Mm1
......................................................................uggacggaUaacugauaagg.......................  1      1  Mm1
......................................................................uggacggagaacugauUagg.......................  29     1  Mm1
......................................................................uggacggagaacugauaagC.......................  3      1  Mm1
......................................................................uggacUgagaacugauaagg.......................  3      1  Mm1
......................................................................uggacggagaacugauCagg.......................  29     1  Mm1
......................................................................uggacggagaacuCauaagg.......................  2      1  Mm1
......................................................................uggacggagaGcugauaagg.......................  122    1  Mm1
......................................................................ugUacggagaacugauaagg.......................  3      1  Mm1
......................................................................uggacggagGacugauaagg.......................  8      1  Mm1
......................................................................uggacgCagaacugauaaggg......................  1      1  Mm1
......................................................................uggacggagUacugauaaggg......................  7      1  Mm1
......................................................................uAgacggagaacugauaaggg......................  22     1  Mm1
......................................................................uggacggagaacuAauaaggg......................  3      1  Mm1
......................................................................uggacggagaCcugauaaggg......................  33     1  Mm1
......................................................................uCgacggagaacugauaaggg......................  3      1  Mm1
......................................................................uggacNgagaacugauaaggg......................  1      1  Mm1
......................................................................uggacggagaGcugauaaggg......................  85     1  Mm1
......................................................................uggacggagaacugauaagUg......................  3      1  Mm1
......................................................................uggacggagaacugauaaUgg......................  3      1  Mm1
......................................................................uggacgUagaacugauaaggg......................  2      1  Mm1
......................................................................uggacUgagaacugauaaggg......................  3      1  Mm1
......................................................................uggacggagaacugauaUggg......................  41     1  Mm1
......................................................................uggacggagGacugauaaggg......................  3      1  Mm1
......................................................................uggacggUgaacugauaaggg......................  4      1  Mm1
......................................................................ugAacggagaacugauaaggg......................  3      1  Mm1
......................................................................uggacggagaacugauCaggg......................  29     1  Mm1
......................................................................uggacggagaacugauaGggg......................  82     1  Mm1
......................................................................uggacggagaacAgauaaggg......................  1      1  Mm1
......................................................................uggacggagaacugauGaggg......................  18     1  Mm1
......................................................................Nggacggagaacugauaaggg......................  6      1  Mm1
......................................................................uggacggagaaAugauaaggg......................  1      1  Mm1
......................................................................Cggacggagaacugauaaggg......................  2      1  Mm1
......................................................................uggacggagaaUugauaaggg......................  1      1  Mm1
......................................................................uggacggagaacugauaaggC......................  5      1  Mm1
......................................................................ugUacggagaacugauaaggg......................  1      1  Mm1
......................................................................uggacggagaacugauaaggA......................  3716   1  Mm1
......................................................................uggacggagaacugauUaggg......................  24     1  Mm1
......................................................................uggGcggagaacugauaaggg......................  3      1  Mm1
......................................................................uggacggagaacugUuaaggg......................  4      1  Mm1
......................................................................uggUcggagaacugauaaggg......................  3      1  Mm1
......................................................................uggacggagaacugGuaaggg......................  5      1  Mm1
......................................................................uggacggagaacugauaaggg......................  14223  0  Mm1
......................................................................uggacggagaacCgauaaggg......................  2      1  Mm1
......................................................................uggacggGgaacugauaaggg......................  2      1  Mm1
......................................................................uggacggagCacugauaaggg......................  2      1  Mm1
......................................................................uggaUggagaacugauaaggg......................  1      1  Mm1
......................................................................uggacggagaacugauaagCg......................  1      1  Mm1
......................................................................uggacggagaacugauaaggU......................  413    1  Mm1
......................................................................uUgacggagaacugauaaggg......................  1      1  Mm1
......................................................................uggacggCgaacugauaaggg......................  1      1  Mm1
......................................................................uggacAgagaacugauaaggg......................  3      1  Mm1
......................................................................uggacggagaacugauaCggg......................  59     1  Mm1
......................................................................uggacggaUaacugauaaggg......................  1      1  Mm1
......................................................................Aggacggagaacugauaaggg......................  31     1  Mm1
......................................................................uggacggagaacugauaagCgc.....................  8      1  Mm1
......................................................................uggacggagaacugauaaUggc.....................  17     1  Mm1
......................................................................uggacggagaacuUauaagggc.....................  8      1  Mm1
......................................................................uggacggGgaacugauaagggc.....................  32     1  Mm1
......................................................................uggacggagCacugauaagggc.....................  27     1  Mm1
......................................................................uggacggagaacugauaagUgc.....................  36     1  Mm1
......................................................................uggacgUagaacugauaagggc.....................  17     1  Mm1
......................................................................uggacggagaacugauaaggCc.....................  6      1  Mm1
......................................................................ugCacggagaacugauaagggc.....................  8      1  Mm1
......................................................................Aggacggagaacugauaagggc.....................  53     1  Mm1
......................................................................uggacggagaacugaCaagggc.....................  11     1  Mm1
......................................................................uggacggagUacugauaagggc.....................  49     1  Mm1
......................................................................uggacggagaaAugauaagggc.....................  6      1  Mm1
```

```
                                  Star                                  Mature
cauuugugacaugagaaguaaguuugucucguguccucuucacuuuuccauccugucuguacucgagaacuggacggagaacugauaagggcauagggcagaccacaucaaca
......................................................................uggaUggagaacugauaagggc.....................	20	1	Mm1
......................................................................uAgacggagaacugauaagggc.....................	47	1	Mm1
......................................................................uggacggagaCcugauaagggc.....................	88	1	Mm1
......................................................................uggacggagaacGgauaagggc.....................	3	1	Mm1
......................................................................Nggacggagaacugauaagggc.....................	48	1	Mm1
......................................................................uggacggagaacugauaaggAc.....................	18	1	Mm1
......................................................................uCgacggagaacugauaagggc.....................	10	1	Mm1
......................................................................uggacgNagaacugauaagggc.....................	3	1	Mm1
......................................................................uggacggaUaacugauaagggc.....................	19	1	Mm1
......................................................................uggacggaCaacugauaagggc.....................	6	1	Mm1
......................................................................uggaAggagaacugauaagggc.....................	8	1	Mm1
......................................................................uggacggagaacugauaUgggc.....................	321	1	Mm1
......................................................................uNgacggagaacugauaagggc.....................	1	1	Mm1
......................................................................uggacggagaacugauaaAggc.....................	21	1	Mm1
......................................................................uggacggagaGcugauaagggc.....................	209	1	Mm1
......................................................................uggacggaAaacugauaagggc.....................	6	1	Mm1
......................................................................uggacggagaacugUuaagggc.....................	9	1	Mm1
......................................................................uggacggagaacugauUagggc.....................	148	1	Mm1
......................................................................uggacggagaacugauaagggc.....................	99594	0	Mm1
......................................................................uggacggagaacugauaCgggc.....................	394	1	Mm1
......................................................................uggacggagaacugGuaagggc.....................	9	1	Mm1
......................................................................ugUacggagaacugauaagggc.....................	10	1	Mm1
......................................................................uggacggagaacugauaaggUc.....................	26	1	Mm1
......................................................................uggGcggagaacugauaagggc.....................	25	1	Mm1
......................................................................uggacggagaacugauaGgggc.....................	762	1	Mm1
......................................................................Cggacggagaacugauaagggc.....................	9	1	Mm1
......................................................................uUgacggagaacugauaagggc.....................	18	1	Mm1
......................................................................uggacggagaacugauGagggc.....................	154	1	Mm1
......................................................................uggacggagGacugauaagggc.....................	251	1	Mm1
......................................................................uggacgCagaacugauaagggc.....................	7	1	Mm1
......................................................................uggacUgagaacugauaagggc.....................	17	1	Mm1
......................................................................uggacggagaaGugauaagggc.....................	2	1	Mm1
......................................................................ugAacggagaacugauaagggc.....................	19	1	Mm1
......................................................................uggacggagaacuCauaagggc.....................	12	1	Mm1
......................................................................uggacggagaacugauaaCggc.....................	2	1	Mm1
......................................................................uggacggCgaacugauaagggc.....................	3	1	Mm1
......................................................................uggacggagaacugauCagggc.....................	209	1	Mm1
......................................................................uggCcggagaacugauaagggc.....................	24	1	Mm1
......................................................................uggacggagaacugauaagggG.....................	16	1	Mm1
......................................................................uggacggagaacugaAaagggc.....................	3	1	Mm1
......................................................................uggacggagaacugauNagggc.....................	1	1	Mm1
......................................................................uggacgAagaacugauaagggc.....................	8	1	Mm1
......................................................................uggacggagaaUugauaagggc.....................	20	1	Mm1
......................................................................uggacggagaacAgauaagggc.....................	5	1	Mm1
......................................................................uggUcggagaacugauaagggc.....................	13	1	Mm1
......................................................................uggaGggagaacugauaagggc.....................	1	1	Mm1
......................................................................uggacggagaacuAauaagggc.....................	18	1	Mm1
......................................................................uggacggagaacugauaagggU.....................	821	1	Mm1
......................................................................uggacggagaacCgauaagggc.....................	10	1	Mm1
......................................................................uggacNgagaacugauaagggc.....................	11	1	Mm1
......................................................................uggacggagaacugauaagggA.....................	4855	1	Mm1
......................................................................uggacggagaacugauaagAgc.....................	24	1	Mm1
......................................................................uggacAgagaacugauaagggc.....................	10	1	Mm1
......................................................................uggacggUgaacugauaagggc.....................	47	1	Mm1
......................................................................uggacggagaacugCuaagggc.....................	3	1	Mm1
......................................................................Gggacggagaacugauaagggca....................	1	1	Mm1
......................................................................uggacggagaacugauaCgggca....................	55	1	Mm1
......................................................................uggacUgagaacugauaagggca....................	2	1	Mm1
......................................................................uggUcggagaacugauaagggca....................	4	1	Mm1
......................................................................uggacggagCacugauaagggca....................	5	1	Mm1
......................................................................uggacggagaaGugauaagggca....................	1	1	Mm1
......................................................................uggacggagaacCgauaagggca....................	5	1	Mm1
......................................................................uggacggagGacugauaagggca....................	22	1	Mm1
......................................................................uggacggagaacugauaagggUa....................	42	1	Mm1
......................................................................uggacAgagaacugauaagggca....................	1	1	Mm1
......................................................................uggCcggagaacugauaagggca....................	2	1	Mm1
......................................................................uggacggagaacugauaaggCca....................	4	1	Mm1
......................................................................uggacggagUacugauaagggca....................	4	1	Mm1
......................................................................uggacggagaacugCuaagggca....................	1	1	Mm1
......................................................................uggacggagaacugauaaUggca....................	1	1	Mm1
```

Star Mature

cauuugugacaugagaaguaaguuugucucguguccucuucacuuuuccauccugucuguacucgagaacuggacggagaacugauaagggcauagggcagaccacaucaaca

....uggacggagaUcugauaagggca....................	1469	1	Mm1
....uggacggUgaacugauaagggca....................	2	1	Mm1
....uggacggagaacugauaagCgca....................	1	1	Mm1
....uggacggagaacuAauaagggca....................	3	1	Mm1
....uggacggagaacugauCagggca....................	20	1	Mm1
....uAgacggagaacugauaagggca....................	3	1	Mm1
....uggacgUagaacugauaagggca....................	2	1	Mm1
....Aggacggagaacugauaagggca....................	2	1	Mm1
....uggGcggagaacugauaagggca....................	2	1	Mm1
....ugUacggagaacugauaagggca....................	2	1	Mm1
....ugAacggagaacugauaagggca....................	4	1	Mm1
....uggacggagaacugauUagggca....................	16	1	Mm1
....uggacggagaacugauaagggAa....................	339	1	Mm1
....Cggacggagaacugauaagggca....................	3	1	Mm1
....uggacggCgaacugauaagggca....................	2	1	Mm1
....uggacggagaaUugauaagggca....................	2	1	Mm1
....uggacggGgaacugauaagggca....................	4	1	Mm1
....uggacggagaacugGuaagggca....................	1	1	Mm1
....uggacggagaacugauaagggGa....................	2	1	Mm1
....uggacggagaacugauaaCggca....................	1	1	Mm1
....uggacggagaacugUuaagggca....................	3	1	Mm1
....ugCacggagaacugauaagggca....................	2	1	Mm1
....uggacNgagaacugauaagggca....................	1	1	Mm1
....uUgacggagaacugauaagggca....................	3	1	Mm1
....uggacggagaacugauaaggAca....................	2	1	Mm1
....uggaUggagaacugauaagggca....................	1	1	Mm1
....uggacggagaacugauaagUgca....................	3	1	Mm1
....Nggacggagaacugauaagggca....................	6	1	Mm1
....uggacggagaacugauaagAgca....................	1	1	Mm1
....uggacggaUaacugauaagggca....................	1	1	Mm1
....uggacggagaacugauaGgggca....................	57	1	Mm1
....uggacggagaacugauaaAggca....................	1	1	Mm1
....uggacggagaacugauaagggcC....................	44	1	Mm1
....uggacgAagaacugauaagggca....................	1	1	Mm1
....uggacggagaacugauaagggca....................	11464	0	Mm1
....uggacgNagaacugauaagggca....................	1	1	Mm1
....uggacggagaacugauaagggcG....................	66	1	Mm1
....uggacggagaacugauaaggUca....................	4	1	Mm1
....uggacggagaacAgauaagggca....................	1	1	Mm1
....uggacggagaCcugauaagggca....................	12	1	Mm1
....uggacggagaacugauaUgggca....................	32	1	Mm1
....uggacggagaGcugauaagggca....................	20	1	Mm1
....uggacggagaacugauGagggca....................	16	1	Mm1
....uggacggagaacugauaagggUau...................	39	1	Mm1
....uggacggagaacugauaagggAau...................	202	1	Mm1
....uggacggagaacuAauaagggcau...................	1	1	Mm1
....uggacggagaacugauaagggcCu...................	5	1	Mm1
....uggacggagaUcugauaagggcau...................	194	1	Mm1
....uggacggagaacugauaagggcaC...................	14	1	Mm1
....uggacggagaacugauaagggcaA...................	30	1	Mm1
....uggacggagaacugauUagggcau...................	2	1	Mm1
....Nggacggagaacugauaagggcau...................	1	1	Mm1
....uggacggagaacugauaUgggcau...................	3	1	Mm1
....uggacggagaacugauGagggcau...................	2	1	Mm1
....uggacggagaacugauaagggcau...................	951	0	Mm1
....uggacggagaacugauCagggcau...................	4	1	Mm1
....uggaUggagaacugauaagggcau...................	1	1	Mm1
....uggacggagaacugauaagggcaG...................	18	1	Mm1
....uggacggagaacugauaGgggcau...................	4	1	Mm1
....uggacggagaacugaCaagggcau...................	1	1	Mm1
....uggacggagaGcugauaagggcau...................	2	1	Mm1
....Cggacggagaacugauaagggcau...................	2	1	Mm1
....uggacggagaacugauaagggcGu...................	7	1	Mm1
....uggacggagaacugauaCgggcau...................	2	1	Mm1
....uggacggagaacugauaagggcUua..................	5	1	Mm1
....uggacggagaacugauaagggcauC..................	5	1	Mm1
....uggacggagaacugauaagggcauU..................	101	1	Mm1
....uggacggagaacugauaagggcaua..................	2	0	Mm1
....uggacggagaacugGuaagggcaua..................	1	1	Mm1
....uggacggagaacugauaagggcauaU.................	2	1	Mm1

```
                                  Star                                  Mature
cauuugugacaugagaaguaaguuugucucguguccucuucacuuuuccauccugucuguacucgagaacuggacggagaacugauaagggcauagggcagaccacaucaaca
......................................................................uggacggagaUcugauaagggcauag.................	6	1	Mm1
.......................................................................ggacggagaacugauaaA........................	9	1	Mm1
.......................................................................ggacggagaacugauaag........................	9	0	Mm1
.......................................................................ggacggagaaAugauaag........................	1	1	Mm1
.......................................................................ggacggagaacugauaagg.......................	27	0	Mm1
.......................................................................ggacggagCacugauaagg.......................	1	1	Mm1
.......................................................................ggacggagaacugauaagU.......................	5	1	Mm1
.......................................................................ggacggagaacugauaaggA......................	7	1	Mm1
.......................................................................ggGcggagaacugauaaggg......................	1	1	Mm1
.......................................................................Ugacggagaacugauaaggg......................	1	1	Mm1
.......................................................................ggacggagaacugauaaggC......................	1	1	Mm1
.......................................................................ggacggagaacugauaaggg......................	43	0	Mm1
.......................................................................ggacggagaCcugauaagggc.....................	1	1	Mm1
.......................................................................ggacggaAaacugauaagggc.....................	1	1	Mm1
.......................................................................ggacggagaacGgauaagggc.....................	1	1	Mm1
.......................................................................ggacggagaacugauaagggU.....................	2	1	Mm1
.......................................................................ggacggagaacugauaaUggc.....................	1	1	Mm1
.......................................................................ggacggagGacugauaagggc.....................	2	1	Mm1
.......................................................................ggacggagaacugauaagggc.....................	178	0	Mm1
.......................................................................ggaAggagaacugauaagggc.....................	1	1	Mm1
.......................................................................ggacggagaacugauaagggA.....................	7	1	Mm1
.......................................................................ggGcggagaacugauaagggc.....................	2	1	Mm1
.......................................................................ggacggagCacugauaagggc.....................	1	1	Mm1
.......................................................................ggacggagaaAugauaagggc.....................	1	1	Mm1
.......................................................................ggacggagaacugauaaggAc.....................	1	1	Mm1
.......................................................................ggacggagaaGugauaagggc.....................	2	1	Mm1
.......................................................................Ugacggagaacugauaagggc.....................	4	1	Mm1
.......................................................................ggacggagaacugauaagggca....................	17	0	Mm1
.......................................................................ggacggagaacugauaaggCca....................	1	1	Mm1
.......................................................................ggacggagaUcugauaagggca....................	10	1	Mm1
.......................................................................ggacggagaacugauaagggAau...................	3	1	Mm1
..........................................................................Uggagaacugauaagggc.....................	1	1	Mm1

..................................ccucuucacuuuuccauccugu.........................................................	14	0	Fm1
..................................ccucuucacuuuuccauccugG.........................................................	1	1	Fm1
..................................ccucuucacuuuuccauccuguU........................................................	3	1	Fm1
..................................ccucuucacuuuuccauccuguc........................................................	2	0	Fm1
..................................ccucuucacuuuuccauccugucugCacuc.................................................	1	1	Fm1
..................................ccucuucacuuuuccauccugucuguacuc.................................................	6	0	Fm1
..................................ccucuucacuuuuccauccugucuguacucgagaa............................................	1	0	Fm1
..................................ccucuucacuuuuccauccugucuguacucgagaacG..........................................	1	1	Fm1
.............................................uuccauccugucuguacucgaga.............................................	2	0	Fm1
...............................................................cgagaacuggacggagaacugauaagggc.....................	6	0	Fm1
...................................................................aUcuggacggagaacugauaagggc.....................	4	1	Fm1
....................................................................acuggacggagaacugauaaU........................	4	1	Fm1
....................................................................acuggacggagaacugauaagg.......................	2	0	Fm1
....................................................................acuggacggagaacugauaaggA......................	2	1	Fm1
....................................................................acuggacggagaacugauaagggc.....................	3	0	Fm1
.....................................................................cuggacggagaacugaua..........................	2	0	Fm1
.....................................................................cuggacggagGacugauaa.........................	1	1	Fm1
.....................................................................cuggacggagaacugauaa.........................	6	0	Fm1
.....................................................................cuggacggagaacugauCa.........................	1	1	Fm1
.....................................................................cuggacggagaacugaAaa.........................	5	1	Fm1
.....................................................................cuggacggagaacugauaag........................	36	0	Fm1
.....................................................................cuggacggagaacugauaaA........................	14	1	Fm1
.....................................................................cAggacggagaacugauaag........................	1	1	Fm1
.....................................................................cuggacggagaacugauaaU........................	3	1	Fm1
.....................................................................cuggacggagaacugauCagg.......................	1	1	Fm1
.....................................................................cuggacggagaacugauaagg.......................	165	0	Fm1
.....................................................................cuggaNggagaacugauaagg.......................	1	1	Fm1
.....................................................................cuggacggagaacugauaagU.......................	1	1	Fm1
.....................................................................cuggacggagaacugauaagA.......................	18	1	Fm1
.....................................................................Nuggacggagaacugauaagg.......................	1	1	Fm1
.....................................................................cuggacggaUaacugauaagg.......................	1	1	Fm1
.....................................................................cuggacggagaacAgauaagg.......................	1	1	Fm1
.....................................................................cuggacggagaacugauaaggU......................	32	1	Fm1
.....................................................................cuggacggagaacugauaaggC......................	5	1	Fm1
.....................................................................cuggacggagCacugauaaggg......................	1	1	Fm1
.....................................................................cuggacggagaacugauaaggg......................	169	0	Fm1
.....................................................................cuggacggagaacugauaaggA......................	15	1	Fm1
```

```
                                  Star                                   Mature
cauuugugacaugagaaguaaguuugucucguguccucuucacuuuuccauccugucuguacucgagaacuggacggagaacugauaagggcauagggcagaccacaucaaca
.....................................................................cuggacggagUacugauaaggg......................   1      1   Fm1
.....................................................................cuggacggagaacugauGaggg......................   2      1   Fm1
.....................................................................cuggacUgagaacugauaaggg......................   1      1   Fm1
.....................................................................cuggacggagaacugaGaaggg......................   1      1   Fm1
.....................................................................cuggacggagaacugaAaagggc.....................   1      1   Fm1
.....................................................................cuggacggagaacugauCagggc.....................   1      1   Fm1
.....................................................................cuggacggagGacugauaagggc.....................   4      1   Fm1
.....................................................................cuggacggagaacugauaGgggc.....................   9      1   Fm1
.....................................................................cuggacggagaacugauaagggc.....................   422    0   Fm1
.....................................................................cuggacAgagaacugauaagggc.....................   1      1   Fm1
.....................................................................cuggacggagaacugauaagggA.....................   37     1   Fm1
.....................................................................cuggacggagaCcugauaagggc.....................   1      1   Fm1
.....................................................................cGggacggagaacugauaagggc.....................   1      1   Fm1
.....................................................................cuggacggagaaUugauaagggc.....................   1      1   Fm1
.....................................................................cuggacggagaacugauUagggc.....................   1      1   Fm1
.....................................................................cuggacUgagaacugauaagggc.....................   1      1   Fm1
.....................................................................cuggacggagaacugauGagggc.....................   1      1   Fm1
.....................................................................cuggacggUgaacugauaagggc.....................   1      1   Fm1
.....................................................................cuggacggagaacugauaagggU.....................   4      1   Fm1
.....................................................................cuggacggagaacugaCaagggc.....................   4      1   Fm1
.....................................................................cuggacggGgaacugauaagggc.....................   2      1   Fm1
.....................................................................Uuggacggagaacugauaagggc.....................   1      1   Fm1
.....................................................................Nuggacggagaacugauaagggc.....................   1      1   Fm1
.....................................................................cuggacggagUacugauaagggc.....................   2      1   Fm1
.....................................................................cuggacggagaCcugauaagggca....................   1      1   Fm1
.....................................................................cuggacggagaacugauaagggca....................   38     0   Fm1
.....................................................................cuggacggagaacugauaagggcG....................   1      1   Fm1
.....................................................................cuggacggagaacugauaagggAa....................   2      1   Fm1
.....................................................................cuggacggagaacugauaagggcau...................   3      0   Fm1
......................................................................uggacggagaacugauaa.........................   2454   0   Fm1
......................................................................uggacggagaacugauaG.........................   14     1   Fm1
......................................................................uggacggagaacugauaC.........................   15     1   Fm1
......................................................................uggacggagaacugauCa.........................   3      1   Fm1
......................................................................ugAacggagaacugauaa.........................   1      1   Fm1
......................................................................uggacggagaacugaAaa.........................   215    1   Fm1
......................................................................uggacggagaGcugauaa.........................   10     1   Fm1
......................................................................uggacggagaacugauaU.........................   88     1   Fm1
......................................................................uAgacggagaacugauaa.........................   2      1   Fm1
......................................................................uggacggagUacugauaa.........................   1      1   Fm1
......................................................................uggacggagaacugUuaa.........................   1      1   Fm1
......................................................................uggacggagaacugaGaa.........................   6      1   Fm1
......................................................................uggacggagaCcugauaa.........................   1      1   Fm1
......................................................................uggacggagaacugauGa.........................   1      1   Fm1
......................................................................Nggacggagaacugauaa.........................   1      1   Fm1
......................................................................uggacggagaacugCuaa.........................   1      1   Fm1
......................................................................uggacggUgaacugauaa.........................   2      1   Fm1
......................................................................uggGcggagaacugauaa.........................   1      1   Fm1
......................................................................uggacggagaacugauUa.........................   9      1   Fm1
......................................................................uggacggagaacGgauaag........................   1      1   Fm1
......................................................................Nggacggagaacugauaag........................   10     1   Fm1
......................................................................Aggacggagaacugauaag........................   3      1   Fm1
......................................................................uggGcggagaacugauaag........................   8      1   Fm1
......................................................................uggUcggagaacugauaag........................   5      1   Fm1
......................................................................uggacggagUacugauaag........................   3      1   Fm1
......................................................................uggacggagaacugauCag........................   39     1   Fm1
......................................................................uggaUggagaacugauaag........................   4      1   Fm1
......................................................................ugUacggagaacugauaag........................   1      1   Fm1
......................................................................uggacggagaacugauUag........................   24     1   Fm1
......................................................................uggacggagaacugauaaA........................   6927   1   Fm1
......................................................................uggacggagaacugauGag........................   21     1   Fm1
......................................................................uUgacggagaacugauaag........................   1      1   Fm1
......................................................................uggacggagaacuCauaag........................   2      1   Fm1
......................................................................uggacggagGacugauaag........................   9      1   Fm1
......................................................................uggacggagaacugauaGg........................   72     1   Fm1
......................................................................uggacggagaacCgauaag........................   4      1   Fm1
......................................................................uAgacggagaacugauaag........................   10     1   Fm1
......................................................................uNgacggagaacugauaag........................   1      1   Fm1
......................................................................uggacggagaacugUuaag........................   6      1   Fm1
......................................................................uggacggagaacugauaCg........................   70     1   Fm1
......................................................................uggacggGgaacugauaag........................   4      1   Fm1
```

```
                                  Star                                  Mature
cauuugugacaugagaaguaaguuugucucguguccucuucacuuuuccauccugucuguacucgagaacuggacggagaacugauaagggcauagggcagaccacaucaaca
......................................................................uggacUgagaacugauaag........................   5       1   Fm1
......................................................................uggacggUgaacugauaag........................   4       1   Fm1
......................................................................uggaGggagaacugauaag........................   1       1   Fm1
......................................................................uggacAgagaacugauaag........................   5       1   Fm1
......................................................................uggacgAagaacugauaag........................   3       1   Fm1
......................................................................uggacCgagaacugauaag........................   1       1   Fm1
......................................................................uggacgUagaacugauaag........................   5       1   Fm1
......................................................................ugAacggagaacugauaag........................   5       1   Fm1
......................................................................uggacggagaacugauaUg........................   63      1   Fm1
......................................................................uggCcggagaacugauaag........................   1       1   Fm1
......................................................................uggacggagaacugauaaU........................   4300    1   Fm1
......................................................................uggacggagaaAugauaag........................   1       1   Fm1
......................................................................ugCacggagaacugauaag........................   2       1   Fm1
......................................................................uggacggagaacAgauaag........................   1       1   Fm1
......................................................................uggacggagaacugCuaag........................   1       1   Fm1
......................................................................uggacggaUaacugauaag........................   4       1   Fm1
......................................................................uggacggagaCcugauaag........................   24      1   Fm1
......................................................................uggacggagaacuAauaag........................   2       1   Fm1
......................................................................uggacggagaacuUauaag........................   3       1   Fm1
......................................................................uggaAggagaacugauaag........................   1       1   Fm1
......................................................................Cggacggagaacugauaag........................   2       1   Fm1
......................................................................uggacggagaacugaCaag........................   2       1   Fm1
......................................................................uggacgCagaacugauaag........................   1       1   Fm1
......................................................................uggacggagCacugauaag........................   2       1   Fm1
......................................................................uggacggagaacugauaaC........................   30      1   Fm1
......................................................................uggacggagaacugauaag........................   17744   0   Fm1
......................................................................uggacggagaacugaAaag........................   19      1   Fm1
......................................................................uggacggagaaUugauaag........................   8       1   Fm1
......................................................................uggacggaAaacugauaag........................   3       1   Fm1
......................................................................uggacggagaGcugauaag........................   31      1   Fm1
......................................................................uggacNgagaacugauaag........................   4       1   Fm1
......................................................................uggUcggagaacugauaagg.......................   8       1   Fm1
......................................................................uggacggagaacuCauaagg.......................   4       1   Fm1
......................................................................uCgacggagaacugauaagg.......................   4       1   Fm1
......................................................................uggacggagaacugaCaagg.......................   2       1   Fm1
......................................................................uggacggagCacugauaagg.......................   6       1   Fm1
......................................................................uggacggGgaacugauaagg.......................   8       1   Fm1
......................................................................uggCcggagaacugauaagg.......................   9       1   Fm1
......................................................................uggacggaCaacugauaagg.......................   1       1   Fm1
......................................................................uAgacggagaacugauaagg.......................   27      1   Fm1
......................................................................uggacggagGacugauaagg.......................   51      1   Fm1
......................................................................uggacggCgaacugauaagg.......................   4       1   Fm1
......................................................................uggacggagaacugauGagg.......................   63      1   Fm1
......................................................................uggacggagaacugauaUgg.......................   165     1   Fm1
......................................................................uggacggagaacuAauaagg.......................   9       1   Fm1
......................................................................uggacggagaacugauaagg.......................   46362   0   Fm1
......................................................................uggaAggagaacugauaagg.......................   1       1   Fm1
......................................................................uggacUgagaacugauaagg.......................   14      1   Fm1
......................................................................uggacggagaaGugauaagg.......................   1       1   Fm1
......................................................................uggacggagaacAgauaagg.......................   2       1   Fm1
......................................................................uggacggagaacugauaCgg.......................   177     1   Fm1
......................................................................uggacgUagaacugauaagg.......................   8       1   Fm1
......................................................................uggacggagaacugauaaCg.......................   4       1   Fm1
......................................................................uggacCgagaacugauaagg.......................   2       1   Fm1
......................................................................uggacggaAaacugauaagg.......................   2       1   Fm1
......................................................................uggacAgagaacugauaagg.......................   5       1   Fm1
......................................................................uggacgNagaacugauaagg.......................   2       1   Fm1
......................................................................uggacgCagaacugauaagg.......................   2       1   Fm1
......................................................................uggacggagaacugUuaagg.......................   9       1   Fm1
......................................................................ugCacggagaacugauaagg.......................   1       1   Fm1
......................................................................uggacggagaacugGuaagg.......................   7       1   Fm1
......................................................................uggacggagaacugauCagg.......................   122     1   Fm1
......................................................................uggacggaUaacugauaagg.......................   8       1   Fm1
......................................................................uUgacggagaacugauaagg.......................   8       1   Fm1
......................................................................uggacggagaacugauaagC.......................   31      1   Fm1
......................................................................Aggacggagaacugauaagg.......................   17      1   Fm1
......................................................................ugAacggagaacugauaagg.......................   9       1   Fm1
......................................................................Cggacggagaacugauaagg.......................   9       1   Fm1
......................................................................uNgacggagaacugauaagg.......................   2       1   Fm1
......................................................................uggacggagaacugCuaagg.......................   2       1   Fm1
```

Star Mature

cauuugugacaugagaaguaaguuugucucguguccucuucacuuuuccauccuguucuguacucgagaacuggacggagaacugauaagggcauagggcagaccacaucaaca			
....uggacNgagaacugauaagg.......................	2	1	Fm1
....uggacggagaaUugauaagg.......................	8	1	Fm1
....uggacggagaCcugauaagg.......................	71	1	Fm1
....uggacggagaacugauUagg.......................	70	1	Fm1
....Nggacggagaacugauaagg.......................	17	1	Fm1
....uggGcggagaacugauaagg.......................	16	1	Fm1
....uggacggUgaacugauaagg.......................	18	1	Fm1
....uggacggagaacugauaGgg.......................	199	1	Fm1
....uggacggagaacuUauaagg.......................	1	1	Fm1
....uggacggagaacugauaagU.......................	1110	1	Fm1
....uggaUggagaacugauaagg.......................	9	1	Fm1
....uggacggagaacCgauaagg.......................	3	1	Fm1
....uggacggagaGcugauaagg.......................	149	1	Fm1
....uggacggagUacugauaagg.......................	17	1	Fm1
....ugUacggagaacugauaagg.......................	5	1	Fm1
....uggacggagaacugaAaagg.......................	2	1	Fm1
....uggacggagaacugauaaUg.......................	3	1	Fm1
....uggacgAagaacugauaagg.......................	4	1	Fm1
....uggacggagaacugauaagA.......................	4462	1	Fm1
....uggacggagaacugauaaAg.......................	266	1	Fm1
....uggacgAagaacugauaaggg......................	8	1	Fm1
....uggacggagaacugauaaggC......................	348	1	Fm1
....uggacgUagaacugauaaggg......................	16	1	Fm1
....uggacggaUaacugauaaggg......................	14	1	Fm1
....uggaUggagaacugauaaggg......................	13	1	Fm1
....uggacggagUacugauaaggg......................	19	1	Fm1
....uggacggagCacugauaaggg......................	8	1	Fm1
....uggacAgagaacugauaaggg......................	8	1	Fm1
....ugUacggagaacugauaaggg......................	3	1	Fm1
....uggacggagaacugauaaggg......................	58344	0	Fm1
....uggGcggagaacugauaaggg......................	14	1	Fm1
....uggacggagaacuAauaaggg......................	13	1	Fm1
....ugNacggagaacugauaaggg......................	1	1	Fm1
....uggacggagaacugauaCggg......................	225	1	Fm1
....uggacggagaacAgauaaggg......................	4	1	Fm1
....uggacggagaacugauaaggU......................	1894	1	Fm1
....uggacggagaacugauaUggg......................	223	1	Fm1
....uCgacggagaacugauaaggg......................	5	1	Fm1
....uggacggagaacCgauaaggg......................	12	1	Fm1
....uggUcggagaacugauaaggg......................	6	1	Fm1
....uggacgNagaacugauaaggg......................	5	1	Fm1
....uggacCgagaacugauaaggg......................	4	1	Fm1
....uggacggagaacugauaaggA......................	11343	1	Fm1
....uggacggagaacugaGaaggg......................	2	1	Fm1
....uggaAggagaacugauaaggg......................	1	1	Fm1
....uggacggagGacugauaaggg......................	59	1	Fm1
....uggacggagaacugauUaggg......................	93	1	Fm1
....uggacggagaaUugauaaggg......................	20	1	Fm1
....uggacggaAaacugauaaggg......................	2	1	Fm1
....uggacgCagaacugauaaggg......................	3	1	Fm1
....uggacggagaacugauGaggg......................	97	1	Fm1
....uggacggaCaacugauaaggg......................	3	1	Fm1
....uggacggagaacugauaagUg......................	14	1	Fm1
....uggCcggagaacugauaaggg......................	10	1	Fm1
....uggaGggagaacugauaaggg......................	3	1	Fm1
....uggacNgagaacugauaaggg......................	11	1	Fm1
....uggacggagaCcugauaaggg......................	72	1	Fm1
....uggacUgagaacugauaaggg......................	15	1	Fm1
....ugCacggagaacugauaaggg......................	1	1	Fm1
....uggacggGgaacugauaaggg......................	18	1	Fm1
....ugAacggagaacugauaaggg......................	12	1	Fm1
....uggacggagaacugauaaUgg......................	8	1	Fm1
....uggacggagaacugCuaaggg......................	4	1	Fm1
....uggacggagaacuUauaaggg......................	4	1	Fm1
....uUgacggagaacugauaaggg......................	14	1	Fm1
....uAgacggagaacugauaaggg......................	30	1	Fm1
....uggacggagaacGgauaaggg......................	1	1	Fm1
....uggacggCgaacugauaaggg......................	5	1	Fm1
....uggacggagaacugGuaaggg......................	13	1	Fm1
....uggacggagaacugUuaaggg......................	11	1	Fm1

```
                                  Star                                  Mature
cauuugugacaugagaaguaaguuugucucguguccucuucacuuuuccauccuguCuguacucgagaacuggacggagaacugauaagggcauagggcagaccacaucaaca
......................................................................uggacggagaacugauaGggg......................  524     1  Fm1
......................................................................uggacggagaacugauaagCg......................  5       1  Fm1
......................................................................uggacggagaacuCauaaggg......................  5       1  Fm1
......................................................................uggacggagaacugaCaaggg......................  2       1  Fm1
......................................................................uggacggagaacugaAaaggg......................  2       1  Fm1
......................................................................Gggacggagaacugauaaggg......................  1       1  Fm1
......................................................................Cggacggagaacugauaaggg......................  9       1  Fm1
......................................................................uggacggagaGcugauaaggg......................  136     1  Fm1
......................................................................uggacggUgaacugauaaggg......................  20      1  Fm1
......................................................................uggacggagaacugauCaggg......................  154     1  Fm1
......................................................................uggacggagaacugauaagAg......................  22      1  Fm1
......................................................................Nggacggagaacugauaaggg......................  26      1  Fm1
......................................................................uggacggagaacugauaaAgg......................  6       1  Fm1
......................................................................Aggacggagaacugauaaggg......................  24      1  Fm1
......................................................................Nggacggagaacugauaagggc.....................  440     1  Fm1
......................................................................uggacggagaacugauaaAggc.....................  61      1  Fm1
......................................................................uggacggUgaacugauaagggc.....................  431     1  Fm1
......................................................................uggacggagaacuUauaagggc.....................  78      1  Fm1
......................................................................uggacggagaacCgauaagggc.....................  121     1  Fm1
......................................................................Aggacggagaacugauaagggc.....................  153     1  Fm1
......................................................................uggacggagaacuCauaagggc.....................  49      1  Fm1
......................................................................uggacggagaacugauaagggA.....................  21574   1  Fm1
......................................................................uggacggagaCcugauaagggc.....................  672     1  Fm1
......................................................................uUgacggagaacugauaagggc.....................  193     1  Fm1
......................................................................uggacggagUacugauaagggc.....................  282     1  Fm1
......................................................................uggacggagaacugauaGgggc.....................  8811    1  Fm1
......................................................................uggacgUagaacugauaagggc.....................  178     1  Fm1
......................................................................uggacggagCacugauaagggc.....................  169     1  Fm1
......................................................................uggacggagaacugauaNgggc.....................  2       1  Fm1
......................................................................uggacggagaaUugauaagggc.....................  284     1  Fm1
......................................................................uggacggagaacugCuaagggc.....................  25      1  Fm1
......................................................................uggacggagaacugauaagggc.....................  846342  0  Fm1
......................................................................uggacggagaGcugauaagggc.....................  1253    1  Fm1
......................................................................uNgacggagaacugauaagggc.....................  7       1  Fm1
......................................................................ugNacggagaacugauaagggc.....................  4       1  Fm1
......................................................................ugAacggagaacugauaagggc.....................  183     1  Fm1
......................................................................uggGcggagaacugauaagggc.....................  170     1  Fm1
......................................................................uggacgNagaacugauaagggc.....................  46      1  Fm1
......................................................................uggacggagaacugauaagCgc.....................  50      1  Fm1
......................................................................uggacggagaacugauCagggc.....................  1755    1  Fm1
......................................................................ugCacggagaacugauaagggc.....................  65      1  Fm1
......................................................................uggacgCagaacugauaagggc.....................  47      1  Fm1
......................................................................uggacgAagaacugauaagggc.....................  98      1  Fm1
......................................................................uggacggagaacAgauaagggc.....................  31      1  Fm1
......................................................................uggacggagaacugauaagggU.....................  7010    1  Fm1
......................................................................uggaAggagaacugauaagggc.....................  66      1  Fm1
......................................................................uggacggagaacuAauaagggc.....................  170     1  Fm1
......................................................................uggacUgagaacugauaagggc.....................  145     1  Fm1
......................................................................uggacggagaacugauaagggG.....................  874     1  Fm1
......................................................................uggUcggagaacugauaagggc.....................  135     1  Fm1
......................................................................uggacggagaacugauaagAgc.....................  127     1  Fm1
......................................................................uggacNgagaacugauaagggc.....................  135     1  Fm1
......................................................................uggacAgagaacugauaagggc.....................  137     1  Fm1
......................................................................uggacggagaacugUuaagggc.....................  110     1  Fm1
......................................................................uggacggagaacugauaaggAc.....................  313     1  Fm1
......................................................................uggacggagaacugGuaagggc.....................  99      1  Fm1
......................................................................uggacggagaacugauaagUgc.....................  175     1  Fm1
......................................................................uggacggagaacugaGaagggc.....................  21      1  Fm1
......................................................................uggacggaCaacugauaagggc.....................  44      1  Fm1
......................................................................uggacggagaacugauaaggUc.....................  223     1  Fm1
......................................................................uggacggagaacugauaaggCc.....................  116     1  Fm1
......................................................................uggacggagGacugauaagggc.....................  1684    1  Fm1
......................................................................uggacggagaacugauUagggc.....................  1143    1  Fm1
......................................................................uggacggCgaacugauaagggc.....................  86      1  Fm1
......................................................................uggacggaUaacugauaagggc.....................  154     1  Fm1
......................................................................uggacggagaacugauaaCggc.....................  16      1  Fm1
......................................................................uAgacggagaacugauaagggc.....................  390     1  Fm1
......................................................................Gggacggagaacugauaagggc.....................  22      1  Fm1
......................................................................uggacggagaacugauGagggc.....................  1216    1  Fm1
......................................................................uggacggaAaacugauaagggc.....................  49      1  Fm1
```

Star **Mature**

```
cauuugugacaugagaaguaaguuugucucguguccucuucacuuuuccauccuguCuguacucgagaacuggacggagaacugauaagggcauagggcagaccacaucaaca
......................................................................uggCcggagaacugauaagggc.....................	174	1	Fm1
......................................................................Cggacggagaacugauaagggc.....................	102	1	Fm1
......................................................................uggacggagaacugaCaagggc.....................	80	1	Fm1
......................................................................ugUacggagaacugauaagggc.....................	131	1	Fm1
......................................................................uggacggagaacGgauaagggc.....................	33	1	Fm1
......................................................................uggacggagaaAugauaagggc.....................	22	1	Fm1
......................................................................uggaUggagaacugauaagggc.....................	194	1	Fm1
......................................................................uggacggGgaacugauaagggc.....................	194	1	Fm1
......................................................................uggaGggagaacugauaagggc.....................	38	1	Fm1
......................................................................uCgacggagaacugauaagggc.....................	73	1	Fm1
......................................................................uggacggagaacugaAaagggc.....................	18	1	Fm1
......................................................................uggacCgagaacugauaagggc.....................	34	1	Fm1
......................................................................uggacggagaaGugauaagggc.....................	18	1	Fm1
......................................................................uggacggagaacugauaaUggc.....................	128	1	Fm1
......................................................................uggacggagaacugauaUgggc.....................	2648	1	Fm1
......................................................................uggacggagaacugauaCgggc.....................	3205	1	Fm1
......................................................................uggacggagaacugauCagggca....................	121	1	Fm1
......................................................................uggacggagaacugauaagggUa....................	193	1	Fm1
......................................................................uggacggagaacugauaagAgca....................	9	1	Fm1
......................................................................uggacggagaacugUuaagggca....................	11	1	Fm1
......................................................................uggacggagaacCgauaagggca....................	18	1	Fm1
......................................................................uggacUgagaacugauaagggca....................	7	1	Fm1
......................................................................Nggacggagaacugauaagggca....................	28	1	Fm1
......................................................................uggacggagaacugauGagggca....................	91	1	Fm1
......................................................................uggacggCgaacugauaagggca....................	2	1	Fm1
......................................................................uggaAggagaacugauaagggca....................	4	1	Fm1
......................................................................uggacggagaacugauaaggCca....................	15	1	Fm1
......................................................................uggacggagaacugauaGggca....................	399	1	Fm1
......................................................................uggacggagaacugauaagggca....................	56469	0	Fm1
......................................................................uggacggagaaUugauaagggca....................	17	1	Fm1
......................................................................Aggacggagaacugauaagggca....................	9	1	Fm1
......................................................................uggacggGgaacugauaagggca....................	13	1	Fm1
......................................................................uggacggaCaacugauaagggca....................	2	1	Fm1
......................................................................uggacggagaacugauaagggGa....................	6	1	Fm1
......................................................................uggacggagaacugauUagggca....................	85	1	Fm1
......................................................................uggacggagaacuCauaagggca....................	2	1	Fm1
......................................................................uggacggagaacugauaagUgca....................	15	1	Fm1
......................................................................uggacggagCacugauaagggca....................	8	1	Fm1
......................................................................uggacggagaacuAauaagggca....................	13	1	Fm1
......................................................................ugNacggagaacugauaagggca....................	2	1	Fm1
......................................................................uggacggagaGcugauaagggca....................	78	1	Fm1
......................................................................uggacNgagaacugauaagggca....................	9	1	Fm1
......................................................................uggacggagaacugauaaCggca....................	2	1	Fm1
......................................................................uggacggagaCcugauaagggca....................	44	1	Fm1
......................................................................uggacggagaacugauaaAggca....................	7	1	Fm1
......................................................................uggacggagaacugGuaagggca....................	6	1	Fm1
......................................................................Gggacggagaacugauaagggca....................	2	1	Fm1
......................................................................uggGcggagaacugauaagggca....................	15	1	Fm1
......................................................................uggacggagaacugauaagCgca....................	4	1	Fm1
......................................................................uggacggagaacugauaagggAa....................	1120	1	Fm1
......................................................................uggacgAagaacugauaagggca....................	6	1	Fm1
......................................................................uggacggagaacugauaaggUca....................	22	1	Fm1
......................................................................uggacggagGacugauaagggca....................	27	1	Fm1
......................................................................uggaGggagaacugauaagggca....................	2	1	Fm1
......................................................................uUgacggagaacugauaagggca....................	4	1	Fm1
......................................................................uggacgUagaacugauaagggca....................	15	1	Fm1
......................................................................uggacggagaacugauaaUggca....................	8	1	Fm1
......................................................................uggacgCagaacugauaagggca....................	3	1	Fm1
......................................................................uggacgNagaacugauaagggca....................	6	1	Fm1
......................................................................ugAacggagaacugauaagggca....................	17	1	Fm1
......................................................................uggacggaAacugauaagggca....................	7	1	Fm1
......................................................................uggacggagaacugauaCgggca....................	189	1	Fm1
......................................................................uggacggagaacAgauaagggca....................	3	1	Fm1
......................................................................uggacCgagaacugauaagggca....................	4	1	Fm1
......................................................................uNgacggagaacugauaagggca....................	2	1	Fm1
......................................................................uggacggagaacugauaUgggca....................	195	1	Fm1
......................................................................uggacggUgaacugauaagggca....................	23	1	Fm1
......................................................................uggacggagaaAugauaagggca....................	2	1	Fm1
......................................................................uggacggagaacugauaagggcC....................	616	1	Fm1
......................................................................Cggacggagaacugauaagggca....................	5	1	Fm1
```

Star Mature

```
cauuugugacaugagaaguaaguuugucucguguccucuucacuuuuccauccuguucuguacucgagaacuggacggagaacugauaagggcauagggcagaccacaucaaca
......................................................................ugCacggagaacugauaagggca....................    4     1   Fm1
......................................................................uAgacggagaacugauaagggca....................    24    1   Fm1
......................................................................ugUacggagaacugauaagggca....................    12    1   Fm1
......................................................................uggacggagaacuUauaagggca....................    3     1   Fm1
......................................................................uggaUggagaacugauaagggca....................    16    1   Fm1
......................................................................uggCcggagaacugauaagggca....................    8     1   Fm1
......................................................................uggacggagaUcugauaagggca....................    1901  1   Fm1
......................................................................uggacggagaacugauaaggAca....................    12    1   Fm1
......................................................................uggacggagaacugaCaagggca....................    2     1   Fm1
......................................................................uggacggagUacugauaagggca....................    17    1   Fm1
......................................................................uggacggagaaGugauaagggca....................    1     1   Fm1
......................................................................uggacggagaacGgauaagggca....................    2     1   Fm1
......................................................................uggacggaUaacugauaagggca....................    9     1   Fm1
......................................................................uCgacggagaacugauaagggca....................    7     1   Fm1
......................................................................uggacggagaacugCuaagggca....................    2     1   Fm1
......................................................................uggacggagaacugauaagggcG....................    2275  1   Fm1
......................................................................uggacAgagaacugauaagggca....................    7     1   Fm1
......................................................................uggUcggagaacugauaagggca....................    4     1   Fm1
......................................................................ugUacggagaacugauaagggcau...................    1     1   Fm1
......................................................................uggacggagaacugauaaUggcau...................    1     1   Fm1
......................................................................uggacggagaacuUauaagggcau...................    3     1   Fm1
......................................................................Nggacggagaacugauaagggcau...................    1     1   Fm1
......................................................................uggacggagaacugauGagggcau...................    7     1   Fm1
......................................................................uggacggCgaacugauaagggcau...................    2     1   Fm1
......................................................................uggacggagaacugauUagggcau...................    9     1   Fm1
......................................................................uggacggagaacugauaaggUcau...................    1     1   Fm1
......................................................................uggUcggagaacugauaagggcau...................    1     1   Fm1
......................................................................uUgacggagaacugauaagggcau...................    2     1   Fm1
......................................................................uCgacggagaacugauaagggcau...................    1     1   Fm1
......................................................................uNgacggagaacugauaagggcau...................    1     1   Fm1
......................................................................uggacggUgaacugauaagggcau...................    2     1   Fm1
......................................................................uggacggagaacugauaCgggcau...................    10    1   Fm1
......................................................................uggacggagaacugauaagggcaC...................    138   1   Fm1
......................................................................uggGcggagaacugauaagggcau...................    1     1   Fm1
......................................................................Aggacggagaacugauaagggcau...................    1     1   Fm1
......................................................................uggacggagaacugauaagggcaA...................    139   1   Fm1
......................................................................uggacggagaacugauaagCgcau...................    1     1   Fm1
......................................................................uggaGggagaacugauaagggcau...................    2     1   Fm1
......................................................................uggacgUagaacugauaagggcau...................    1     1   Fm1
......................................................................uggacggagaacuAauaagggcau...................    2     1   Fm1
......................................................................uggacggagaacugauaagggUau...................    208   1   Fm1
......................................................................uggaAggagaacugauaagggcau...................    2     1   Fm1
......................................................................uggacggagaGcugauaagggcau...................    5     1   Fm1
......................................................................uggacggagaacugauCagggcau...................    12    1   Fm1
......................................................................uggacggagCacugauaagggcau...................    2     1   Fm1
......................................................................uggacggagaacugauaagggcaG...................    69    1   Fm1
......................................................................uggacggagaUcugauaagggcau...................    151   1   Fm1
......................................................................uggacggagaacugauaagggAau...................    832   1   Fm1
......................................................................uggacggagaacugauaGgggcau...................    51    1   Fm1
......................................................................uggacggagaacugauaUgggcau...................    8     1   Fm1
......................................................................uggacggagaacugauaagggcau...................    3686  0   Fm1
......................................................................uggacggagaacugauaagggcCu...................    95    1   Fm1
......................................................................uAgacggagaacugauaagggcau...................    2     1   Fm1
......................................................................uggacggagaacugauaagggcGu...................    30    1   Fm1
......................................................................uggacggagaacugauaagggcUua..................    30    1   Fm1
......................................................................uggacggagaacugauaagggcaua..................    2     0   Fm1
......................................................................uggacggagaacugauaagggcauU..................    654   1   Fm1
......................................................................uggacggagaacugauaagggAaua..................    4     1   Fm1
......................................................................uggacggagaacugauaagggcauG..................    1     1   Fm1
......................................................................uggacggagaacugauaagggcauC..................    2     1   Fm1
......................................................................uggacggagaacugauaagggcauUg.................    1     1   Fm1
......................................................................uggacggagaacugauaagggcauaU.................    15    1   Fm1
.......................................................................ggacggagaacugauaaU........................    16    1   Fm1
.......................................................................ggGcggagaacugauaag........................    1     1   Fm1
.......................................................................ggacggagGacugauaag........................    1     1   Fm1
.......................................................................ggacggagaacugauaUg........................    1     1   Fm1
.......................................................................ggacggaAaacugauaag........................    1     1   Fm1
.......................................................................ggacggagaacugauaag........................    21    0   Fm1
.......................................................................Ugacggagaacugauaag........................    1     1   Fm1
.......................................................................ggacggagaacugauaaA........................    8     1   Fm1
```

Star Mature

```
cauuugugacaugagaaguaaguuugucucguguccucuucacuuuuccauccuguCuguacucgagaacuggacggagaacugauaagggcauagggcagaccacaucaaca
.......................................................................ggacggagaaAugauaagg.......................	1	1	Fm1
.......................................................................ggacggagaacugauaagA.......................	4	1	Fm1
.......................................................................Ugacggagaacugauaagg.......................	2	1	Fm1
.......................................................................ggacggagaacugauaagU.......................	1	1	Fm1
.......................................................................ggacggagaacGgauaagg.......................	1	1	Fm1
.......................................................................ggacggagaacugauaagg.......................	60	0	Fm1
.......................................................................ggacggagaacugauaaggU......................	4	1	Fm1
.......................................................................ggacggagaacugauaaggg......................	73	0	Fm1
.......................................................................ggacggagaacugauaaggC......................	1	1	Fm1
.......................................................................Ugacggagaacugauaaggg......................	4	1	Fm1
.......................................................................ggacggagCacugauaaggg......................	1	1	Fm1
.......................................................................ggacCgagaacugauaaggg......................	1	1	Fm1
.......................................................................ggacggagaacugauGaggg......................	1	1	Fm1
.......................................................................ggacggagaaAugauaaggg......................	1	1	Fm1
.......................................................................ggacggagaacugauCaggg......................	1	1	Fm1
.......................................................................ggacggagaacugauaaggA......................	16	1	Fm1
.......................................................................ggacggagaacGgauaagggc.....................	1	1	Fm1
.......................................................................ggaAggagaacugauaagggc.....................	4	1	Fm1
.......................................................................ggacggagaaGugauaagggc.....................	1	1	Fm1
.......................................................................ggacggagCacugauaagggc.....................	16	1	Fm1
.......................................................................ggacggCgaacugauaagggc.....................	4	1	Fm1
.......................................................................ggacggagaaAugauaagggc.....................	2	1	Fm1
.......................................................................ggacggagaacugauaUgggc.....................	1	1	Fm1
.......................................................................ggacggagaCcugauaagggc.....................	3	1	Fm1
.......................................................................ggCcggagaacugauaagggc.....................	2	1	Fm1
.......................................................................ggacggagaacugauaaggAc.....................	1	1	Fm1
.......................................................................ggacggagaacugauUagggc.....................	1	1	Fm1
.......................................................................ggacggaAaacugauaagggc.....................	2	1	Fm1
.......................................................................gUacggagaacugauaagggc.....................	1	1	Fm1
.......................................................................ggacUgagaacugauaagggc.....................	1	1	Fm1
.......................................................................ggacggGgaacugauaagggc.....................	1	1	Fm1
.......................................................................ggacggagaacugauaagggA.....................	51	1	Fm1
.......................................................................ggacggaUaacugauaagggc.....................	1	1	Fm1
.......................................................................ggacggagaacugauaGgggc.....................	9	1	Fm1
.......................................................................ggacggagaacAgauaagggc.....................	1	1	Fm1
.......................................................................ggacggagaacugauaCgggc.....................	1	1	Fm1
.......................................................................ggaGggagaacugauaagggc.....................	1	1	Fm1
.......................................................................ggGcggagaacugauaagggc.....................	2	1	Fm1
.......................................................................ggacggagUacugauaagggc.....................	2	1	Fm1
.......................................................................ggacggUgaacugauaagggc.....................	1	1	Fm1
.......................................................................ggacggagaacugauCagggc.....................	1	1	Fm1
.......................................................................ggacgUagaacugauaagggc.....................	1	1	Fm1
.......................................................................ggacggagaacugUuaagggc.....................	1	1	Fm1
.......................................................................Ugacggagaacugauaagggc.....................	45	1	Fm1
.......................................................................ggacggagaacugaAaagggc.....................	2	1	Fm1
.......................................................................ggacggagaacugauaagAgc.....................	2	1	Fm1
.......................................................................ggacggagaacugauaaggCc.....................	4	1	Fm1
.......................................................................ggacggagaacugauaagggU.....................	12	1	Fm1
.......................................................................ggacggagaacugauaagggc.....................	1101	0	Fm1
.......................................................................ggacggagGacugauaagggc.....................	4	1	Fm1
.......................................................................ggacggagaacugauaagUgc.....................	2	1	Fm1
.......................................................................ggacggagaacugauaaggUc.....................	2	1	Fm1
.......................................................................ggacggagaacCgauaagggc.....................	3	1	Fm1
.......................................................................ggacggagaaUugauaagggc.....................	4	1	Fm1
.......................................................................ggCcggagaacugauaagggca....................	1	1	Fm1
.......................................................................ggacggagaacugauaagggcG....................	4	1	Fm1
.......................................................................ggacggagCacugauaagggca....................	1	1	Fm1
.......................................................................ggacggagaacugauaaCggca....................	1	1	Fm1
.......................................................................ggacggagaacugauaagggAa....................	4	1	Fm1
.......................................................................ggUcggagaacugauaagggca....................	1	1	Fm1
.......................................................................Ugacggagaacugauaagggca....................	1	1	Fm1
.......................................................................ggacggagaacGgauaagggca....................	1	1	Fm1
.......................................................................ggacggagaacugauaagggca....................	76	0	Fm1
.......................................................................ggacggagaUcugauaagggca....................	8	1	Fm1
.......................................................................ggacggCgaacugauaagggca....................	1	1	Fm1
.......................................................................ggacggagaaAugauaagggca....................	1	1	Fm1
.......................................................................ggacggagaacugauaagggUau...................	2	1	Fm1
.......................................................................ggacggagaacugauaagggcau...................	4	0	Fm1
.......................................................................ggacggagaacugauaagggcaC...................	1	1	Fm1
........................................................................gacggagaacugauaagggc.....................	4	0	Fm1
```

Star **Mature**

cauuugugacaugagaaguaaguuugucucguguccucuucacuuuuccauccugucuguacucgagaacuggacggagaacugauaagggcauagggcagaccacaucaaca			
....Uacggagaacugauaagggc.....................	3	1	Fm1
....cggagaacugauaagggc.....................	3	0	Fm1
....Uggagaacugauaagggc.....................	10	1	Fm1
..................................ccucuucacuuuuccaCccugu...	1	1	Fm2
..................................ccucuucacuuuuccauccugu...	15	0	Fm2
..................................ccucuucacuuuuccauccugucuguacucgagaac...	11	0	Fm2
..................................ccucuucacuuuuccauccugucuCuacucgagaac...	1	1	Fm2
....acuggacggagaacugaGaagg.......................	1	1	Fm2
....acuggacggagaacugauaagg.......................	13	0	Fm2
....cuggacggagaacugauU..........................	2	1	Fm2
....cuggacggagaacugaua..........................	9	0	Fm2
....cuggacggagaacugauaa.........................	17	0	Fm2
....cuggacggagaacugaCaag........................	2	1	Fm2
....cuggacggagaacugauaaA........................	3	1	Fm2
....cuggacggagaacugauaaU........................	11	1	Fm2
....cuggacggagaacugauaag........................	51	0	Fm2
....cuggacggagaacugauCag........................	1	1	Fm2
....cuggacggagaacugaGaagg.......................	4	1	Fm2
....cuggacggagaacugauaaAg.......................	6	1	Fm2
....cuAgacggagaacugauaagg.......................	1	1	Fm2
....cuggacggGgaacugauaagg.......................	1	1	Fm2
....cuggacggagaacugauaagg.......................	228	0	Fm2
....cuggacggagaacugauaagA.......................	6	1	Fm2
....cuggacggagaacugauaagU.......................	3	1	Fm2
....cuggacggagCacugauaagg.......................	1	1	Fm2
....cuggacggagaacugauGagg.......................	2	1	Fm2
....cuggacggagGacugauaagg.......................	1	1	Fm2
....cuggacggagaacugauaaggA......................	31	1	Fm2
....cuggacggagaacugauaaAgg......................	7	1	Fm2
....cuggacggagaacugaCaaggg......................	1	1	Fm2
....Uuggacggagaacugauaaggg......................	1	1	Fm2
....cuggacggagaacugauCaggg......................	2	1	Fm2
....cuggacggagaacugauaaggg......................	210	0	Fm2
....cuggacggagaacugauaaggU......................	45	1	Fm2
....cuggacggagaacugaGaaggg......................	1	1	Fm2
....cuggacggagaacugauaagAg......................	1	1	Fm2
....cuggacggagaacCgauaagggc.....................	1	1	Fm2
....cuggaUggagaacugauaagggc.....................	2	1	Fm2
....cuggacggagaacugauUagggc.....................	1	1	Fm2
....cuggacggagaacugaGaagggc.....................	2	1	Fm2
....cuggacggagaacugaCaagggc.....................	1	1	Fm2
....cuggacggagaacugauaagggc.....................	420	0	Fm2
....cuggacggagaacugauCagggc.....................	2	1	Fm2
....cuggacggagaaUugauaagggc.....................	4	1	Fm2
....cuggacggagaacugauaaUggc.....................	1	1	Fm2
....cuggacggagaacugaAaagggc.....................	2	1	Fm2
....cuggacggagaacugauaagggG.....................	2	1	Fm2
....cuggacggagCacugauaagggc.....................	1	1	Fm2
....cuggacggagaacugauaagggU.....................	23	1	Fm2
....cuggacggagaacugauaagggca....................	11	0	Fm2
....uggacggagaacugaAaa.........................	81	1	Fm2
....uggacggagaacugauGa.........................	3	1	Fm2
....uggacggagaacugauaC.........................	3	1	Fm2
....uggacggagaacugauaU.........................	23	1	Fm2
....uggacggagaacugauaa.........................	908	0	Fm2
....uggacggagaCcugauaa.........................	2	1	Fm2
....uggacggagaacugauCa.........................	1	1	Fm2
....uggacggaUaacugauaa.........................	1	1	Fm2
....uggacggagaacugauUa.........................	9	1	Fm2
....uggaUggagaacugauaa.........................	1	1	Fm2
....uggacggagaGcugauaa.........................	1	1	Fm2
....uggacggagaacugaGaa.........................	2	1	Fm2
....uggacggagaacugauaG.........................	12	1	Fm2
....uggaGggagaacugauaag........................	2	1	Fm2
....uUgacggagaacugauaag........................	1	1	Fm2
....uggacggagaacugauaaA........................	3153	1	Fm2
....uggacgUagaacugauaag........................	2	1	Fm2
....uggacggagaacugaCaag........................	3	1	Fm2
....uggacggagaacugauaGg........................	19	1	Fm2

```
                                  Star                                Mature
cauuugugacaugagaaguaaguuugucucguguccucuucacuuuuccauccugucuguacucgagaacuggacggagaacugauaagggcauagggcagaccacaucaaca
......................................................................ugCacggagaacugauaag........................	1	1	Fm2
......................................................................uggacggaUaacugauaag........................	1	1	Fm2
......................................................................uggacggagaacugauUag........................	9	1	Fm2
......................................................................uggacAgagaacugauaag........................	1	1	Fm2
......................................................................uggacggagCacugauaag........................	2	1	Fm2
......................................................................uggCcggagaacugauaag........................	1	1	Fm2
......................................................................Cggacggagaacugauaag........................	1	1	Fm2
......................................................................uggacggagaacugauaaU........................	1662	1	Fm2
......................................................................uggGcggagaacugauaag........................	1	1	Fm2
......................................................................uggacggagaacugauCag........................	15	1	Fm2
......................................................................uggacggagaacugauaCg........................	22	1	Fm2
......................................................................uggacggagaaUugauaag........................	2	1	Fm2
......................................................................uggUcggagaacugauaag........................	1	1	Fm2
......................................................................uggacggagaacugaAaag........................	5	1	Fm2
......................................................................uggacggUgaacugauaag........................	2	1	Fm2
......................................................................ugAacggagaacugauaag........................	3	1	Fm2
......................................................................uAgacggagaacugauaag........................	6	1	Fm2
......................................................................uggacggagaacugauaUg........................	17	1	Fm2
......................................................................uggaUggagaacugauaag........................	9	1	Fm2
......................................................................uggacggagaacugauaag........................	7644	0	Fm2
......................................................................uggacUgagaacugauaag........................	2	1	Fm2
......................................................................uggacggCgaacugauaag........................	1	1	Fm2
......................................................................uggacggagGacugauaag........................	9	1	Fm2
......................................................................Nggacggagaacugauaag........................	3	1	Fm2
......................................................................uggacggagaacugauGag........................	9	1	Fm2
......................................................................uggacggagaCcugauaag........................	8	1	Fm2
......................................................................Aggacggagaacugauaag........................	1	1	Fm2
......................................................................uggacggagaacCgauaag........................	1	1	Fm2
......................................................................uggacggagaacAgauaag........................	1	1	Fm2
......................................................................uggacggGgaacugauaag........................	3	1	Fm2
......................................................................uggacggagaGcugauaag........................	18	1	Fm2
......................................................................uggacggagaacuCauaag........................	2	1	Fm2
......................................................................uggacNgagaacugauaag........................	2	1	Fm2
......................................................................uggacggagUacugauaag........................	4	1	Fm2
......................................................................uggacggagaacugauaaC........................	7	1	Fm2
......................................................................uggacggagaacugauaagA.......................	1695	1	Fm2
......................................................................uggacggagaacugauaagC.......................	3	1	Fm2
......................................................................uggacggUgaacugauaagg.......................	12	1	Fm2
......................................................................uggacggagaacugauaagU.......................	534	1	Fm2
......................................................................uggacggagaacugauaUgg.......................	70	1	Fm2
......................................................................uggacggagaacugauGagg.......................	32	1	Fm2
......................................................................Nggacggagaacugauaagg.......................	11	1	Fm2
......................................................................uggacggaUaacugauaagg.......................	3	1	Fm2
......................................................................uggacggCgaacugauaagg.......................	4	1	Fm2
......................................................................uggacgCagaacugauaagg.......................	2	1	Fm2
......................................................................uggacggagaacugaGaagg.......................	2	1	Fm2
......................................................................uggacggaCaacugauaagg.......................	3	1	Fm2
......................................................................uggacggagaaUugauaagg.......................	4	1	Fm2
......................................................................ugUacggagaacugauaagg.......................	4	1	Fm2
......................................................................uggUcggagaacugauaagg.......................	4	1	Fm2
......................................................................uggacgNagaacugauaagg.......................	2	1	Fm2
......................................................................uggacggagCacugauaagg.......................	6	1	Fm2
......................................................................uggacggagaacCgauaagg.......................	8	1	Fm2
......................................................................ugCacggagaacugauaagg.......................	3	1	Fm2
......................................................................uggacggagaacugaCaagg.......................	1	1	Fm2
......................................................................Cggacggagaacugauaagg.......................	6	1	Fm2
......................................................................uggacggagaacugauUagg.......................	28	1	Fm2
......................................................................uggacggagaacugauCagg.......................	47	1	Fm2
......................................................................uggacggagGacugauaagg.......................	23	1	Fm2
......................................................................uggacggagUacugauaagg.......................	7	1	Fm2
......................................................................uggacggagaacugauaagg.......................	23066	0	Fm2
......................................................................uggacggGgaacugauaagg.......................	7	1	Fm2
......................................................................uggacggaAaacugauaagg.......................	1	1	Fm2
......................................................................uggacggagaacugauaCgg.......................	93	1	Fm2
......................................................................uggacggagaacugUuaagg.......................	4	1	Fm2
......................................................................uggacggagaacugGuaagg.......................	5	1	Fm2
......................................................................uggacggagaacGgauaagg.......................	1	1	Fm2
......................................................................uggacggagaacugauaGgg.......................	112	1	Fm2
......................................................................Aggacggagaacugauaagg.......................	16	1	Fm2
......................................................................uggacggagaaGugauaagg.......................	1	1	Fm2
```

Star Mature

cauuugugacaugagaaguaaguuugucucguguccucuucacuuuuccauccuguccuguacucgagaacuggacggagaacugauaagggcauagggcagaccacaucaaca

....uggacggagaacugauaaCg.......................	1	1	Fm2
....uggacNgagaacugauaagg.......................	1	1	Fm2
....uggaUggagaacugauaagg.......................	6	1	Fm2
....uggacggagaacuAauaagg.......................	4	1	Fm2
....uggacggagaacuCauaagg.......................	2	1	Fm2
....uggacggagaCcugauaagg.......................	68	1	Fm2
....uggacggagaGcugauaagg.......................	103	1	Fm2
....uAgacggagaacugauaagg.......................	10	1	Fm2
....uggacgAagaacugauaagg.......................	3	1	Fm2
....uggacggagaacuUauaagg.......................	5	1	Fm2
....uggacAgagaacugauaagg.......................	5	1	Fm2
....uggGcggagaacugauaagg.......................	3	1	Fm2
....uggacCgagaacugauaagg.......................	1	1	Fm2
....uggCcggagaacugauaagg.......................	5	1	Fm2
....ugAacggagaacugauaagg.......................	6	1	Fm2
....uggacgUagaacugauaagg.......................	1	1	Fm2
....uggacggagaacugauaaUg.......................	4	1	Fm2
....uggaGggagaacugauaagg.......................	1	1	Fm2
....uggacggagaacugauaaAg.......................	124	1	Fm2
....uggaAggagaacugauaagg.......................	3	1	Fm2
....uggacUgagaacugauaagg.......................	3	1	Fm2
....uUgacggagaacugauaagg.......................	9	1	Fm2
....uAgacggagaacugauaaggg......................	20	1	Fm2
....uggacggagaacugauaaggU......................	824	1	Fm2
....uggacggaAaacugauaaggg......................	2	1	Fm2
....uggacggUgaacugauaaggg......................	21	1	Fm2
....Cggacggagaacugauaaggg......................	8	1	Fm2
....uggacggagaacugauaagCg......................	2	1	Fm2
....uggUcggagaacugauaaggg......................	4	1	Fm2
....uggacggagaacugauaGggg......................	193	1	Fm2
....Gggacggagaacugauaaggg......................	4	1	Fm2
....uggacAgagaacugauaaggg......................	7	1	Fm2
....uggacggagaacugauaCggg......................	130	1	Fm2
....uggaUggagaacugauaaggg......................	8	1	Fm2
....uggacgAagaacugauaaggg......................	2	1	Fm2
....uggacggagaacugauaaggA......................	6724	1	Fm2
....ugUacggagaacugauaaggg......................	5	1	Fm2
....uggaAggagaacugauaaggg......................	2	1	Fm2
....uggacggagaacugGuaaggg......................	4	1	Fm2
....uggacggagaacugauaaggg......................	35348	0	Fm2
....uggacgNagaacugauaaggg......................	4	1	Fm2
....uggacgCagaacugauaaggg......................	3	1	Fm2
....uggacNgagaacugauaaggg......................	4	1	Fm2
....uggacgUagaacugauaaggg......................	15	1	Fm2
....uggacggagaacugaGaaggg......................	1	1	Fm2
....uggacggagaacugCuaaggg......................	2	1	Fm2
....uCgacggagaacugauaaggg......................	3	1	Fm2
....uggacCgagaacugauaaggg......................	1	1	Fm2
....uggacggagaGcugauaaggg......................	127	1	Fm2
....uggacggagaacugauGaggg......................	52	1	Fm2
....Aggacggagaacugauaaggg......................	22	1	Fm2
....uggaGggagaacugauaaggg......................	5	1	Fm2
....uggacggagaacugauUaggg......................	56	1	Fm2
....uggacggGgaacugauaaggg......................	4	1	Fm2
....uggacggagaacugaAaaggg......................	2	1	Fm2
....uggacggagaacugauaaAgg......................	3	1	Fm2
....uggacggagaacAgauaaggg......................	3	1	Fm2
....uggacggagaacugauaagAg......................	8	1	Fm2
....uggacggagaacuAauaaggg......................	3	1	Fm2
....Nggacggagaacugauaaggg......................	16	1	Fm2
....uggacggagaacuCauaaggg......................	2	1	Fm2
....ugAacggagaacugauaaggg......................	8	1	Fm2
....uggacggaUaacugauaaggg......................	5	1	Fm2
....ugCacggagaacugauaaggg......................	4	1	Fm2
....uggacggagaaAugauaaggg......................	1	1	Fm2
....uggacggagaacugaCaaggg......................	6	1	Fm2
....uggacUgagaacugauaaggg......................	9	1	Fm2
....uggacggagaacuUauaaggg......................	2	1	Fm2
....uggacggagaacugauaaUgg......................	13	1	Fm2
....uggacggagaaUugauaaggg......................	15	1	Fm2

```
                                                      Star                              Mature
cauuugugacaugagaaguaaguuugucucguguccucuucacuuuuccauccugucuguacucgagaacuggacggagaacugauaagggcauagggcagaccacaucaaca
......................................................................uggacggagaacCgauaaggg......................    7       1    Fm2
......................................................................uggacggagaaGugauaaggg......................    1       1    Fm2
......................................................................uggacggagGacugauaaggg......................    27      1    Fm2
......................................................................uggacggagaacugUuaaggg......................    6       1    Fm2
......................................................................uggacggagUacugauaaggg......................    16      1    Fm2
......................................................................uggCcggagaacugauaaggg......................    4       1    Fm2
......................................................................uggacggagaCcugauaaggg......................    67      1    Fm2
......................................................................uggacggagaacugauaaCgg......................    3       1    Fm2
......................................................................uggacggagaacugauaaggC......................    16      1    Fm2
......................................................................uUgacggagaacugauaaggg......................    10      1    Fm2
......................................................................uggacggagaacugauaagUg......................    9       1    Fm2
......................................................................uggacggagCacugauaaggg......................    8       1    Fm2
......................................................................uggacggagaacugauaNggg......................    1       1    Fm2
......................................................................uggacggCgaacugauaaggg......................    2       1    Fm2
......................................................................uggacggagaacugauaUggg......................    112     1    Fm2
......................................................................uggacggagaacugauCaggg......................    75      1    Fm2
......................................................................uggGcggagaacugauaaggg......................    5       1    Fm2
......................................................................Gggacggagaacugauaagggc.....................    7       1    Fm2
......................................................................uggacggagaacAgauaagggc.....................    7       1    Fm2
......................................................................ugCacggagaacugauaagggc.....................    28      1    Fm2
......................................................................uggacggagaacugauaCgggc.....................    1038    1    Fm2
......................................................................uggacggagaacugauaaggAc.....................    83      1    Fm2
......................................................................uggacggagaaUugauaagggc.....................    70      1    Fm2
......................................................................uggacggaAaacugauaagggc.....................    15      1    Fm2
......................................................................uggacNgagaacugauaagggc.....................    34      1    Fm2
......................................................................uggacgUagaacugauaagggc.....................    60      1    Fm2
......................................................................uggacggagaacugCuaagggc.....................    7       1    Fm2
......................................................................uggCcggagaacugauaagggc.....................    44      1    Fm2
......................................................................uggacggagaacugaCaagggc.....................    22      1    Fm2
......................................................................uggacggagaacugauaagggA.....................    13052   1    Fm2
......................................................................uggacggagaCcugauaagggc.....................    221     1    Fm2
......................................................................uggacggaCaacugauaagggc.....................    11      1    Fm2
......................................................................ugNacggagaacugauaagggc.....................    2       1    Fm2
......................................................................uggacggGgaacugauaagggc.....................    83      1    Fm2
......................................................................uggacggaUaacugauaagggc.....................    55      1    Fm2
......................................................................uggacAgagaacugauaagggc.....................    38      1    Fm2
......................................................................uggacggagaacugauaaggUc.....................    78      1    Fm2
......................................................................uggacggagaacugauaagAgc.....................    38      1    Fm2
......................................................................uggaGggagaacugauaagggc.....................    13      1    Fm2
......................................................................uggacggagaacugauaagCgc.....................    17      1    Fm2
......................................................................uggGcggagaacugauaagggc.....................    55      1    Fm2
......................................................................uUgacggagaacugauaagggc.....................    77      1    Fm2
......................................................................uggacggagaacugauaagUgc.....................    78      1    Fm2
......................................................................uggacCgagaacugauaagggc.....................    10      1    Fm2
......................................................................ugUacggagaacugauaagggc.....................    54      1    Fm2
......................................................................Aggacggagaacugauaagggc.....................    62      1    Fm2
......................................................................uggacggagaacugauaaCggc.....................    8       1    Fm2
......................................................................uggaAggagaacugauaagggc.....................    32      1    Fm2
......................................................................uggacUgagaacugauaagggc.....................    58      1    Fm2
......................................................................uggacggagaacugauGagggc.....................    490     1    Fm2
......................................................................uggacggagaacugUuaagggc.....................    40      1    Fm2
......................................................................uggacggagaacuAauaagggc.....................    64      1    Fm2
......................................................................uggacggagaacugaAaagggc.....................    7       1    Fm2
......................................................................uAgacggagaacugauaagggc.....................    133     1    Fm2
......................................................................uggacggagaacugauaagggU.....................    2043    1    Fm2
......................................................................ugAacggagaacugauaagggc.....................    70      1    Fm2
......................................................................uggacggagaacuCauaagggc.....................    16      1    Fm2
......................................................................uggacggagaacugauaagggc.....................    272144  0    Fm2
......................................................................uggacggagCacugauaagggc.....................    56      1    Fm2
......................................................................uggacggagaGcugauaagggc.....................    478     1    Fm2
......................................................................uggacggagaacugauCagggc.....................    583     1    Fm2
......................................................................Nggacggagaacugauaagggc.....................    143     1    Fm2
......................................................................uggacggagaaAugauaagggc.....................    5       1    Fm2
......................................................................uggaUggagaacugauaagggc.....................    84      1    Fm2
......................................................................uggacgAagaacugauaagggc.....................    32      1    Fm2
......................................................................uggacggagGacugauaagggc.....................    1032    1    Fm2
......................................................................uggacggagaacuUauaagggc.....................    30      1    Fm2
......................................................................uggacggagUacugauaagggc.....................    107     1    Fm2
......................................................................uggacggagaacugGuaagggc.....................    26      1    Fm2
......................................................................uggacggagaacugauaaUggc.....................    66      1    Fm2
```

```
                                  Star                                  Mature
cauuugugacaugagaaguaaguuugucucguguccucuucacuuuuccauccugucuguacucgagaacuggacggagaacugauaagggcauagggcagaccacaucaaca
......................................................................uNgacggagaacugauaagggc.....................	3	1	Fm2
......................................................................uggacggagaacugauaGgggc.....................	6080	1	Fm2
......................................................................uggacggagaacugauUagggc.....................	345	1	Fm2
......................................................................uCgacggagaacugauaagggc.....................	38	1	Fm2
......................................................................uggacggagaacCgauaagggc.....................	29	1	Fm2
......................................................................uggacggCgaacugauaagggc.....................	50	1	Fm2
......................................................................Cggacggagaacugauaagggc.....................	37	1	Fm2
......................................................................uggacggagaaGugauaagggc.....................	6	1	Fm2
......................................................................uggUcggagaacugauaagggc.....................	43	1	Fm2
......................................................................uggacggagaacugaGaagggc.....................	9	1	Fm2
......................................................................uggacgCagaacugauaagggc.....................	27	1	Fm2
......................................................................uggacggagaacugauaagggG.....................	452	1	Fm2
......................................................................uggacggagaacugauaUgggc.....................	904	1	Fm2
......................................................................uggacggUgaacugauaagggc.....................	161	1	Fm2
......................................................................uggacgNagaacugauaagggc.....................	16	1	Fm2
......................................................................uggacggagaacGgauaagggc.....................	7	1	Fm2
......................................................................uggacggagaacugauaaggCc.....................	31	1	Fm2
......................................................................uggacggagaacugauaaAggc.....................	40	1	Fm2
......................................................................uggacAgagaacugauaagggca....................	4	1	Fm2
......................................................................uggacggagaacugauUagggca....................	32	1	Fm2
......................................................................uggacggCgaacugauaagggca....................	1	1	Fm2
......................................................................uggacggagaCcugauaagggca....................	6	1	Fm2
......................................................................uUgacggagaacugauaagggca....................	7	1	Fm2
......................................................................uggacgAagaacugauaagggca....................	3	1	Fm2
......................................................................uggacggagaacugauGagggca....................	65	1	Fm2
......................................................................uggacggagaUcugauaagggca....................	768	1	Fm2
......................................................................uggGcggagaacugauaagggca....................	6	1	Fm2
......................................................................uggacggagaacugaGaagggca....................	1	1	Fm2
......................................................................uggacggagaacugauaaggAca....................	6	1	Fm2
......................................................................uggacggagaacuAauaagggca....................	4	1	Fm2
......................................................................uggacggagUacugauaagggca....................	12	1	Fm2
......................................................................uggacggagaacugauaaAggca....................	4	1	Fm2
......................................................................uggacggagaacugauaagggUa....................	99	1	Fm2
......................................................................uggacggaCaacugauaagggca....................	1	1	Fm2
......................................................................uggacggagGacugauaagggca....................	29	1	Fm2
......................................................................uggacggGgaacugauaagggca....................	7	1	Fm2
......................................................................Nggacggagaacugauaagggca....................	17	1	Fm2
......................................................................uggacggagaacugaAaagggca....................	2	1	Fm2
......................................................................uggacggagaacCgauaagggca....................	3	1	Fm2
......................................................................uggacggaAaacugauaagggca....................	1	1	Fm2
......................................................................uAgacggagaacugauaagggca....................	20	1	Fm2
......................................................................uggacggagaacugauCagggca....................	49	1	Fm2
......................................................................uggacgUagaacugauaagggca....................	7	1	Fm2
......................................................................Gggacggagaacugauaagggca....................	1	1	Fm2
......................................................................uggacggagaacuUauaagggca....................	1	1	Fm2
......................................................................uggacggagaacugauaagggAa....................	701	1	Fm2
......................................................................uggacNgagaacugauaagggca....................	3	1	Fm2
......................................................................uggacggagaacugauaagggcC....................	179	1	Fm2
......................................................................uggacggUgaacugauaagggca....................	8	1	Fm2
......................................................................uggacggagaacugauaaCggca....................	1	1	Fm2
......................................................................uggaAggagaacugauaagggca....................	2	1	Fm2
......................................................................uCgacggagaacugauaagggca....................	3	1	Fm2
......................................................................uggacggagaacugauaaggUca....................	9	1	Fm2
......................................................................uggacggagaGcugauaagggca....................	39	1	Fm2
......................................................................uggacggagaacugauaaggCca....................	3	1	Fm2
......................................................................uggUcggagaacugauaagggca....................	5	1	Fm2
......................................................................Aggacggagaacugauaagggca....................	2	1	Fm2
......................................................................uggacggagaacugauaUgggca....................	89	1	Fm2
......................................................................uggaGggagaacugauaagggca....................	3	1	Fm2
......................................................................uggacggagCacugauaagggca....................	7	1	Fm2
......................................................................uggacggagaacugGuaagggca....................	2	1	Fm2
......................................................................uggCcggagaacugauaagggca....................	3	1	Fm2
......................................................................uggacggagaacuCauaagggca....................	4	1	Fm2
......................................................................uggacggagaacugauaCgggca....................	110	1	Fm2
......................................................................uggacCgagaacugauaagggca....................	1	1	Fm2
......................................................................uggacggagaacugauaagggca....................	25517	0	Fm2
......................................................................uggacggagaacugauaagggcG....................	874	1	Fm2
......................................................................ugAacggagaacugauaagggca....................	4	1	Fm2
......................................................................uggacggagaacugauaaUggca....................	5	1	Fm2
......................................................................uggacggagaacugauaGgggca....................	273	1	Fm2
```

Star **Mature**

```
cauuugugacaugagaaguaaguuugucucguguccucuucacuuuuccauccuguCuguacucgagaacuggacggagaacugauaagggcauagggcagaccacaucaaca
......................................................................uggacggagaacAgauaagggca....................	1	1	Fm2
......................................................................uggacggagaacugauaagUgca....................	8	1	Fm2
......................................................................uggacggaUaacugauaagggca....................	4	1	Fm2
......................................................................uggacUgagaacugauaagggca....................	8	1	Fm2
......................................................................uggacggagaacugUuaagggca....................	5	1	Fm2
......................................................................uggaUggagaacugauaagggca....................	9	1	Fm2
......................................................................uggacggagaacugauaagAgca....................	4	1	Fm2
......................................................................Cggacggagaacugauaagggca....................	6	1	Fm2
......................................................................ugUacggagaacugauaagggca....................	5	1	Fm2
......................................................................uggacggagaaUugauaagggca....................	5	1	Fm2
......................................................................uggacggagaacugauaaAggcau...................	1	1	Fm2
......................................................................uUgacggagaacugauaagggcau...................	1	1	Fm2
......................................................................uAgacggagaacugauaagggcau...................	1	1	Fm2
......................................................................Nggacggagaacugauaagggcau...................	2	1	Fm2
......................................................................uggacggagaacugauaagggcau...................	1743	0	Fm2
......................................................................uggacggagaacugauaagUgcau...................	1	1	Fm2
......................................................................uggacggagaacugauCagggcau...................	5	1	Fm2
......................................................................uggacggagaacugauUagggcau...................	2	1	Fm2
......................................................................uggacggagaacugauaCgggcau...................	6	1	Fm2
......................................................................uggacggagGacugauaagggcau...................	1	1	Fm2
......................................................................uggacggagaacugauaagggUau...................	267	1	Fm2
......................................................................uggacggagaGcugauaagggcau...................	2	1	Fm2
......................................................................uggacggagaacugauaagggAau...................	602	1	Fm2
......................................................................uggacUgagaacugauaagggcau...................	1	1	Fm2
......................................................................uggacggagaUcugauaagggcau...................	123	1	Fm2
......................................................................uggacgUagaacugauaagggcau...................	2	1	Fm2
......................................................................uggGcggagaacugauaagggcau...................	1	1	Fm2
......................................................................uggacggagaacugauaaCggcau...................	1	1	Fm2
......................................................................uggacggagaacugauaagggcGu...................	5	1	Fm2
......................................................................uggacggagaacugauaUgggcau...................	8	1	Fm2
......................................................................uggacggagaacugauaagggcaA...................	67	1	Fm2
......................................................................uggacggagaacuCauaagggcau...................	1	1	Fm2
......................................................................uggacggagaCcugauaagggcau...................	2	1	Fm2
......................................................................uggacggagaacugauaagggcaG...................	4	1	Fm2
......................................................................ugCacggagaacugauaagggcau...................	1	1	Fm2
......................................................................uCgacggagaacugauaagggcau...................	1	1	Fm2
......................................................................uggacggagaacugauaagggcCu...................	13	1	Fm2
......................................................................uggacggagaacugauaGgggcau...................	35	1	Fm2
......................................................................uggacggagaacugauGagggcau...................	3	1	Fm2
......................................................................uggacggagaacugauaagggcUua..................	3	1	Fm2
......................................................................uggacggagaacugauaagggcauU..................	383	1	Fm2
......................................................................uggacggagaacugauaagggcauC..................	12	1	Fm2
......................................................................uggacggagaacugauaagggcauUg.................	1	1	Fm2
......................................................................uggacggagaacugauaagggcauaU.................	14	1	Fm2
.......................................................................ggacggagaacugauaag........................	3	0	Fm2
.......................................................................ggacggagaacugauaaU........................	5	1	Fm2
.......................................................................ggacggagaacugauaaA........................	2	1	Fm2
.......................................................................ggacggagGacugauaagg.......................	1	1	Fm2
.......................................................................ggacggagaacGgauaagg.......................	1	1	Fm2
.......................................................................ggacggagaacugauaagA.......................	2	1	Fm2
.......................................................................ggacggagaacugauaagg.......................	30	0	Fm2
.......................................................................ggacggagaacugauaagU.......................	1	1	Fm2
.......................................................................ggacggagaacugauaGgg.......................	1	1	Fm2
.......................................................................Ugacggagaacugauaagg.......................	2	1	Fm2
.......................................................................ggacggagaacugauaagUg......................	1	1	Fm2
.......................................................................Ugacggagaacugauaaggg......................	5	1	Fm2
.......................................................................ggacggagaacugauaaggA......................	8	1	Fm2
.......................................................................ggacggagaacugauaagAg......................	1	1	Fm2
.......................................................................ggacggagaacugauaaggg......................	39	0	Fm2
.......................................................................ggacggaAaacugauaagggc.....................	1	1	Fm2
.......................................................................ggacgUagaacugauaagggc.....................	1	1	Fm2
.......................................................................ggacggagaacugauaGgggc.....................	6	1	Fm2
.......................................................................Agacggagaacugauaagggc.....................	1	1	Fm2
.......................................................................ggacggagaacugauaagggA.....................	22	1	Fm2
.......................................................................ggacggagaacugauaaggCc.....................	1	1	Fm2
.......................................................................ggacggagaacuUauaagggc.....................	1	1	Fm2
.......................................................................ggacggagaacugauaagggc.....................	191	0	Fm2
.......................................................................ggacggagaaUugauaagggc.....................	2	1	Fm2
.......................................................................ggacggUgaacugauaagggc.....................	1	1	Fm2
.......................................................................ggacggagCacugauaagggc.....................	3	1	Fm2
```

```
                                  Star                               Mature
cauuugugacaugagaaguaaguuugucucguguccucuucacuuuuccauccugucuguacucgagaacuggacggagaacugauaagggcauagggcagaccacaucaaca
.......................................................................ggacggagaaAugauaagggc.....................   1    1    Fm2
.......................................................................ggacggNgaacugauaagggc.....................   1    1    Fm2
.......................................................................Ugacggagaacugauaagggc.....................   13   1    Fm2
.......................................................................ggacggagaacugauaagCgc.....................   1    1    Fm2
.......................................................................ggacggagUacugauaagggc.....................   1    1    Fm2
.......................................................................ggacggagaacugauaaggAc.....................   3    1    Fm2
.......................................................................ggacggagaacuAauaagggc.....................   2    1    Fm2
.......................................................................ggacggagaacugauaagggU.....................   1    1    Fm2
.......................................................................ggacggagaUcugauaagggca....................   5    1    Fm2
.......................................................................ggacggagaacugauaagggcG....................   1    1    Fm2
.......................................................................ggacggagaaAugauaagggca....................   1    1    Fm2
.......................................................................ggacggagaacugauaagggca....................   31   0    Fm2
.......................................................................ggacggagaGcugauaagggcau...................   1    1    Fm2
.......................................................................ggacggagaacugauaagggcau...................   2    0    Fm2
........................................................................gacggagaacugauaagg.......................   1    0    Fm2
..........................................................................cggagaacugauaagggc.....................   5    0    Fm2
..........................................................................Uggagaacugauaagggc.....................   1    1    Fm2
```
